# Toward Improving the Selectivity of Organic Halide Electrocarboxylation with Mechanistically Informed Solvent Selection

**DOI:** 10.1021/jacs.2c10561

**Published:** 2023-01-10

**Authors:** Nathan Corbin, Glen P. Junor, Thu N. Ton, Rachel J. Baker, Karthish Manthiram

**Affiliations:** † Department of Chemical Engineering, Massachusetts Institute of Technology, 77 Massachusetts Avenue, Cambridge, Massachusetts02139, United States; ‡ Division of Chemistry and Chemical Engineering, California Institute of Technology, Pasadena, California91125, United States

## Abstract

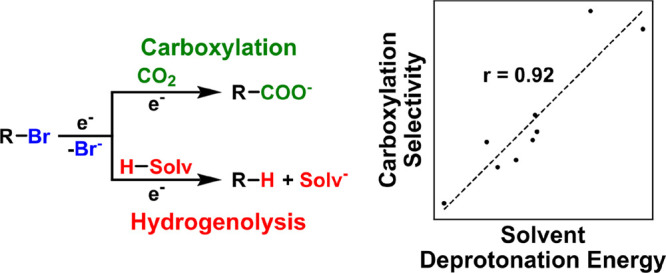

The use of a liquid electrolyte is nearly ubiquitous in electrosynthetic systems and can have a significant impact on the selectivity and efficiency of electrochemical reactions. Solvent selection is thus a key step during optimization, yet this selection process usually involves trial-and-error. As a step toward more rational solvent selection, this work examines how the electrolyte solvent impacts the selectivity of electrocarboxylation of organic halides. For the carboxylation of a model alkyl bromide, hydrogenolysis is the primary side reaction. Isotope-labeling studies indicate the hydrogen atom in the hydrogenolysis product comes solely from the aprotic electrolyte solvent. Further mechanistic studies reveal that under synthetically relevant electrocarboxylation conditions, the hydrogenolysis product is formed via deprotonation of the solvent. Guided by these mechanistic findings, a simple computational descriptor based on the free energy to deprotonate a solvent molecule was shown to correlate strongly with carboxylation selectivity, overcoming limitations of traditional solvent descriptors such as p*K*_a_. Through careful mechanistic analysis surrounding the role of the solvent, this work furthers the development of selective electrocarboxylation systems and more broadly highlights the benefits of such analysis to electrosynthetic reactions.

## Introduction

Synthetic electro-organic chemistry has experienced a surge in popularity due to its chemoselectivity stemming from precise potential control and mild conditions coupled with renewable energy compatibility.^1−6^ Electro-organic systems generally use liquid electrolytes comprising an ionic salt dissolved in a molecular solvent. By mole number, solvent molecules are the major species in most electrolytes for electro-organic synthesis, so the reactive and solvating properties of the solvent can have a profound influence on the rates and selectivities of electrochemical reactions. Choosing an appropriate solvent for an electrochemical reaction is crucial and can be facilitated by having appropriate solvent selection guidelines that reduce the amount of trial-and-error experimentation needed.

All liquid-phase reaction development requires careful solvent selection to ensure compatibility with reactants, products, and additives. At the outset, typical considerations include the coordinating ability, proticity, and possibly p*K*_a_ of the solvent. For electro-organic reactions, an additional consideration is the electrochemical stability window of the solvent, typically measured with an inert supporting electrolyte.^7,8^ These initial considerations can narrow the scope of solvents to test, but the exact role of the solvent in a reaction may not be obvious at the outset. As with conventional synthesis, numerous electrosynthetic studies have observed important product selectivity changes induced by the electrolyte solvent choice,^1,9−12^ many of which have been ascribed to various nonreactive roles including selectively changing oxidation potentials,^13^ acting as a mediator,^14^ modifying nucleophilicity,^15^ and coordinating ionic intermediates.^16^ A number of studies have shown that solvent molecules can also be reactive in electrochemical systems, which can be desirable for achieving certain types of products.^17,18^ For electrochemical systems, the application of a voltage can enable the creation of intermediates that are much more reactive than starting materials or products, complicating the solvent selection process. Thus, obtaining mechanistic understanding of how the solvent impacts reaction outcomes is an important task to enable proper solvent choice.

An important class of electro-organic reactions is reductive cross-coupling of organic halides with electrophiles. These transformations can generate new carbon–carbon^19,20^ or carbon–heteroatom^21^ bonds—both desirable transformations in industrial and synthetic organic chemistry. Protic solvents have been found to accelerate the reduction of many types of carbon–halogen bonds on catalytic electrodes^22,23^ and can even alter the reaction mechanism relative to that in an aprotic solvent.^24^ In these cases, protic solvents accelerate the hydrogenolysis of the carbon–halogen bond, which is usually undesirable for organic synthesis, although site-specific deuteration with D_2_O is one promising application.^25^ Among aprotic solvents, cleavage rates of carbon–halogen bonds have been correlated to the Lewis acidity of the solvent, which impacts its ability to solubilize the halogen anion byproduct.^26−29^ While these studies examined how the solvent impacts the rates and mechanism of electrochemical carbon–halogen bond cleavage, understanding of how the solvent affects the product selectivity of electrochemical cross-coupling reactions involving organic halides is lacking.

**Scheme 1:**
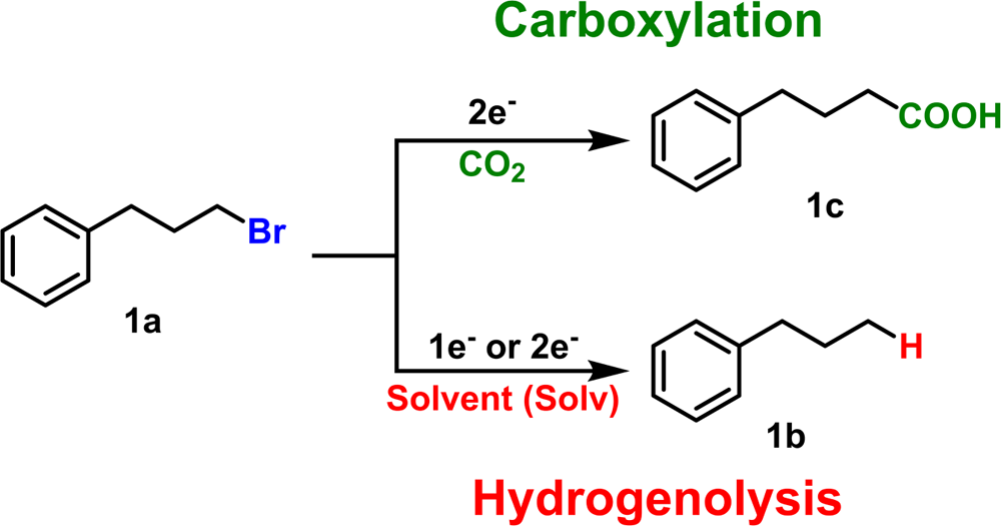
Competition between Carboxylation and Hydrogenolysis for the Model Alkyl Halide 1-Bromo-3-phenylpropane (**1a**)

To probe the role of the solvent on the selectivity of carbon–carbon bond formation, the electrochemical carboxylation of organic halides with carbon dioxide (CO_2_) is used as a model reaction (Scheme 1). This reaction scheme is promising because it can use sustainable energy (renewable electricity) and an abundant, renewable C_1_ carbon source (CO_2_) to construct a wide variety of valuable carboxylic acids.^30−32^ The use of applied potential can also eliminate the need for highly reactive organometallic reagents^5^ and can enable precise control over kinetic driving forces, leading to improved functional group tolerance.^33^ Although electrocarboxylation can be inherently more selective than traditional organometallic reagents, it can be plagued by the undesirable electrochemical hydrogenolysis of the carbon–halogen bond. Prior work has suggested that the selectivity of electrocarboxylation over hydrogenolysis can depend rather strongly on the choice of aprotic solvent,^33^ motivating an in-depth mechanistic study into the role(s) of the solvent. In this work, the aprotic solvent is shown to provide the hydrogen atoms for the hydrogenolysis side product during electrocarboxylation. The mechanism of the hydrogenolysis side reaction is elucidated with respect to the aprotic solvent, revealing that deprotonation of the solvent is the dominant pathway toward the hydrogenolysis product under synthetically relevant conditions for making carboxylic acids. These results are used to construct a computational molecular descriptor for solvents that correlates strongly with carboxylation selectivity and outperforms standard solvent descriptors from the literature such as p*K*_a_.

## Results and Discussion

### Origin of the Hydrogenolysis Product

A number of possible sources of hydrogen atoms and protons exist in typical electrocarboxylation electrolytes, including the solvent molecules, electrolyte ions such as tetra-*n*-butylammonium (TBA), trace amounts of water, and even the substrate itself. Trace water can be particularly reactive because protic solvents are known to accelerate reductive cleavage of carbon–halogen bonds for many types of organic halides.^22,23^ To identify the source of hydrogen atoms in the hydrogenolysis product, carboxylation reactions were conducted in both deuterated dimethyl sulfoxide (DMSO-*d*_6_) and deuterated acetonitrile (MeCN-*d*_3_), and the fraction of hydrogenolysis product containing deuterium was quantified. Both solvents were distilled and dried over 3 Å molecular sieves to reduce the chances of impurities, especially water, in the solvents influencing the results (see the Supporting Information). As a model organic halide, 1-bromo-3-phenylpropane (**1a**) was used because the rates of carboxylation and hydrogenolysis are comparable under electrochemical conditions.^33^ Electrochemical experiments were conducted in a single-compartment cell with a sacrificial aluminum anode and a silver cathode. Bromide ions were found to be necessary to enable oxidation of the aluminum anode, so a mixture of TBA-BF_4_ and TBA-Br was used (see the Supporting Information).

**Figure 1:**
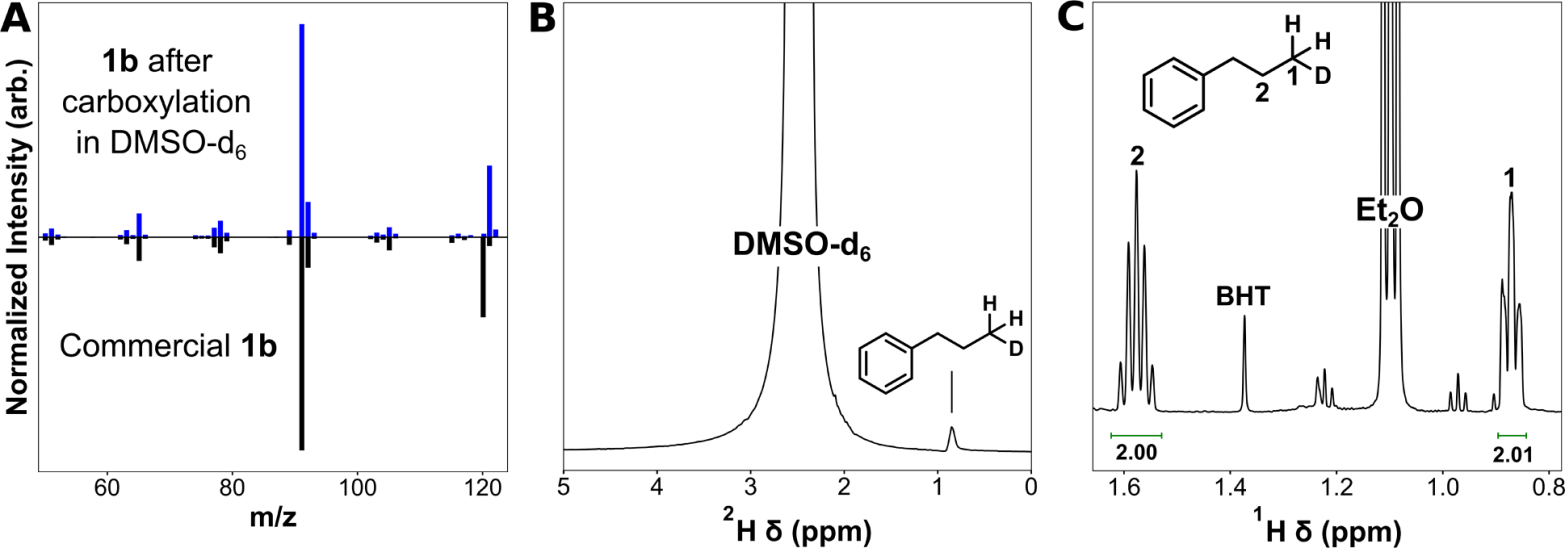
Evidence that hydrogen atoms from the solvent end up in the hydrogenolysis product during carboxylation. (A) Mass spectrum of the hydrogenolysis product **1b** after carboxylation in DMSO-*d*_6_ showing a terminal −CDH_2_ fragment. (B) ^2^H NMR spectrum showing a signal for deuterium in the aliphatic region. Reaction solvent (DMSO-*d*_6_) is indicated. (C) ^1^H NMR spectrum of **1b** quantifying the fraction of deuterium by the ratio of protons at the terminal carbon (1) to the internal carbon (2). Residual work-up solvent (Et_2_O) and stabilizer (BHT) are indicated. Reaction conditions: silver cathode, aluminum anode, undivided cell, −5 mA/cm^2^ for 2 h, 0.1 M TBA-BF_4_, 0.1 M **1a**, 25 mM MgBr_2_, 20 sccm of CO_2_, DMSO-*d*_6_.

The hydrogenolysis of **1a** produces the hydrocarbon product *n*-propylbenzene (**1b**). In a deuterated solvent with otherwise protic compounds, either a hydrogen or deuterium atom can replace the bromine in **1a**. A combination of nuclear magnetic resonance (NMR) spectroscopy and mass spectrometry (MS) was used to confirm the replacement of the carbon–bromine bond with a carbon–deuterium bond. The mass spectrum of **1b** after carboxylation in DMSO-*d*_6_ shows the primary mass peak increases by one relative to that of a commercial standard of **1b**, consistent with the incorporation of a single deuterium nucleus into the product (Figure 1A). The increased mass only persists for the first set of peaks, indicating that the deuterium is on the terminal carbon of the propyl chain. The presence of deuterium on the terminal carbon is also confirmed by ^2^H NMR which shows a deuterium peak in the aliphatic region (Figure 1B). Additional triplet splitting on the terminal carbon protons by deuterium can also be observed by ^1^H NMR (Figure S4). These spectroscopic results collectively prove a significant amount of **1b** contains a deuterium at the terminal carbon after carboxylation in a deuterated solvent.

To probe the origin of the hydrogen atom in **1b** more thoroughly, electrocarboxylation experiments were performed (1) in two different anhydrous, deuterated solvents (DMSO-*d*_6_ and MeCN-*d*_3_), (2) at both low and high conversion of **1a**, and (3) at constant current and constant potential. The deuterated fraction was quantified either from the ratio of the *m*/*z* 121 and 120 peaks from MS or from the ratio of the integrated proton signals at the terminal and internal carbon atoms (Figure 1C). Under all conditions, the vast majority of the hydrogenolysis product contains deuterium (Table 1). On the basis of these results, solvent hydrogen atoms are the primary source of the hydrogenolysis product under synthetically relevant electrocarboxylation conditions.

**Table 1:** Quantification of Deuterated **1b** Fractions after Carboxylation in Deuterated Solvents^a^

solvent	conditions	percentage of deuterated **1b**
DMSO-*d*_6_	–5 mA/cm^2^ for 2 h, 100 mM **1a**	>98.7 (^1^H NMR)
MeCN-*d*_3_	–5 mA/cm^2^ for 2 h, 100 mM **1a**	>99.6 (^1^H NMR)
MeCN-*d*_3_	–2.21 V^b^ until 8 C passed, 20 mM **1a**	103 ± 5 (MS)
MeCN-*d*_3_	–2.37 V^b^ until 8 C passed, 20 mM **1a**	105 ± 5 (MS)

a Reaction conditions for constant current experiments: silver cathode, aluminum anode, undivided cell, 0.1 M TBA-BF_4_, 25 mM MgBr_2_, 20 sccm CO_2_. Reaction conditions for constant potential experiments: silver cathode, aluminum anode, undivided cell, 90 mM TBA-BF_4_, 10 mM TBA-Br, 20 sccm CO_2_. Solvents and reaction times are specified above. Deuteration percentages from ^1^H NMR are given as lower bounds due to the presence of impurity peaks around the terminal carbon proton peak, while deuteration percentages from MS include a ±5% absolute error bound from the calibration curve (see the Supporting Information for more details).

b Voltages referenced to Me_10_Fc^0/+^.

### Hydrogenolysis Mechanism under Electrocarboxylation Conditions

**Scheme 2:**
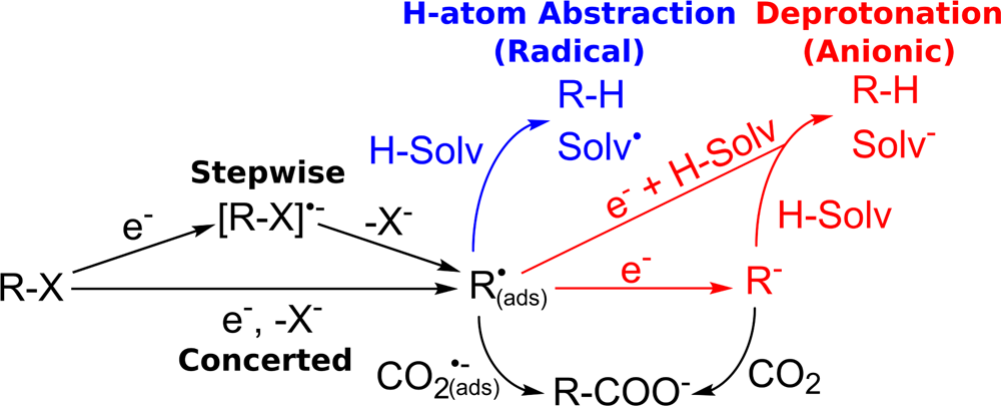
Possible Mechanistic Pathways during Electrocarboxylation of Organic Halides The hydrogenolysis routes involving hydrogen-atom (H-atom) abstraction and deprotonation are highlighted. Solvent deprotonation could feasibly occur via a concerted or stepwise pathway. H-Solv = electrolyte solvent, R-X = organic halide. Species that may or may not be adsorbed to the electrode are denoted with a subscript (Ads).

In the case of carboxylation and other cross-coupling reactions involving organic halides, hydrogenolysis products are not desirable. However, studying the hydrogenolysis mechanism can provide insights into which properties of the solvent control the predominance of this pathway. For the electrochemical reduction of carbon–halogen bonds, the first step is widely accepted to involve a concerted or stepwise cleavage of the carbon–halogen bond, forming a halide anion and a carbon radical (Scheme 2).^34−37^ The use of catalytic electrodes such as silver^38^ and substrates without low-lying π* orbitals^37^ favors the concerted pathway, as is the case in this work. Once the carbon–halogen bond is cleaved, hydrogenation of the organic radical intermediate can proceed via either radical hydrogen abstraction (1e^–^ process) or anionic deprotonation (2e^–^ process).^39^ Carboxylation may also proceed from either the radical or anionic intermediate in a 2e^–^ process. To facilitate rational solvent selection, the predominant hydrogenolysis pathway needs to be identified because each pathway involves a different reactive property of the solvent.

**Figure 2:**
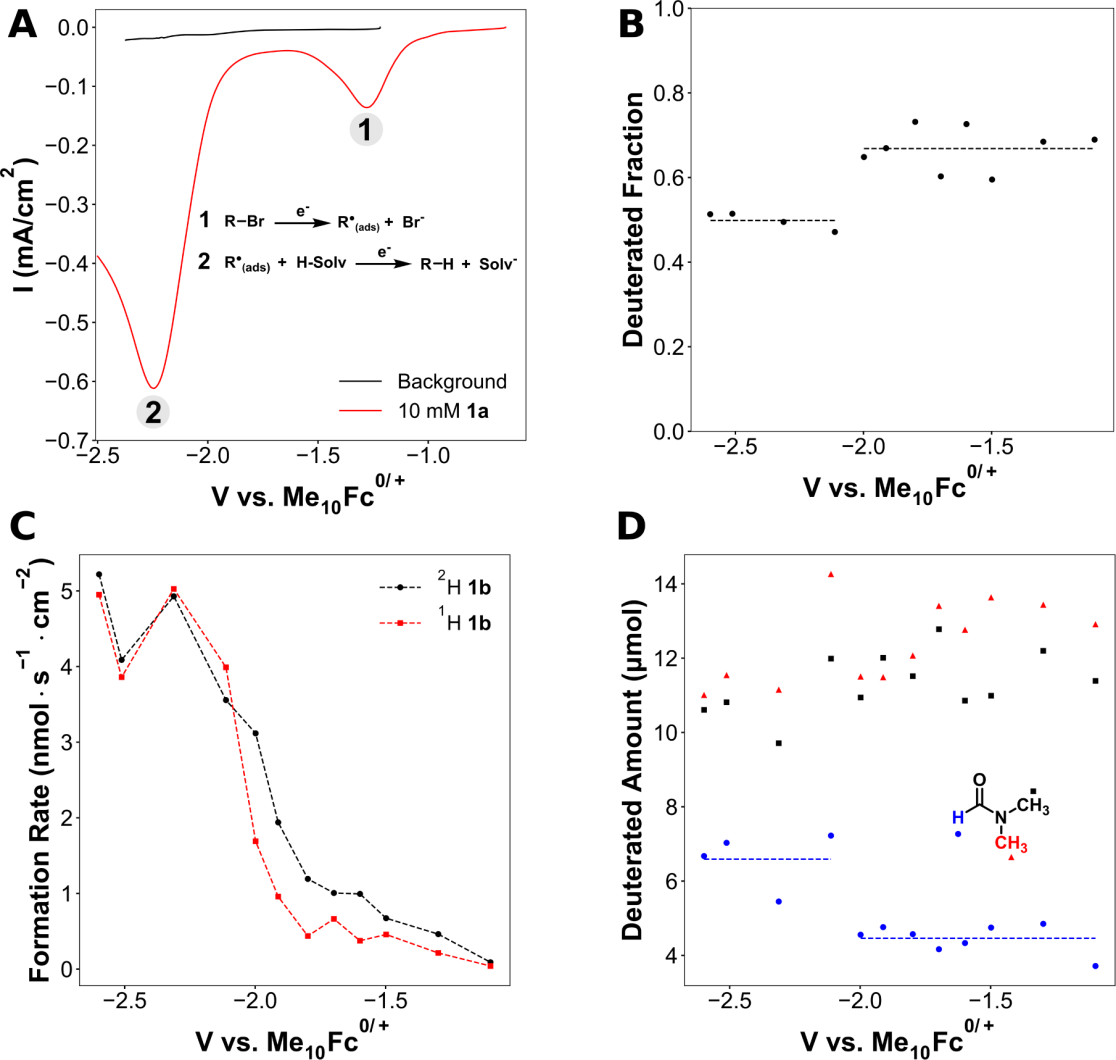
Reduction of **1a** in the presence of EtOD in DMF at various potentials. (A) Linear sweep voltammograms (LSVs) at 10 mV/s in DMF with and without 10 mM **1a**. Electrolyte: 90 mM TBA-BF_4_ and 10 mM TBA-Br. (B) Fraction of deuterated hydrogenolysis product (**1b**) as a function of potential. Dashed lines indicate the average deuterated fraction for each group of points. (C) Formation rates of protonated and deuterated **1b** as a function of potential. Dashed lines are to guide the eye. (D) Amount of deuterium incorporated into DMF as a function of potential. Dashed lines indicate the average amount of deuterium incorporation at the formyl position for each group of points. Reaction conditions for panels B–D: 20 mM **1a**, 90 mM TBA-BF_4_, 10 mM TBA-Br, 20 sccm N_2_, DMF, and 400 mM EtOD. Experiments were run for either 4 C or 1 h, whichever condition was met first. Me_10_Fc = decamethylferrocene. H-Solv = solvent.

Linear sweep voltammetry indicates the presence of two electrochemical reactions during the reduction of **1a** on silver in DMF (Figure 2A). The first peak likely corresponds to the reductive cleavage of the carbon–bromine bond to discharge a bromide anion and form an adsorbed organic species or an organoradical. The presence of an organic radical intermediate was confirmed by the formation of a coupling product when the radical trap 4-vinylanisole was added (Figures S20–S23).

The second cathodic peak has a much greater peak current than that of the first. It displays a dependence on the concentration of **1a**, and the peak current is proportional to the square root of the scan rate (Figure S7). These observations are consistent with the second peak arising from transport limitations. The reduction of organic (R^•^_(ads)_) or solvent radical species (Solv^•^_(ads)_) could be responsible for the current at the second reduction peak:
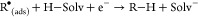

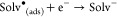
The presence of possibly adsorbed solvent species could arise from hydrogen atom abstraction by the organic species. Both species would only be generated after the initial one-electron reduction of **1a**, so the reduction of both would also become limited by mass transport of **1a**. Distinguishing between these two processes is key to understanding the hydrogenolysis mechanism.

To clarify which reaction is occurring at the second cathodic peak, the reduction of **1a** was conducted in the presence of deuterated ethanol (EtOD). This deuterated additive was selected because the −OD group should be susceptible to deprotonation but not to deuterium abstraction.^39^ To avoid the formation of adsorbed deuterium, which could react with radical intermediates, potentials were kept more positive of −2.6. V vs Me_10_Fc^0/+^ (all potentials in this work are referenced to decamethylferrocene), which is the observed onset potential for EtOD reduction on silver in DMF (Figure S5). The presence of EtOD does induce higher hydrogenolysis rates at potentials where direct EtOD reduction does not occur. Notably, the radical trap 4-vinylanisole does not induce similar increases in the hydrogenolysis rate (Figure S22), indicating that EtOD is not accelerating hydrogenolysis via a radical pathway. This observation is also in line with a previous work which found that protic solvents accelerate the electrochemical reduction of organic halides.^22^ Because the hydrogenolysis rate increases in its presence, EtOD does not react with already formed carbanion intermediates and suggests a possible concerted proton–electron transfer (CPET). Notwithstanding this limitation, EtOD can be used to obtain important mechanistic insights about the hydrogenolysis mechanism via changes in the deuteration of products as discussed below.

Examining the incorporation of deuterium into the hydrogenolysis product **1b** and the solvent enables identification of the process occurring at the second cathodic peak. The fraction of deuterated alkane shows a statistically significant drop (*p* < 9.1 × 10^–6^) going from −2.0 to −2.1 V, while remaining fairly constant on either side of this drop (Figure 2B). The origin of the decrease in the deuterated fraction of **1b** is a more rapid increase in the formation rate of protonated **1b** compared to that for deuterated **1b** beginning around −2.1 V (Figure 2C). At the same time, a statistically significant increase in the amount of deuterium is seen only at the formyl position (*p* < 7.1 × 10^–3^) in DMF beginning at −2.1 V (Figure 2D). This observation indicates that the formyl position is deprotonated below −2.1 V. This result is in agreement with DFT calculations which predict the formyl proton is the most acidic in DMF (Table S8).

On the basis of these observations, the mechanism that is most consistent with the above data for the electrochemical process at the second cathodic peak is the reduction of R^•^_(ads)_ to **1b** by deprotonation of the solvent at voltages more cathodic than −2.1 V. If this process corresponded to reduction of a solvent radical, the deuterated fraction would have remained unchanged, which is not observed. Moreover, the reduction of R^•^_(ads)_ to **1b** would clear the surface of adsorbed species, resulting in higher current densities, consistent with observed product formation rates. Similar trends are observed when varying the initial concentration of **1a** and switching the solvent to MeCN (Figures S17 and S19), indicating this mechanism is not unique to DMF. Additional experiments confirm that the observed changes in deuterium incorporation are not a result of solvent–EtOD exchange reactions but are genuinely due to the applied potential (Figure S19).

**Figure 3:**
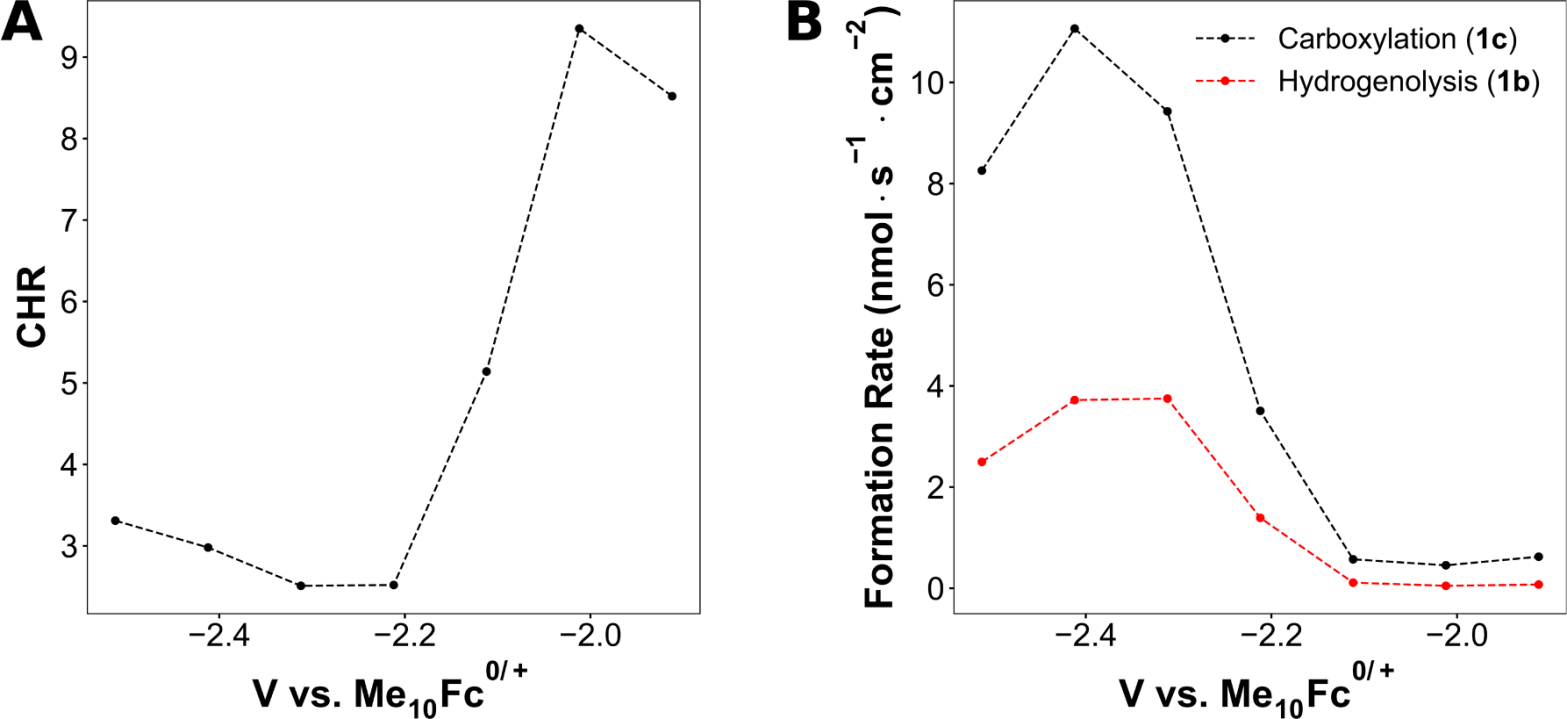
Carboxylation and hydrogenolysis of **1a** as a function of potential. (A) Carboxylation-to-hydrogenolysis ratio (CHR), calculated as the ratio of carboxylic acid (**1c**) to alkane (**1b**) formed and (B) formation rates of **1c** and **1b** as a function of potential. Reaction conditions: 20 mM **1a**, 90 mM TBA-BF_4_, 10 mM TBA-Br, DMF, 20 sccm of CO_2_. Stopping criteria: fixed passed charge or 1 h. Amount of passed charged was adjusted to obtain approximately similar conversion of **1a**; further experimental details are available in Table S4. Dashed lines are to guide the eye.

These mechanistic insights about the hydrogenolysis pathway can be leveraged to understand electrocarboxylation selectivities. The ratio of the amount of carboxylic acid (**1c**) to the amount of hydrogenolysis product (**1b**) is used as a metric for carboxylation selectivity and is denoted as the carboxylation-to-hydrogenolysis ratio (CHR). The CHR displays a strong dependence on the applied potential (Figure 3A). Similar to the results with EtOD, a sharp decrease in the CHR by about a factor of 4 occurs beginning around −2.0 V. The carboxylation rate also increases significantly after −2.1 V, although the hydrogenolysis rate increases by a proportionally larger amount, leading to lower CHRs (Figure 3B). Although the CHR is relatively high at potentials more anodic of −2.1 V, the rate of carboxylation is too low for synthetic purposes. Only at potentials more cathodic of −2.1 V can carboxylation occur at synthetically relevant rates on silver. As shown earlier by the deuterium incorporation experiments, the mechanism of hydrogenolysis at potentials more cathodic of −2.1 V involves deprotonation of the solvent. Taken together, solvent deprotonation is the primary hydrogenolysis pathway under synthetically relevant electrocarboxylation conditions.

### Solvent-Based Descriptor for Carboxylation Selectivity

The previous discussion showed that under practical carboxylation conditions in DMF the majority of the hydrogenolysis product originates from deprotonation of DMF rather than from hydrogen abstraction. On the basis of similarities between LSVs of **1a** in other solvents to its LSV in DMF (Figure S25), a reasonable assumption is that the hydrogenolysis product primarily originates from solvent deprotonation across a wide range of solvents under practical electrocarboxylation conditions. This assumption can be leveraged to develop a solvent-based descriptor that correlates strongly with carboxylation selectivity. Such a descriptor would harness mechanistic understanding to facilitate the discovery and design of improved solvents for electrochemical carboxylation of organic halides and potentially other electrochemical reductive cross-coupling reactions.

**Figure 4:**
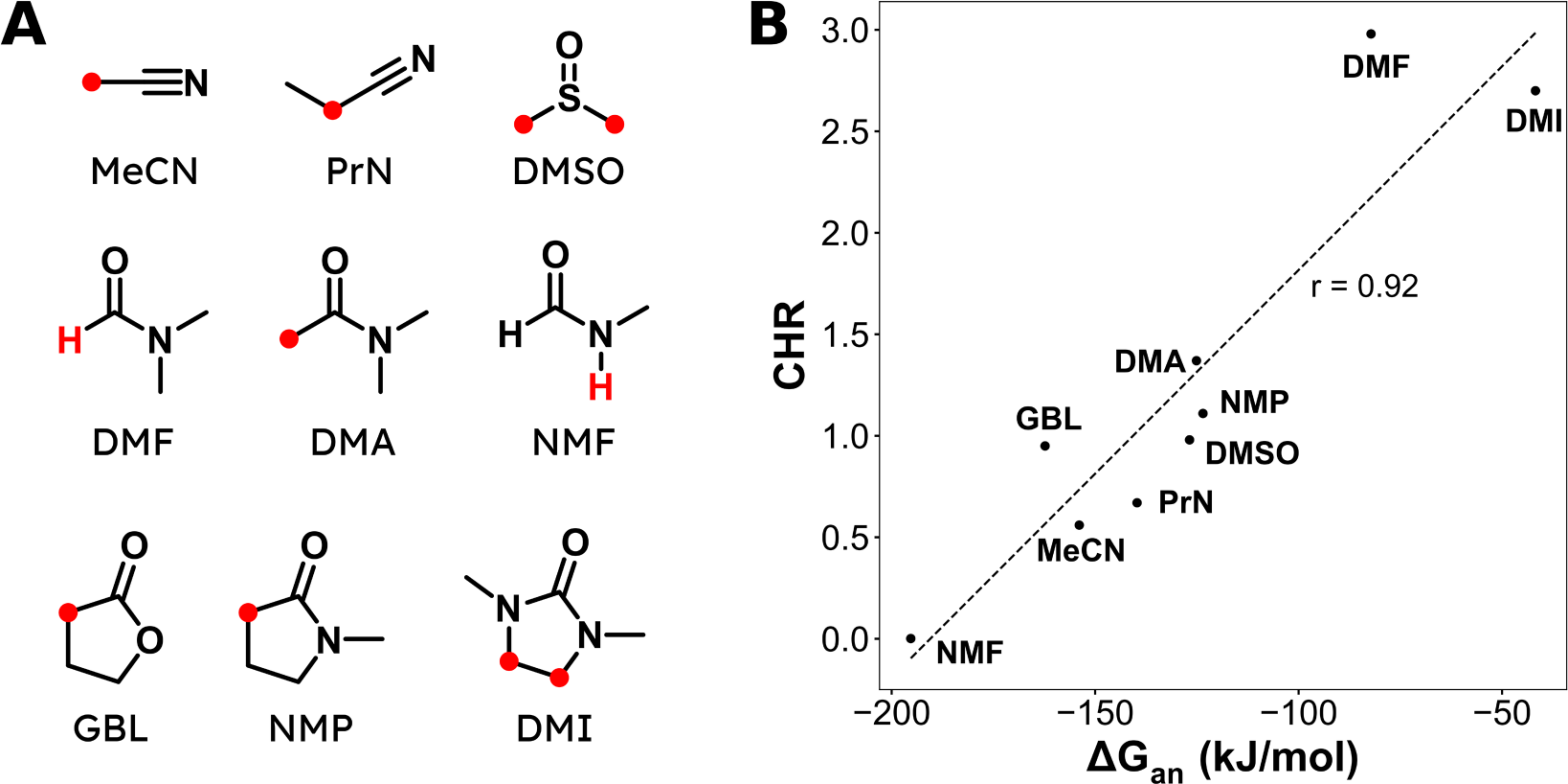
(A) Molecular structures and abbreviations for solvents. The most acidic protons are indicated in red. (B) Correlation between the deprotonation free energy (Δ*G*_an_) and the carboxylation-to-hydrogenolysis ratio (CHR). Data collected at −160 mV of the second cathodic peak of the LSV of **1a** in each solvent. Dashed line is the best linear fit to the data with a Pearson correlation coefficient of *r* = 0.92. DFT calculations performed at the M06-2X/def2-TZVPD level using PCM solvation. Experimental conditions: Ag cathode, Al anode, undivided cell, 20 mM **1a**, 90 mM TBA-BF_4_, 10–15 mM TBA-Br (increased from 10 mM as necessary to keep cell voltage within potentiostat’s limits), 2.5 mL of solvent, 20 sccm of CO_2_. Further experimental details are available in Table S4.

To ensure the robustness of any observed correlations, carboxylation was performed in a variety of solvents (Figure 4A) under several conditions, including both constant current (−5 mA/cm^2^) and constant potential electrolyses. For constant potential electrolyses, three different potentials were selected. Two potentials were based on the peak potential of the second cathodic peak from LSVs in each solvent (0 and −160 mV), and the third was a constant potential relative to Me_10_Fc^0/+^ (−2.3 V). Using potentials relative to the second cathodic peak potential ensures similar operating regimes (i.e., whether solvent deprotonation or hydrogen abstraction is predominant) across solvents, while holding potential constant relative to Me_10_Fc^0/+^ keeps the chemical potential of electrons in the cathode consistent across solvents.

For all experimental conditions, the CHR was used as the experimental data against which to assess the descriptors. Although mass transport does affect the reduction of **1a** under most of the conditions tested, the CHR is a selectivity metric and should not depend too strongly on the amount of **1a** near the electrode surface. We have examined transport limitations of CO_2_ in our system and found they should not influence the results (Figure S29). Carboxylation and hydrogenolysis products comprised the vast majority of products derived from the reduction of **1a** in all tested solvents (Tables S4–S7), which further supports only needing to examine the CHR to assess carboxylation selectivity.

Because hydrogenolysis primarily occurs via solvent deprotonation, a natural choice for a solvent-based descriptor would be the p*K*_a_ of the solvent. While solvent p*K*_a_ does display a moderate correlation with CHR (Figure S12), directly measuring the p*K*_a_ of many aprotic solvent molecules relevant for electrocarboxylation is not feasible due to their high basicities (e.g., DMF). Other commonly used solvent parameters such as Kamlet–Taft parameters and Gutmann numbers either fail to have a strong correlation with CHR or are not sensitive enough to differentiate solvents with the highest CHRs (Figure S12). Because of the limitations of experimental descriptors available in the literature, a computational descriptor based on the free energy of deprotonating a solvent molecule (Δ*G*_an_, formation energy of a solvent anion) was employed. The protonation of a carbanion (R^–^) can be used as a reference reaction, although this specific choice does not qualitatively influence the results.
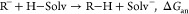
The advantage of this type of descriptor is that it can be rapidly calculated for any arbitrary solvent molecule and avoids calculating energies of solvated protons, enabling efficient screening of a wide variety of candidate solvents.

To compute Δ*G*_an_, density functional theory (DFT) calculations with continuum solvation (PCM) at the M06-2X/def2-TZVPD level of theory were used.^40−44^ Less expensive DFT methods could also be used to calculate descriptors that correlated to experimental CHRs nearly as well as those from M06-2X/def2-TZVPD + PCM (Figure S13). Because many of these solvents have more than one deprotonation site, only the most acidic (i.e., most negative Δ*G*_an_) site was used to for the descriptor. As a check, we generated a composite descriptor incorporating the deprotonation energies of all the C–H bonds in each solvent (*Q*_an_, see the Supporting Information). These computational descriptors were evaluated to determine how well they correlated to the experimentally observed CHRs from the selected solvents.

For a variety of different polar, aprotic solvents (Figure 4A), a strong correlation is observed between the calculated Δ*G*_an_ and the experimentally observed CHR under both constant current and constant potential experiments (Figures S10 and 4B). As the solvent molecule becomes harder to deprotonate (less negative Δ*G*_an_), the CHR generally increases, in agreement with the expected trend if solvent deprotonation is controlling the amount of hydrogenolysis product created. In particular, the best correlation (*r* = 0.92) is observed for the experiments conducted at −160 mV of the second cathodic peak (Figure 4B). Out of the conditions tested, this potential is the most cathodic, which helps reduce the importance of hydrogen abstraction, leaving carboxylation and solvent deprotonation as the primary pathways for the reduction of **1a**. The composite descriptor *Q*_an_ performed similarly to Δ*G*_an_ since all of the solvents examined have one C–H bond that is much more easily deprotonated than the rest (Figure S11). The lowest free energy of abstracting a hydrogen, Δ*G*_rad_, and its composite descriptor, *Q*_rad_, show no correlation with CHR, consistent with solvent deprotonation being the primary hydrogenolysis pathway at potentials relevant to practical carboxylation (Figure S11).

The easiest solvent to deprotonate within the selection, *N*-methylformamide (NMF), displayed almost negligible carboxylation activity, with hydrogenolysis predominating. Solvents more acidic than NMF would also be expected to be fairly poor for carboxylation. At the other end, the two solvents most resistant to deprotonation, DMF and 1,3-dimethyl-2-imidazolidinone (DMI), showed the highest CHRs. The remaining solvents are clustered together; these solvents share the similar characteristic of having a-CH_2_ or -CH_3_ alpha to an unsaturated electron-withdrawing group (carbonyl or nitrile). These α-CH_x_ protons are known to be acidic and represent an undesirable functional group to have in a solvent for selective electrocarboxylation.

To further support the use of the DFT-derived Δ*G*_an_, these computational values were compared to an experimental acidity metric developed in this work. This metric is based off of the exchange rate between EtO^–^ and the solvent, as measured by deuterium incorporation into the solvent during electrolysis with EtOD using ^2^H NMR. The log–linear relationship between Δ*G*_an_ and the amount of deuterium exchange confirms that Δ*G*_an_ is representative of how easily C–H bonds in aprotic solvents are deprotonated (Figure S28).

**Scheme 3:**
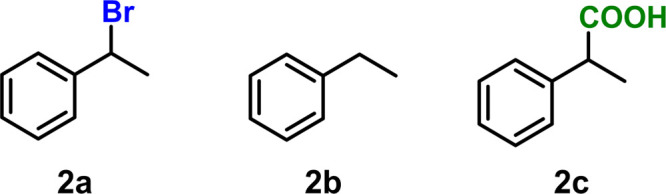
Chemical Structures of the Benzylic Halide (**2a**) and Its Corresponding Hydrogenolysis (**2b**) and Carboxylation (**2c**) Products

Benzylic halides are also common substrates in carboxylation and cross-coupling reactions. To gain a sense of how important the solvent may be for other types of organic halides, (1-bromoethyl)benzene (**2a**) was carboxylated in the same series of solvents (Scheme 3). **2a** reduces more easily than **1a** as a result of benzylic stabilization of intermediate radicals and anions (Figure S26). The CHR values after carboxylation of **2a** are above 20 in all solvents except NMF. Furthermore, no correlation could be found between the anionic or radical DFT descriptors (Figure S15). The carboxylation selectivity of **2a** is already high, so beyond NMF, solvent acidity properties no longer have a significant impact. Carboxylation of **2a** in MeCN-*d*_3_ resulted in only partial deuteration of the hydrogenolysis product, which suggests that trace water or other impurities may become relevant at these low hydrogenolysis rates. In context, CHRs for alkyl halides are fairly sensitive to solvent choice while for benzylic halides, CHRs are rather independent of the solvent beyond a certain acidity limit.

## Conclusions

The choice of solvent is a critical design parameter for electrosynthetic reactions, yet this selection is often done empirically from a limited set of solvents. In the case of electrocarboxylation of organic halides with CO_2_ at heterogeneous cathodes, the solvent can play a decisive role in the selectivity of the reaction by controlling the rate of the competing hydrogenolysis side reaction. Through mechanistic investigations, this work showed that hydrogenolysis products incorporate hydrogen atoms derived from the aprotic solvent and that under synthetically relevant conditions, hydrogenolysis occurs via solvent deprotonation rather than hydrogen abstraction. On the basis of this mechanistic understanding, a computational solvent descriptor involving the free energy to deprotonate a solvent molecule was formulated. This descriptor is readily calculable by standard DFT methods for any arbitrary solvent molecule and was found to give a strong correlation with carboxylation selectivity. A deuterium exchange rate experiment confirmed the appropriateness of the DFT descriptor to capture the ease of solvent deprotonation. Common empirical solvent descriptors were unable to correlate with experimental carboxylation selectivities as strongly as the DFT descriptor across all tested solvents. The sensitivity of carboxylation selectivity to the solvent is a function of substrate choice, with benzylic halides having carboxylation selectivities that are almost independent of the solvent choice while alkyl halides depend more strongly on the solvent choice. These results not only provide a tool to select better performing solvents for carboxylation but also illustrate how a mechanistic understanding can facilitate rational solvent selection in electrochemical reactions.

## Supporting Information Available

Detailed experimental procedures, additional product distribution data, product characterization, descriptor correlations, electrochemical characterization, and DFT optimized geometries (PDF)
