# Supplementary material for: Toward Improving the Selectivity of Organic Halide Electrocarboxylation with Mechanistically Informed Solvent Selection
Source: J Am Chem Soc. 2023 Jan 10;145(3):1740–8. doi: 10.1021/jacs.2c10561 (PMC9880992; doi:10.1021/jacs.2c10561)
Supplement: Supplementary file 1 — ja2c10561_si_001.pdf [file ja2c10561_si_001.pdf]

Supporting Information

Toward Improving the Selectivity of Organic-Halide Electrocarboxylation with Mechanistically Informed Solvent Selection

Nathan Corbin[1], Glen Junor[1], Thu N. Ton[2], Rachel J. Baker[2], and Karthish Manthiram[2]*
[1]Department of Chemical Engineering, Massachusetts Institute of Technology, 77 Massachusetts Avenue, Cambridge, Massachusetts 02139, United States
[2]Division of Chemistry and Chemical Engineering, California Institute of Technology, Pasadena, California 91125, United States
*Correspondence: karthish@caltech.edu

Contents

1 Materials ... 3
1.1 Electrolyte Salts and Solids Handling ... 5
1.2 Solvent and Substrate Purification ... 5
2 Experimental Methods ... 7
2.1 Electrolyte Preparation ... 7
2.2 Reference Electrodes ... 7
2.3 Electrochemical Cell Setup and Cleaning ... 9
2.4 Product Quantification ... 12
2.4.1 Carboxylation Experiments ... 12
2.4.2 Deuterium Incorporation Experiments ... 15
2.5 Voltammetry ... 16
2.6 Choice of Anode Material ... 17
2.7 Experiments in Propylene Carbonate ... 19
3 Solvent-Based Descriptor for Carboxylation Selectivity ... 20
3.1 Computational Methodology ... 20
3.2 Computational Descriptors ... 20
3.3 Deuterium Exchange Descriptor ... 22
4 Additional Figures and Discussion ... 23
5 Geometries of Molecules, Anions, and Radicals ... 65
5.1 DMA ... 65
5.2 DMF ... 67
5.3 DMI ... 69

5.4 DMSO 71
5.5 GBL 72
5.6 MeCN 74
5.7 NMF 75
5.8 NMP 77
5.9 PrN 80
5.10 Ethylbenzene 81
5.11 *n*-Propylbenzene 82
6 References 84

1 Materials

Name	Specifications	Vendor
Acetone	ACS, ≥ 99.5%	Fisher, Sigma-Aldrich
Diethyl ether (Et_2O)	ACS, anhydrous, BHT stabilized, >99%	J.T. Baker, Fisher
Hexane	ACS, > 98.5% isomers	VWR-BDH
Cyclopentane (CyP)	99%	Beantown Chemical
Dichloromethane (DCM)	ACS, 99.5%, stabilized	Fisher
N,N-Dimethylformamide (DMF)	anhydrous, 99.8%	Sigma-Aldrich
Acetonitrile (MeCN)	anhydrous, 99.8%	Sigma-Aldrich
Dimethylsulfoxide (DMSO)	anhydrous, 99.9%	Sigma-Aldrich
Propylene carbonate (PC)	anhydrous, 99.7%	Sigma-Aldrich
N-Methylformamide (NMF)	99%	Sigma-Aldrich
N,N-Dimethylacetamide (DMAc)	anhydrous, 99.8%	Sigma-Aldrich
Tetrahydrofuran (THF)	≥ 99.0%, 25 ppm BHT, ACS	Sigma-Aldrich
Dimethoxyethane (DME)	≥ 99%, reagent grade	Sigma-Aldrich
Propionitrile (PrN)	99%	Alfa Aesar
N-Methylpyrriolidinone (NMP)	anhydrous, 99.5%	Sigma-Aldrich
Gamma-butyrolactone (GBL)	reagent plus, 99%	Sigma-Aldrich
1,3-Dimethyl-2-imidazolidinone (DMI)	absolute over sieves, ≥ 99.5%	Sigma-Aldrich
Tetraglyme	99%	Alfa Aesar
Chloroform-d ($CDCl_3$)	99.8 (0.03% TMS)<br>99.8%, .05% v/v TMS	Sigma-Aldrich<br>Cambridge Isotope Laboratories
Acetonitrile-d_3 (MeCN-d_3)	≥ 99.8%	Sigma-Aldrich
Dimethylsulfoxide-d_6 (DMSO-d_6)	99.9%	Sigma-Aldrich
Ethanol-OD (EtOD)	99%	Sigma-Aldrich
Deuterium oxide (D_2O)	99.9%	Cambridge Isotope Laboratories
Water (MilliQ)	18.2 Ohm	EMD Millipore
Platinum (Pt)	99.99% trace metal, 0.025 mm thick	Beantown Chemical
Magnesium (Mg)	magnesium alloy	Amazon (More Metals)
Silver (Ag)	99.998% metals basis, 0.1 mm thick, hard, Premion	Alfa Aesar
Aluminum wire	99.999%, 2.0 mm diameter, annealed	Alfa Aesar
Aluminum foil (Al)	99.45%, 0.025 mm thick	Alfa Aesar
	99.9999%, 0.25 mm thick, annealed	Alfa Aesar
	99.9995%, 0.25 mm thick, annealed	Alfa Aesar
Carbon dioxide (CO_2)	99.999%	Airgas
Nitrogen (N_2)	Liquid N_2 boil-off	Airgas
Nitric acid (HNO_3)	Trace metal grade, 67-70 wt.%	Fisher
Hydrobromic acid (HBr)	ACS, 47-49 wt.%	Beantown Chemical
Hydrochloric acid (HCl)	ACS, 37 wt.%	Sigma-Aldrich

1,3,5-Trimethoxybenzene (TMB)	≥ 99%	Sigma-Aldrich
1-Bromo-3-phenylpropane (**1a**)	98%	Sigma-Aldrich
(1-Bromoethyl)benzene (**2a**)	97%	Alfa Aesar
n-Propylbenzene (**1b**)	analytical standard	Sigma-Aldrich
Ethylbenzene (**2b**)	anhydrous 99%	Sigma-Aldrich
4-Phenylbutyric acid (**1c**)	99%	Beantown Chemical
2-Phenylpropanoic acid (**2c**)	97%	Sigma-Aldrich
Tetra-n-butylammonium tetrafluoroborate (TBA-BF_4)	> 98%	TCI
Tetra-n-butylammonium bromide (TBA-Br)	≥ 99.0%	Sigma-Aldrich
Tetra-n-butylammonium iodide (TBA-I)	98%	Sigma-Aldrich
Aluminum oxide (Al_2O_3)	powder, 99.995% ultra-pure	Inframat Advanced Materials
Magnesium oxide (MgO)	99.995% trace metals basis	Beantown Chemical
Lithium tetrafluoroborate ($LiBF_4$)	98%	Sigma-Aldrich
Magnesium bromide ($MgBr_2$)	98%, anhydrous	Acros Organics
Aluminum bromide ($AlBr_3$)	anhydrous 98+%	Acros Organics
Decamethylferrocene ($Me_{10}Fc$)	99%	Beantown Chemical
Ferrocene (Fc)	99%	Alfa Aesar
Iodine (I_2)	ACS, ≥ 99.8%	Sigma-Aldrich
Molecular sieves	4-8 mesh	Acros Organics
Sodium carbonate (Na_2CO_3)	BioXtra ≥ 99.0%	Sigma-Aldrich
Sodium tetrafluoroborate ($NaBF_4$)	98%	Sigma-Aldrich
Phosphorus pentoxide (P_2O_5)	reagent plus, 99%	Sigma-Aldrich
Calcium hydride (CaH_2)	reagent grade, 95%	Sigma-Aldrich
Magnesium sulfate ($MgSO_4$)	Reagent grade	VWR
Tetraethylammonium tetrafluoroborate (TEA-BF_4)	99%	Sigma-Aldrich
Tetraethylammonium bromide (TEA-Br)	99+%	Acros Organics

1.1 Electrolyte Salts and Solids Handling

All electrolyte salts were vacuum dried overnight (12+ hr) and stored in an argon-filled glovebox (Vacuum Atmospheres Genesis) with H_2O levels at or below 11 ppb. TBA-BF_4, TBA-Br, TEA-BF_4, and TEA-Br were dried at 60 – 80 °C; TBA-I was dried at 50 °C (we did observe some oxidation of the iodide if the vacuum chamber was not purged several times with N_2); $LiBF_4$, MgO, and Al_2O_3 were dried at 90 °C. Due to their very hygroscopic nature, $MgBr_2$ and $AlBr_3$ were taken directly into the glovebox after receiving them; $AlBr_3$ was stored in a refrigerator. Molecular sieves were reused by washing twice with acetone, drying at 350 °C for at least 4 hours, cooling under vacuum, and backfilling with N_2. They were kept under a nitrogen blanket to minimize residual oxygen in the pores.

1.2 Solvent and Substrate Purification

To help minimize the impact of impurities across solvents with varying degrees of commercial purity, all electrolyte solvents were subject to distillation. After distillation, solvents were stored over 3 Å molecular sieves (approximately 15–20 vol.%) and kept under a N_2 blanket in amber glass vials or jars. For solvents which could not be readily purchased anhydrous with an air-tight septum, these were dried over 3 Å sieves before distillation as well to help minimize water content. For solvents with a boiling point above 150 °C, vacuum distillation was used with a pump (Welch 1400 DuoSeal) that could reach base pressures as low as 10 mtorr. Collection was generally stopped when the boiling flask was low (~ < 1 mL). For distillations at ambient pressure, the first ~ 200–500 μL of distillate was discarded. For vacuum distillations, the system was held for at least 30 min under pressure before distillation to remove volatiles. The collection flask was set in an ice bath when collecting distillate for solvents with freezing points below 0 °C. For heating, we used a water bath (T < 90 °C) or oil bath (T > 90 °C) kept at a specified temperature to avoid overheating the boiling flask. We avoided using calcium hydride because although it has been recommended to purify some of the solvents,[1] we noticed that some reduction of the solvent occurred. Specific purification steps and conditions for each solvent and substrate are given below. Deuterated solvents were distilled under the same conditions as their protonated counterparts.

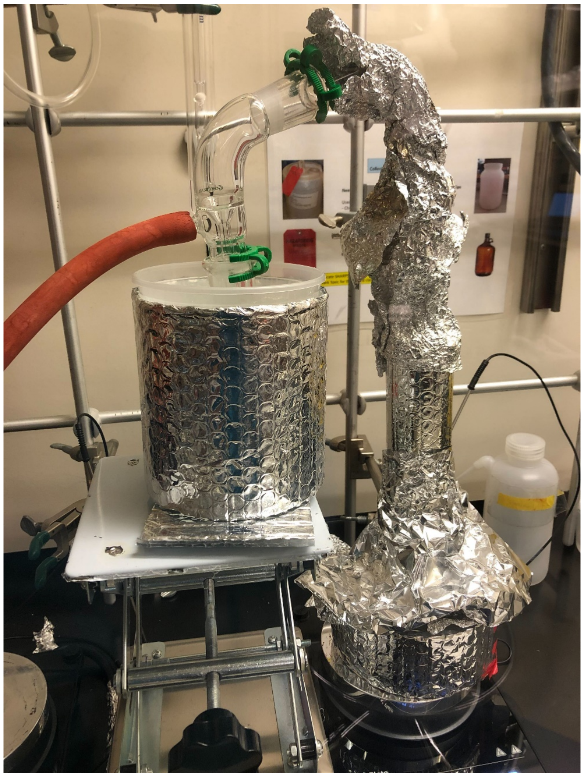

Figure S1. Vacuum distillation setup with insulation.

Acetonitrile (MeCN): Ambient pressure distillation. Water bath set initially to 120 °C, and temperature increased a bit as necessary to drive distillation to completion. Ice bath for distillate collection.

Propionitrile (PrN): Stored over 3 Å sieves before distillation. Ambient pressure distillation. Water bath set initially to 140 °C. Ice bath for distillate collection.

N,N-Dimethylformamide (DMF): Vacuum distillation. Initially pulled vacuum with boiling flask in an ice bath to reduce DMF evaporation. Increased water bath temperature to 25–30 °C to begin distillation. Ice bath for distillate collection.

N,N-Dimethylacetamide (DMAc): Vacuum distillation. Increased water bath temperature to 40 °C to begin distillation. Ice bath for distillate collection.

N-Methylformamide (NMF): Stored over 3 Å sieves before distillation. Vacuum distillation. Increased water bath temperature to 40 °C to begin distillation. Ice bath for distillate collection.

N-Methyl-2-pyrrolidone (NMP): Vacuum distillation. Increased water bath temperature to 40–45 °C to begin distillation. Ice bath for distillate collection.

Dimethylsulfoxide (DMSO): Vacuum distillation. Increased water bath temperature to 40–45 °C to begin distillation. Collection flask kept at room temperature.

Gamma-butyrolactone (GBL): Vacuum distillation. Increased water bath temperature to 40–45 °C to begin distillation. Ice bath for distillate collection.

1,3-Dimethyl-2-imidazolidinone (DMI): Vacuum distillation. Increased water bath temperature to 60 °C to begin distillation. Ice bath for distillate collection.

Propylene Carbonate (PC): Vacuum distillation. Increased water bath temperature to 45 °C to begin distillation. Ice bath for distillate collection.

1-Bromo-3-phenylpropane (**1a**): Vacuum distillation over P_2O_5 (~ 100 mg for 5 g of **1a**). Increased water bath temperature to 40 °C to begin distillation. Ice bath for distillate collection. Kept as much of the apparatus covered by aluminum foil as possible to prevent degradation from light. Stored in original amber glass jar under a nitrogen blanket. Distillation over P_2O_5 removed alcohol impurities, which could frequently overlap with the carboxylic acid peak during GC-MS. Impurity characterization by GC-MS before and after distillation found the distillation improves the purity, especially removing compounds with longer retention times than **1a**. The purity of **1a** was estimated to be > 99% after distillation based on FID area ratios.

2 Experimental Methods

2.1 Electrolyte Preparation

Since the reduction of organic halides can be sensitive to the presence of protic impurities such as water, our handling procedures attempted to avoid exposure to water as much as possible while running electrochemical experiments outside a glovebox (Vacuum Atmospheres Genesis). All electrolyte salts were stored and weighed out in an argon-purged glovebox with H_2O levels at or below 11 ppb. Once weighed out, salts were added to 20 mL glass vials, capped, and transferred to a nitrogen-filled glovebag (Aldrich AtmosBag, size S, zipper lock) for solvent addition. For desiccant, the glovebag had a container of 3 Å sieves, and before each usage, a bit of P_2O_5 was spread out in a glass dish to further reduce moisture levels. Solvents were then added to the vials containing electrolyte salts and shaken to dissolve the solids. For experiments involving product quantification after electrolysis of **1a**, an amount of **1a** and solvent were weighed precisely the day before and kept over sieves (~ 2 sieves per 2.5 mL solution) and under a nitrogen blanket in an amber glass vial; this procedure was found to help improve carboxylation selectivities. For all solvents, GC-MS was used to confirm that these solutions did not degrade **1a** after overnight storage. We did find that sometimes during storage in DMSO over sieves, 2–5% of **1a** converted into 3-phenylpropionaldehyde (~ 2–5%) via Kornblum oxidation. However, we found that carboxylation selectivities were higher compared to adding **1a** directly to DMSO right before experimentation in the glove bag, so we deemed the small amount of oxidation not impactful. Other substrates and additives were added directly to the cell after the bulk electrolyte had been added.

2.2 Reference Electrodes

For experiments involving a reference electrode, a Pt wire (CH Instruments, CH112 with Pt wire) was immersed in a roughly 10 mM solution of I_3^-/I^- in the solvent used for electrolysis. The nominal compositions of the reference electrode solutions were 10 mM I_2, 20 mM TBA-I, and 80 mM TBA-BF_4. The reference electrode solution was contained in a glass tube with a porous Teflon tip (4 mm O.D., CH Instruments). The reference electrode was inserted into a PTFE hex-head

screw (3/8”-16 thread size, 1” long, McMaster Carr) with a 4 mm hole drilled through it. The hex-head screw + reference electrode assembly was carefully screwed into the cell to avoid overtightening which could snap the glass tube. While there was some variability in how snugly the reference electrode fit through the screw, tightening the screw in the cell also tightened the connection between the screw and reference, making a gas-tight seal.

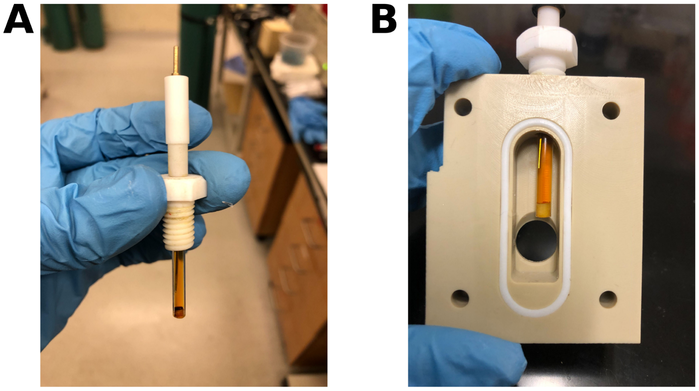

Figure S2. (**A**) Reference electrode assembly showing the reference electrode with I^-/I_3^- filling solution fit through a PTFE hex-head screw. (**B**) Reference electrode assembly screwed into the cell to a depth just above the working electrode opening.

The use of a $I_3^-/I^-/Pt$ reference electrode was motivated by (1) its ease of construction in a variety of solvents, (2) fast reaction kinetics, (3) stability, and (4) low potential impact upon leakage.[2–4] All reference electrodes leak to some extent, with low leakage rates around a few µL/hr. At this rate, the amount of triiodide and iodide introduced into the bulk electrolyte in a typical experiment would be on the order of 10 µM (2.5 mL electrolyte volume). In terms of stability, we did observe that reference electrode solutions made some solvents (e.g. PrN) would vary by 20–30 mV per day initially. Letting these references sit for a few days was found to help improve stability, possibly by allowing all of the I_2 to convert to I_3^- (the potential vs. $Me_{10}Fc$ would gradually become more positive). Once stabilized, the reference potential drift would become much smaller to at most a few mV per day.

Reference electrodes were calibrated before and usually after all of the experiments in a given day by referencing to the $Me_{10}Fc^{0/+}$ couple, as the standard potential of this couple has been shown to be more stable than that of $Fc^{0/+}$ across a wide variety of solvents.[5,6] Calibration was performed by conducting at least five consecutive cyclic voltammograms (CVs) of a solution of $Me_{10}Fc$ (100 mM TBA-BF_4 in desired solvent) at 50 mVs with 85% IR compensation at a Pt electrode. The average of the anodic and cathodic peak potentials was used to calculate the potential of the reference electrode vs. the $Me_{10}Fc^{0/+}$ couple; the final reference potential was calculated as the average of the reference potentials of the last four CV scans. The concentration of $Me_{10}Fc$ was not controlled; it typically was sparingly soluble in most solvents, so 10–20 mg was added and allowed to dissolve. In all cases, enough would dissolve to produce well-defined voltammetric peaks.

2.3 Electrochemical Cell Setup and Cleaning

Silver Electrodes

Silver foils (25 mm x 25 mm) were polished for ~ 1.5 min using 400 G silicon carbide sandpaper (Norton, Blue-Bak T414) on a piece of aluminum foil on a cut piece of a polystyrene weigh boat trimmed to make a shallow dish (VWR, 85 x 85 x 24 mm, anti-static). MilliQ water was added to fully submerge the silver foils on the aluminum. The polishing time was apportioned as follows: 1 min with small circular polishes using one half of the sandpaper and 30 sec of unidirectional polishing to produce a visibly uniform finish using the other fresh half of the sandpaper. A fresh piece of sandpaper was used for each new electrode, and a new piece of aluminum was used after 3–4 polishes. A second layer of nitrile gloves was worn only during polishing to minimize possible silver contamination. After polishing, the electrodes were rinsed with acetone (10–15 seconds per rinse, both sides of the foil). Then, a section of a Kimwipe was wetted with acetone, and the active side of the foil was wiped with the wetted Kimwipe to remove remaining solid particulates. The foils were then submerged in 1 M HNO_3 for 2–3 min seconds followed by rinsing with MilliQ water (10–15 sec, both sides of the foil). The foils were sonicated (VWR Symphony, 90 W, 35 kHz) for at least 2 min in MilliQ water followed by rinsing with acetone (10–15 sec) and then blown dry with house nitrogen. The foils were stored in a covered, vented glass dish in a drying oven (Binder FD forced convection oven) at 80 °C until they were ready to be used (at least 15 min). After electrolysis experiments, Ag electrodes were washed briefly with acetone before storage.

Aluminum Electrodes

Aluminum foils were cleaned by submerging in 1 M HNO_3 for 2–3 min, followed by rinsing with acetone and wiping with an acetone-wetted Kimwipe. They were stored in a drying oven at 80 °C until needed. After electrolysis experiments, Al electrodes were washed briefly with acetone before storage.

Platinum Electrodes

Platinum electrodes were rinsed with acetone and dried at 80 °C for use as counter electrodes or electrodes during reference electrode calibration.

Magnesium Electrodes

Fresh magnesium anodes were dried at 80 °C before use. Each side of the magnesium anode was used once; further uses would often result in leaking likely due to the O-rings not making a perfect fit against the roughened surface of a used magnesium surface.

Electrochemical Cell Components

After each experiment, the PEEK cells (including the PTFE O-rings) were washed with acetone. Occasionally, insoluble particulates remained in the cell, which could be removed by wetting a Kimwipe with acetone and scrubbing the particulates off. The cells were dried at 80 °C before being submerged into 20 wt.% HNO_3. The cells were sonicated for at least 5 min in 20 wt.% nitric acid. The cells were then removed from the nitric acid, washed with copious amounts of

MilliQ, and sonicated for at least 3 min in MilliQ. The cells were then washed with acetone and dried at 80 °C overnight (ideally). TEFZEL (ETFE) plugs were allowed to soak in DCM overnight and then dried at 80 °C.

After extended use with vacuum grease, the PEEK cells, and TEFZEL nuts and plugs were soaked in hexane at 60 °C for several hours to the grease. They were washed with acetone and dried at 80 °C for several hours.

Reference electrodes were cleaned by washing extensively with acetone to remove all color from iodine. A blunt-tipped syringe was used to clean the inside of the glass body. To remove residue in the Teflon tip, the glass body was gently held on a paper towel to drain liquid through the tip. All reference electrode components were dried at 80 °C.

Electrochemical Cell Assembly

A custom PEEK electrochemical cell was used for all electrochemical experiments. The design specifications for these cell have been described in our previous work (SI Section 3.5 from Ref. 7). Sealing was achieved with PTFE O-rings (Rigid, Durometer 55D, Dash 015 and 027, McMaster-Carr). The current collectors were made of commercial aluminum foil taped onto PEEK backing plates. Cells were assembled with the main cell compartment and electrodes taken freshly out of the oven. Care was taken to ensure O-rings made full contact with electrodes. A PEEK spacer was used to achieve a proper height to not exceed the threading on the screws. Cells were hand-tightened with wingnuts; this was sufficient to achieve gas and liquid-tight seals. The cell assembly procedure is pictorially shown below for a one-compartment cell.

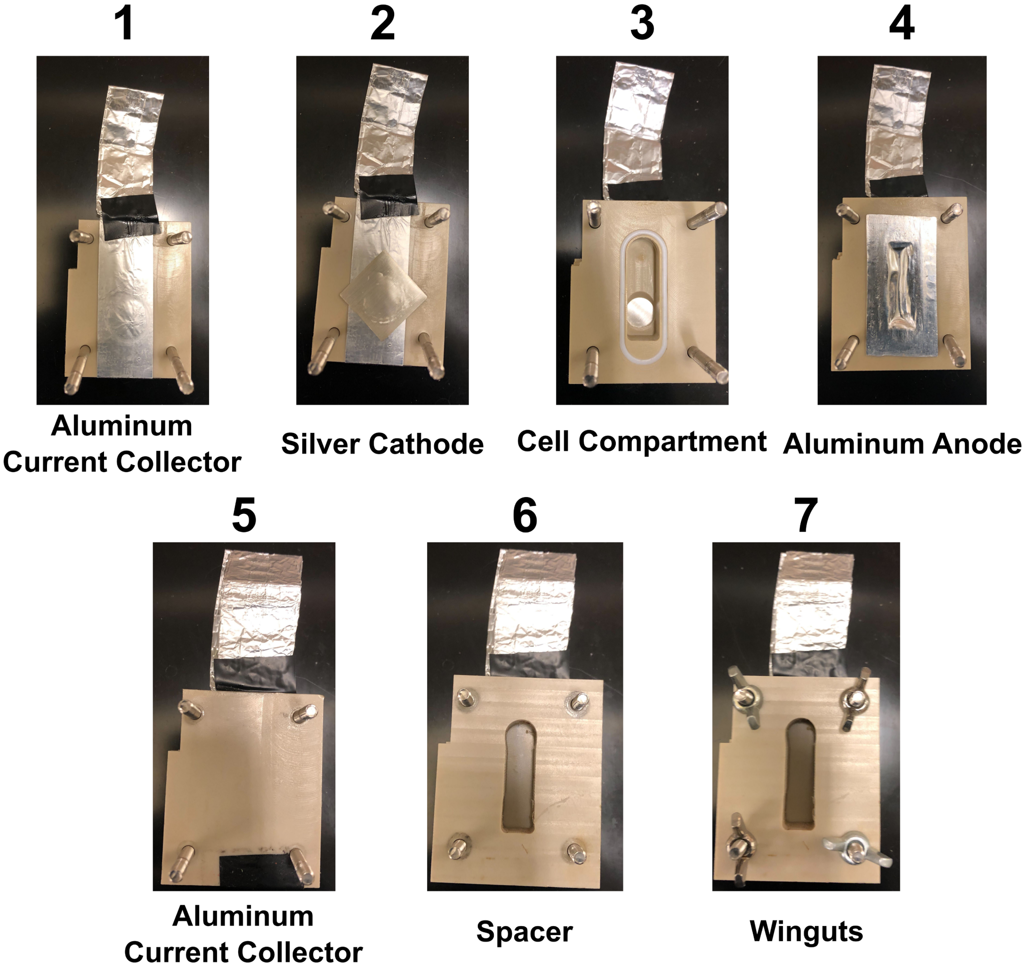

Figure S3. Steps for assembling the one-compartment cell used in this study.

After the cell was assembled as in **Figure S3**, it was connected to flowing gas (either CO_2 or N_2) via 1/16" O.D. FEP tubing through the diagonal side port (left notch in **Figure S3**). A piece of 1/8" O.D. FEP tubing was used as an outlet; this tubing was connected to an in-line gas chromatograph when measurements of CO and H_2 were desired. The remainder of the top ports were plugged. After flowing gas through the empty cell for at least 2 min, a flow meter was connected to the outlet to confirm the cell was gas tight. In instances were gas tightness was not initially achieved, a small amount of vacuum grease was used, typically being applied to either the threads of the nut of the inlet tubing or the reference electrode. After gas tightness was confirmed, the appropriate volume of electrolyte and other reagents were added. Then, the reference electrode assembly was inserted and tightened. We note that overtightening the reference electrode assembly can cause the glass tubing to shatter. At this stage, the electrolyte was allowed to become saturated with gas for at least 10 min before any electrochemical experiments were conducted.

2.4 Product Quantification

2.4.1 Carboxylation Experiments

For most experiments, two general workup procedures were used depending on whether the solvent preferred to partition in water or in Et_2O. To avoid plastic contamination, glass or metal was used wherever possible when handling extraction solvents. All volumes are estimated based off the volume of a full bulb squeeze of liquid in a Pasteur pipette (1.75 mL). The general objective of most extractions was to separate the organic halide substrate and all of its products while removing most of the solvent and nearly all of the electrolyte salt. We observed that tetra-n-butylammonium (TBA) salts can impact the quantification of carboxylic acid by GC-MS-FID, likely by decomposing into tri-n-butylamine which can react with acids to form non-volatile products. All extraction procedures were designed to get nearly undetectable amounts of TBA salts in the final product mixture.

Carboxylation workup with the solvent preferring to partition into water

Solvents: DMF, DMAc, NMF, NMP, DMI, GBL, DMSO

After the completion of electrolysis, the electrolyte was removed from the cell with a long glass Pasteur pipette and added to a glass centrifuge tube (Pyrex 15 mL conical bottom). The cell was washed with 3.5 mL 0.2 M HBr and combined with the electrolyte. Care should be taken when (1) adding acid to the cell if an Mg anode is present and (2) adding acid to the electrolyte because significant bubbling can occur from release of H_2 and CO_2, respectively. The cell was then washed with 3.5 mL of Et_2O and combined with the electrolyte. The combined mixture was shaken, and the organic layer extracted (phase separation was improved by centrifuging at 1,000 rpm for 10 sec). The cell was washed a second time with 3.5 mL Et_2O, combined with the electrolyte, and had the organic layer extracted. Three more Et_2O extractions (1.75 mL) were performed on the electrolyte, and all organic layers were combined. The combined organic layers were washed with 3.5 mL MilliQ water. For GBL, two additional 3.5 MilliQ water washes could be performed to reduce the amount of GBL in the final solution. A weighed amount of 1,3,5-trimethoxybenzene (TMB) was added to the organic layer as an internal standard. At this stage, a sample of the organic layer was analyzed by gas chromatography mass spectrometry (GC-MS) with a flame ionization detector (FID) to quantify products.

In some cases, ^{1}H NMR quantification was used to supplement GC-MS-FID. The organic layer from the previous step was dried with $MgSO_4$, filtered, and rotavapped at room temperature to remove most of the Et_2O. To the residue was added $CDCl_3$, and ^{1}H NMR was conducted on this solution. We observed that rotary evaporation invariably caused noticeable loss of hydrocarbon products (n-propylbenzene and ethylbenzene), so GC-MS-FID was always necessary to quantify these.

Carboxylation workup with the solvent preferring to partition into Et_2O

Solvents: MeCN, PrN

After completion of electrolysis, the electrolyte was removed from the cell with a long glass Pasteur pipette and added to a glass centrifuge tube. The cell was washed with 3.5 mL 0.1 M

Na_2CO_3 and combined with the electrolyte. The cell was then washed with 3.5 mL hexane and combined with the electrolyte. The mixture was shaken, and the organic layer extracted (phase separation was improved by centrifuging at 1,000 rpm for 10 sec). The cell was washed a second time with 3.5 mL hexane, combined with the electrolyte, and had the organic layer extracted. One more hexane extraction (1.75 mL) were performed on the electrolyte. In the case of PrN, sufficient PrN moved into the organic layer such that some TBA salts also partitioned with it. To help remove these TBA salts, the organic layer was washed once with slightly acidified 1.75 mL MilliQ/175 μL DMSO, and this aqueous layer was added back to the electrolyte. This step was done to convert any carboxylate in the organic layer into acid, liberating it from TBA. The addition of DMSO helps improve the partitioning of TBA-BF_4 into the aqueous layer. A second wash with 1.75 mL MilliQ/175 μL DMSO was performed again on the organic layers and discarded.

After extracting with hexane, the aqueous electrolyte layer was extracted 2x with 3.5 mL Et_2O. These combined Et_2O layers were rotavapped at room temperature first to remove Et_2O, then at elevated temperature to remove the electrolyte solvent. The rotavapped residue was added back to the aqueous electrolyte mixture. This mixture was then acidified with a minimal amount of 4 M HCl (until bubbling from CO_2 release stopped), and 350 μL of DMSO was added. Three Et_2O extractions were performed on this acidified aqueous layer (2x 3.5 mL, 1x 1.75 mL). The combined Et_2O layers were washed with 3.5 mL MilliQ water, rotavapped down, and combined with the combined organic layers from hexane extraction. A weighed amount of TMB was added to the combined organic layers. Product quantification was achieved by GC-MS-FID, with ^{1}H NMR used occasionally to provide additional support of the GC-MS-FID results.

<u>Safety note</u>: Hexane has a high toxicity and should always be used with proper ventilation. Safer alternatives such as pentane and heptane can be used in its place for these workup procedures and any cleaning procedures.

GC-MS-FID

GC-MS-FID was the primary quantification method for products from all experiments because it was able to accurately measure both the hydrocarbon and carboxylic acid products. GC-MS-FID was conducted on a DB-Wax column on a 7890B Agilent GC-MS. The FID signal was used for quantification, while the MS signal was helpful for identifying which parts of the FID signal belonged to specific compounds. Calibration curves were constructed using TMB as an internal standard:

$$\frac{N_i}{N_{TMB}} = m_i \left(\frac{A_i}{A_{TMB}}\right) + b_i$$

where *N* represents the molar quantity of a compound, *A* represents the FID area, and *m* and *b* are the fitted calibration parameters. Subscripts indicate the specific compound. Normalizing to the amount of TMB eliminates errors from evaporation of workup solvents such as Et_2O and hexane. Calibration parameters were obtained from fits to known calibration standards made in hexane. These parameters were obtained by minimizing the squared relative (i.e. percent) error to avoid large relative errors at low concentrations, which can occur when minimizing the squared error:

$$\min_{m,b} \sum_{j=1}^{N} \left(\frac{N_{fit,j} - N_{data,j}}{N_{data,j}} \right)^2$$

Calibration curves generated in this manner generally resulted in percent errors not exceeding 4.5% in largest magnitude across two orders of magnitude of concentrations. The hydrocarbon, substrate, and carboxylic acid were explicitly calibration. Quantification of other compounds was achieved by approximating the FID area as proportional to the number of carbons bonded to at least one hydrogen.

We observed that accurately quantifying carboxylic acids required strict cleanliness protocols for the GC-MS. Specifically, avoiding TBA salts and other solids is crucial for quantifying carboxylic acids below 1 mM in concentration. Having dedicated cleaning and blank vials of DCM also reduced carryover from other experiments. The quality of the instrument was checked frequently with a low concentration calibration sample (~ 250 μM acid); once this area fell by more than 5% of its accurate value, the inlet (and possibly septa) were replaced. The carboxylic acid areas generally had long tails which needed to be accurately integrated. In cases where the acid peak was below what could be accurately quantified by the calibration curve, the entire sample was rotavapped down to increase its area. For (1-bromoethyl)benzene, a significant amount of elimination occurred on the column, resulting in the formation of styrene and a broad organic halide peak. Due to this complication, (1-bromoethyl)benzene was not explicitly quantified.

NMR

^{1}H (500 MHz) and ^{2}H (76.8 MHz) NMR spectra were collected on a Bruker Avance Neo spectrometer. Quantitative ^{1}H NMR spectra were typically acquired with 16 scans with an 8 sec acquisition time followed by a 9 sec interscan delay. Quantitative ^{2}H NMR spectra were acquired with 16 scans with a 6.8 sec acquisition followed by a 21 sec interscan delay.

In-Line Gas Chromatography

An 8610C SRI MultiGas 5 gas chromatograph with in-line sampling capabilities was used to quantify carbon monoxide (CO) and hydrogen (H_2). House N_2 was used as the carrier gas at a flow rate of ~ 40 mL/min (13 psig setpoint). The sampled gas was injected via a 1 mL sample loop and passed through a six-foot Haysep D column held at a constant temperature of 85 °C. H_2 was quantified via a thermal conductivity detector (TCD), while CO was quantified with a flame ionization detector (FID) with a pre-methanizer. A stop-flow valve was used to prevent CO_2 and organic solvents from contacting the methanizer. We did observe that some solvents such as DMF could significantly poison the methanizer, decreasing the sensitivity toward CO.

Calibration curves for CO and H_2 were constructed by dilution of an appropriate calibration gas mixture of CO and H_2 in CO_2 (Airgas) with CO_2. Two repeated, consecutive measurements were performed at each dilution to ensure repeatability. The data were divided into groups by order of magnitude, and calibration parameters were fit by minimizing the squared relative error as

detailed above. To convert measured gas concentrations (in ppm) to partial current densities, the following equation was used:

$$j_i = \frac{nFy_i\dot{N}_{tot}}{A_{elec}}$$

where j_i is the partial current density of species i, n is the number of electrons transferred to form species i (n = 2 for CO and H_2), F is Faraday's constant (96,485 C/mol), y_i is the mole fraction (obtained from the measured ppm) for species i, $\dot{N}_{tot}$ is the total molar flow rate of gas set by the mass flow controller, and A_{elec} is the geometric surface area of the cathode (always 1 cm^2). Faradaic efficiencies (FE) were calculated by taking the ratio of the partial current density to the total current density:

$$FE_i = \frac{j_i}{J_{tot}} \times 100\%$$

where FE_i is the Faradaic efficiency of species i, and J_{tot} is the total current density. Further details can be found in our previous work (SI Section 5.5).[7]

Column Chromatography

Column chromatography was used to help isolate and identify products during the electrolysis of **1a** with 4-vinylanisole (4VA). After the nonpolar compounds were isolated and concentrated by the extraction procedures described previously, they were loaded onto a silica column (Biotage Sfär Silica 60µm 5g). The products were eluted with a Biotage Isolera using hexane as an eluent. Analysis of the various fractions was performed by GC-MS, ^{1}H NMR, and ^{13}C NMR to confirm the identity of the coupling product between **1a** and 4VA.

2.4.2 Deuterium Incorporation Experiments

Two general types of deuterium incorporation experiments were performed. One involved the addition of a deuterated additive (EtOD or D_2O) to the electrolyte and running electrolysis without CO_2. The workup steps were similar to those described above for carboxylation experiments. MilliQ water replaced the HBr solution for washing the cell and forming the aqueous layer in experiments without CO_2 bubbling since there was no carboxylic acid. Additionally, only three extractions (2x 3.5 mL cell wash, 1x 1.75 mL) of the combined aqueous electrolyte with an organic solvent (hexane or cyclopentane for MeCN, Et_2O for DMF) were performed due to the high partitioning of the substrate and the alkane into the organic layer. These combined organic layers were washed with 3.5 mL MilliQ water before analysis by GCMS and ^{1}H NMR.

Quantification of the isotopic abundance in the hydrogenolysis product (*n*-propylbenzene) was best performed via GC-MS. A calibration curve for the ratio of deuterium to hydrogen was constructed based off of the relative areas of the *m/z* 120 and 121 peaks in the MS spectrum. Each of the protonated and deuterated products contributes to both of the *m/z* signals at 120 and 121, so the calibration function was constructed accordingly:

$$A_{120} = B_{120,H}N_H + B_{120,D}N_D$$

$$A_{121} = B_{121,H}N_H + B_{121,D}N_D$$

where A_i is the integrated area of the ion signal at $m/z = i$, N_j is the amount of deuterated or protonated product, and the $B_{i,j}$ are proportionality coefficients. Since the FID signal can be used to quantify the total amount of product, i.e. $N_H + N_D$, the MS signal was used to evaluate the fraction of deuterated to protonated product. Based off the calibration functions, the function describing the deuterated fraction of product is

$$f_D = \frac{k_1 + R}{k_2 + k_3 R}$$

where f_D is the fraction of deuterated product, R is the ratio of the signal at $m/z = 121$ to the signal at $m/z = 120$, and k_i are constants to be fit.

To prepare the samples for the calibration curve, a sample of ~100% deuterated *n*-propylbenzene was prepared by electrolysis in MeCN-d_3 with 1 M D_2O at constant current with a silver cathode and aluminum anode. This sample was then mixed at different ratios with commercially available ^{1}H *n*-propylbenzene. ^{1}H NMR was used to quantify the ratio of deuterated to protonated product in the calibration samples; the signals from *n*-propylbenzene were large enough to neglect errors from residual hydrocarbon contamination. The calibration curve was accurate to within about 5% (absolute error) of the nominal deuterated percentage.

^{1}H NMR was avoided as a reliable quantification technique for the deuterated fraction of *n*-propylbenzene from electrolysis experiments. The absolute error in the deuterated fraction obtained from ^{1}H NMR by the ratio of the peak area at ~0.95 ppm to the area at ~2.60 ppm scales with the percent errors on these two areas

$$\epsilon_D = -2\left(\frac{A_{0.95}}{A_{2.60}}\right)\frac{\varepsilon_{0.95} - \varepsilon_{2.60}}{1 + \varepsilon_{2.60}} \sim 2\left(\frac{A_{0.95}}{A_{2.60}}\right)(\varepsilon_{2.60} - \varepsilon_{0.95})$$

Plastic contamination from general laboratory equipment and traces of vacuum grease from sealing the cell will increase the peak area at 0.95 ppm, with the percent error becoming larger at small amounts of *n*-propylbenzene, while the area at 2.60 ppm is not impacted by this contamination. The amount of product from electrolysis experiments was in a range that could be impacted by residual plastic contamination or extraction solvent (in the case of hexane or cyclopentane). Thus, the percent error at 0.95 ppm will usually be larger than the one at 2.60 ppm. The prefactor in front of the percent errors ranges from two to three, increasing the sensitivity to percent errors in NMR peak areas. As an example, a 3% larger area at 0.95 ppm and negligible percent error at 2.60 ppm will result in 6–9 % absolute error on the deuterated percentage. This high sensitivity of the deuterated fraction to errors in peak area obtained from ^{1}H NMR made MS the preferred method for isotopic quantification.

2.5 Voltammetry

Voltammetric experiments were performed in either one- or two-compartment cells with either Al or Pt anodes (specific details given where data is presented). After assembly of the cell and injection of the electrolyte, the electrolyte was purged for at least 10 min with 20 sccm gas

(N_2 unless otherwise indicated). After 10 min with gas still flowing, the cathode was held at -2 V for 5 min to reduce any native oxide. After this hold, the potential was set to the open-circuit voltage (OCV) for several minutes, and the gas flow was turned off. Then, the voltammetric experiment was performed, usually at 100% IR compensation. We do note that this compensation is via software, and 100% compensation works well if the current density magnitude is below 10 mA/cm^2 and the scan rate is not too high. We did observe that the OCV would become more positive over the course of several minutes. Depending on how long OCV was held, we noticed a reduction peak that would grow as this time was increased. We hypothesize this peak comes from the reduction of trace O_2 in the system that slow builds up after the -2 V hold. Although visible, the impact of this trace O_2 on the resulting voltammograms was very minimal. In most cases, a background experiment was performed without substrate. Immediately afterwards, the substrate would be added directly to the cell with the gas flow turned back on to prevent lab air from entering the cell, and the voltammetric experiment repeated after 1–2 min to allow the substrate to mix evenly in the electrolyte.

2.6 Choice of Anode Material

To improve the ease of carboxylation experiments aimed at studying the role of the solvent, sacrificial anodes were used for the counter reaction. These enabled one-compartment cells to be used and avoided unwanted oxidation products which could clutter product analysis. Previous work demonstrates how to perform electrocarboxylation without sacrificial anodes while maintaining product selectivity, so the results of this study should be applicable to electrocarboxylation systems with alternative anodic reactions.[7] Aluminum anodes were selected over magnesium ones because (1) high purity Al anodes were cheaper and (2) Al anodes were more easily reused than Mg ones. Mg anodes would corrode significantly during acid washes of the cell (necessary to recover all of the carboxylic acid), leaving indentations that could prevent good sealing by the O-ring in future runs, particularly during in-line GC sampling which creates a brief pressure wave when the sampling valve rotates. Al anodes were much less reactive and could be reused dozens of times.

We explored Al anodes of various purities and found that the stated purity did not have a large impact on carboxylation selectivity. Al anodes of 99.9999% purity had similar acid-to-alkane ratios as those with 99.45% purity. We ultimately ended up using a combination of 99.9999% and 99.9995% purity Al anodes for practical reasons. We observed that electrolytes containing just BF_4^- anions made Al oxidation too difficult, so all experiments had a Br^- salt to facilitate Al oxidation.

Table S1. Comparison of carboxylation selectivities between aluminum anodes of different purity. CHR = carboxylation-to-hydrogenolysis ratio.

Solvent	CHR (99.999% Al)	CHR (99.45% Al)
**DMF (1)**	1.88	1.85
**DMF (2)**	2.28	2.32
**MeCN**	0.57	0.54

Experimental conditions: -5 mA/cm^2 for 30 min, 20 sccm CO_2, 100 mM **1a**, 100 mM TBA-BF_4, 25 mM $MgBr_2$. We note that these results were collected prior to full procedure optimization, so these results should only be compared between the different anode purities.

2.7 Experiments in Propylene Carbonate

Carboxylation experiments were conducted at constant current in propylene carbonate as a solvent. Product quantification was made difficult by (1) the tendency for propylene carbonate to partition into Et_2O, (2) the low volatility of propylene carbonate, which made rotary evaporation infeasible, and (3) decomposition by the workup conditions leading to an alcohol product that overlapped the carboxylic acid peak on the GC-MS.

To get around these limitations, a special workup procedure was developed for propylene carbonate incorporating partition coefficients for the carboxylate partitioning derived from UV-vis absorbance measurements (Ocean Optics). The cell was washed with 3.5 mL 0.1 M HBr and added to the removed electrolyte. This solution was basified with a minimal amount of 1 M Na_2CO_3. Four hexane extractions were performed on this mixture (1x 3.5 mL, 3x 1.75 mL hexane); the first hexane extraction was used to wash out the cell prior to addition to the basic electrolyte solution. We observed a triphasic system (water, propylene carbonate, and hexane) during these extractions. The combined hexane fractions were washed three times with 3.5 mL MilliQ water, and another hexane extraction was performed on these MilliQ water washes and combined with the other hexane layers. The goal of these steps was to isolate the substrate and alkane from TBA and propylene carbonate. To begin isolation of the carboxylic acid, the basic electrolyte solution was extracted three times with Et_2O (1x 3.5 mL, 2x 1.75 mL) with the first 3.5 mL Et_2O used to wash out the cell before addition to the basic electrolyte solution. In the presence of propylene carbonate and TBA cations, some of the carboxylate product will partition into the Et_2O layer. The combined Et_2O layers were extracted three times with 3.5 mL 1 M Na_2CO_3 to recover nearly all of the lost carboxylate. A 3.5 mL Et_2O extraction was performed on the combined basic aqueous layers. Then, the basic aqueous layers were combined with the basic electrolyte layers and carefully acidified with a minimal amount of 12 M HCl to minimize dilution. Once the aqueous fraction was acidified, it was extracted three times with Et_2O (2x 3.5 mL, 1x 1.75 mL). GC-MS of this fraction showed an alcohol decomposition product from propylene carbonate that significantly overlapped with the carboxylic acid, so ^1H NMR was used instead to quantify the carboxylic acid.

Further tests of propylene carbonate were not performed due to difficulties with DFT. Specifically, deprotonation of the methyl side group resulting in breakage of the cyclic carbonate structure and transference of the negative charge to an oxygen atom. Because of the large structural deformation, the energy is likely not representative of the transition state in the same way it is for the other carbanion structures. Thus, a reliable descriptor for propylene carbonate could not be established, and its moderate CHR suggests that it is not a good solvent candidate for electrocarboxylation. Moreover, a poorer FE closure was also observed with propylene carbonate in comparison to all of the other solvents, indicating that additional solvent decomposition pathways may occur.

3 Solvent-Based Descriptor for Carboxylation Selectivity

3.1 Computational Methodology

Density functional theory (DFT) calculations were run in Gaussian16.[8] The M06-2X/def2-TZVPD[9–11] level of theory was primarily used. The M06-2X/def2-TZVP level of theory has been demonstrated to work well for calculating energies of organic radicals.[12] An additional set of diffuse functions were added to def-TZVP improve accuracy for anions; these basis functions were input manually into the Gaussian input file and obtained from the Basis Set Exchange.[13] Results for many molecules were also obtained with the less computationally intensive B3LYP/6-31++G(d,p)[14–17] level of theory and generally agreed well with the results from M06-2X/def2-TZVPD. Geometry optimization was performed with tight convergence thresholds for all molecules in both the gas phase and with PCM solvation.[18] Frequency calculations were performed on these optimized structures to confirm local minima and thermodynamic properties. Gibbs free energies were obtained at 298.15 K using the standard thermochemistry output in Gaussian. A SuperFine integration grid was used only for calculations with M06-2X/def2-TZVPD, as it was found to improve convergence for some molecules. The nosymm keyword was added to achieve convergence when required.

The reactivity of solvent molecules occurs within the solvated environment of the liquid electrolyte. This environment was captured to a first approximation with PCM solvation. The solvent identity was chosen to be the same as the molecule under consideration if already present within Gaussian16's default list (MeCN, DMF, DMSO, DMA, PrN, NMF). For GBL, DMI, NMP, and PC, which did not have solvent parameters already present, DMA was used as the solvent to provide roughly similar dielectric properties. The inclusion of solvation significantly impacts the energies of the anions. However, for the primary carbanion of *n*-propylbenzene, we observed only a small dependence on the identity of the solvent, mostly related to its dielectric constant. The variation in the energy difference between the anion and neutral molecule for *n*-propylbenzene across MeCN, DMF, DMSO, DMA, and PrN was only 4 kJ/mol, indicating that substituting DMA for the aforementioned solvents is a reasonable approximation for a descriptor. We do note that the anion energies changed noticeably more for lower polarity solvents such as THF, 1,4-dioxane, and anisole, so DMA is not a universal substitution. Application of this computational methodology to new solvent molecules should at least use a solvent with similar dielectric constant for PCM calculations.

3.2 Computational Descriptors

Computational descriptors for solvent reactivity were obtained from calculated free energies of acid-base and hydrogen abstraction reactions. To avoid calculating free energies of solvated protons and free hydrogen radicals, a common reference molecule was used. The choice of reference molecule is rather arbitrary, as the specific choice does not influence the relative values of the descriptors among solvents.

For assessing solvent deprotonation, the following acid-base reaction was used:

$$R^{-} + H - Solv \rightarrow R - H + Solv^{-}, \qquad \Delta G_{an}$$

where R^- is reference base, R-H is the protonated reference base, H-Solv is the solvent, and $Solv^-$ is the deprotonated solvent. R^- was chosen to be *n*-propylbenzene with the carbanion at the terminal alkyl position. Since many solvent molecules have several non-equivalent hydrogen atoms, a set of deprotonation free energies may exist. A composite descriptor capturing deprotonation of all C-H or N-H bonds in the solvent was also developed:

$$Q_{an} = C_S \sum_{i=1}^{N_H} n_i e^{-\frac{\Delta G_{an,i}}{RT}}$$

where Q_{an} is the composite deprotonation free energy descriptor, C_S is the molar concentration of the pure solvent in its liquid state (mol/L), N_H is the number of non-equivalent C-H and N-H bonds in the solvent, n_i is the number of identical C-H and N-H bonds of type *i*, $\Delta G_{an,i}$ is the deprotonation free energy of bond type *i* (J/mol), *T* is the temperature (K), and *R* is the ideal gas constant (8.314 $J{\cdot}mol^{-1}{\cdot}K^{-1}$). The expression for Q_{an} captures all of the contributions of each deprotonation site to give an overall value representative of the deprotonation rate of a solvent. The kinetics of deprotonation are assumed to be related to the deprotonation free energy by approximately the same scaling factor for all deprotonation sites. The identification of non-equivalent hydrogen atoms was taken as the number of unique ^{1}H NMR peaks of the solvent molecule, with n_i taken as the number of protons contributing to each peak. As an example, DMF would have $N_H = 3$ with $n_i = \{1,3,3\}$ representing one formyl proton and two sets of three methyl protons. Since all of the solvents examined in this study had one site that was much easier to deprotonate than the rest, there was not a significant difference using the most negative $\Delta G_{an,i}$ and Q_{an} to correlate experimental CHRs. Analogous expressions can be developed for hydrogen atom transfer:

$$R^{\cdot} + H - Solv \rightarrow R - H + Solv^{\cdot}, \qquad \Delta G_{rad}$$

$$Q_{rad} = C_S \sum_{i=1}^{N_H} n_i e^{-\frac{\Delta G_{rad,i}}{RT}}$$

These computational descriptors present a simplified model that captures the molecular contributions to solvent deprotonation. However, in actual electrochemical carboxylation systems, solvent deprotonation would likely occur at an electrified interface, so the presence of strong electric fields, altered solvation shells, and catalytic metal surfaces would likely impact the deprotonation free energy. Solvent deprotonation may also happen in concert with electron transfer. Accounting for these additional phenomena at electrochemical interfaces is a computationally demanding task, but as evidenced by the strong correlations with experimental data in this work, these additional phenomena are not necessarily required to understand major trends in carboxylation selectivity with respect to the solvent. The success of a simple acidity descriptor such as ΔG_{an} illustrates that solvent deprotonation is the primary factor governing carboxylation selectivity. The simplicity of these descriptors also enables screening of large sets of solvent molecules to find promising candidates. Nevertheless, these correlations are not perfect, which may reflect that other phenomena at electrochemical interfaces may have some effect.

3.3 Deuterium Exchange Descriptor

This work developed an experiment to assess the kinetics of deprotonating solvents via hydrogen-deuterium exchange. The experiment involved reducing deuterated ethanol (EtOD) at a silver cathode in a one compartment cell with an aluminum anode. The electrolyte comprised the solvent under investigation and 0.1 M TBA-BF_4. The Al anode was able to oxidize without a halide anion source in the presence of EtOD. At the cathode, EtOD is reduced to hydrogen and ethoxide:

$$EtOD + e^- \rightarrow \frac{1}{2}D_2 + EtO^-$$

The ethoxide can deprotonate solvent molecules, initiating a sequence of acid-base exchange reactions:

$$EtO^- + H - Solv \rightarrow Solv^- + EtOH$$

$$Solv^- + EtOD \rightarrow D - Solv + EtO^-$$

The net effect of these exchange reactions is to transfer deuterium from EtOD to the solvent. At the same time, aluminum cations from the oxidation of the anode are transported across the cell and inhibit these acid-base reactions:

$$EtO^- + Al(EtO)_x^{(3-x)+} \rightarrow Al(EtO)_{x+1}^{(2-x)+}, \qquad x = 0,1,2$$

The amount of deuterium ending up on the solvent at the end of the experiment is related to the rate of acid-base exchange between ethoxide and the solvent. If the rate is slow, Al^{3+} from the anode will have time to inhibit ethoxide before many exchange reactions can occur, leading to low amounts of deuterium ending up in the solvent. The exchange rates may also be influenced by mass transport affecting the reactions of deprotonated solvent with EtOD and Al^{3+} with EtO^-. Differential mass transport may exist across solvents, which could impact the observed exchange rates. To a first approximation, the diffusivities of $Solv^-$ and Al^{3+} should scale together in different solvents of similar polarities, so the effect of mass transport on the observed deuterium incorporation in the solvent may be small. Different solvation abilities of the tested solvents could also impact the exchange rates, although testing only across polar, aprotic solvents helps reduce this difference.

In this work, -5 mA/cm^2 was applied for 20 min to probe the rate of acid-base exchange reactions. An N_2 flow of 20 sccm was bubbled through the electrolyte to aide with mass transport. The electrolyte was quenched with some $NaBF_4$ to minimize and reactivity after stopping the current. The amount of deuterium can be quantified by ^{2}H NMR with $CDCl_3$ as an internal standard, providing a quantitative measure of the relative acidity among all deprotonation sites on a solvent. This descriptor was found to agree well with DFT in identifying the most acidic site on each solvent and quantitatively ranking the acidities between different solvents (**Figure S28**).

4 Additional Figures and Discussion

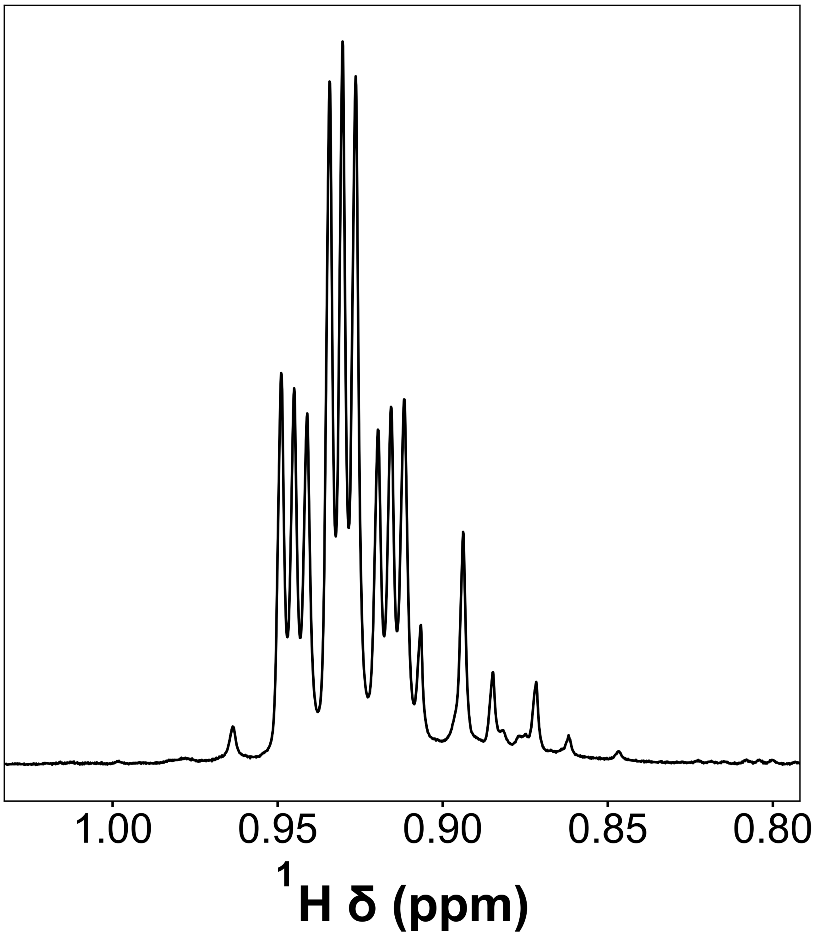

Figure S4. Zoom-in of the terminal hydrogen peak of **1b** after carboxylation in MeCN-d_3. The presence of a deuterium nucleus introduces a set of triplets on each peak in a roughly 1:1:1 ratio because its nuclear spin is one. We did observe that the clarity of the splitting depended on the deuterated solvent used to collect the ^{1}H NMR spectrum; in this case, the NMR solvent was $CDCl_3$. Reaction conditions: Undivided cell, Ag cathode, Al anode, -2.37 V vs. $Me_{10}Fc^{0/+}$ until 8 C passed, 25 mM $MgBr_2$, 0.1 M TBA-BF_4, 20 mM **1a**, MeCN-d_3, 20 sccm CO_2.

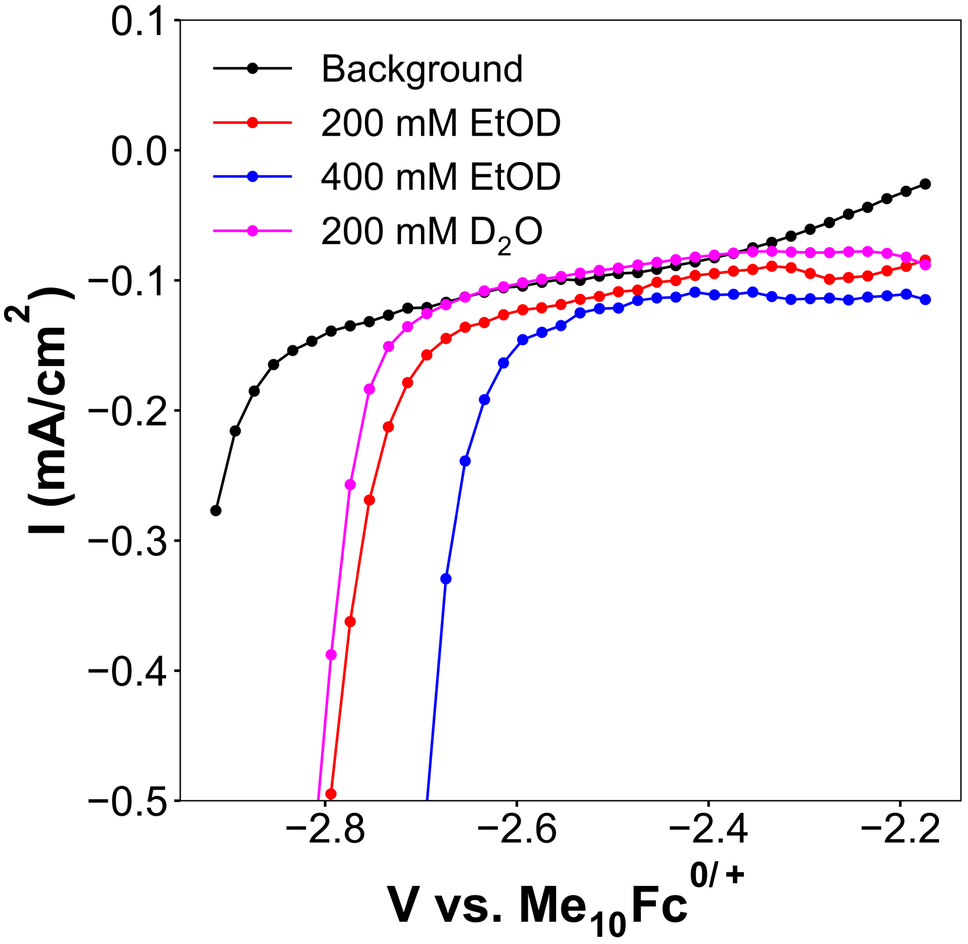

Figure S5. Staircase voltammograms of deuterated additives in DMF. Experimental conditions: Ag cathode, Al anode, undivided cell, 90 mM TBA-BF_4, 10 mM TBA-Br, 2.5 mL DMF. Acquisition parameters: 20 mV steps for 30 s. The last 50% of each step was averaged and recorded.

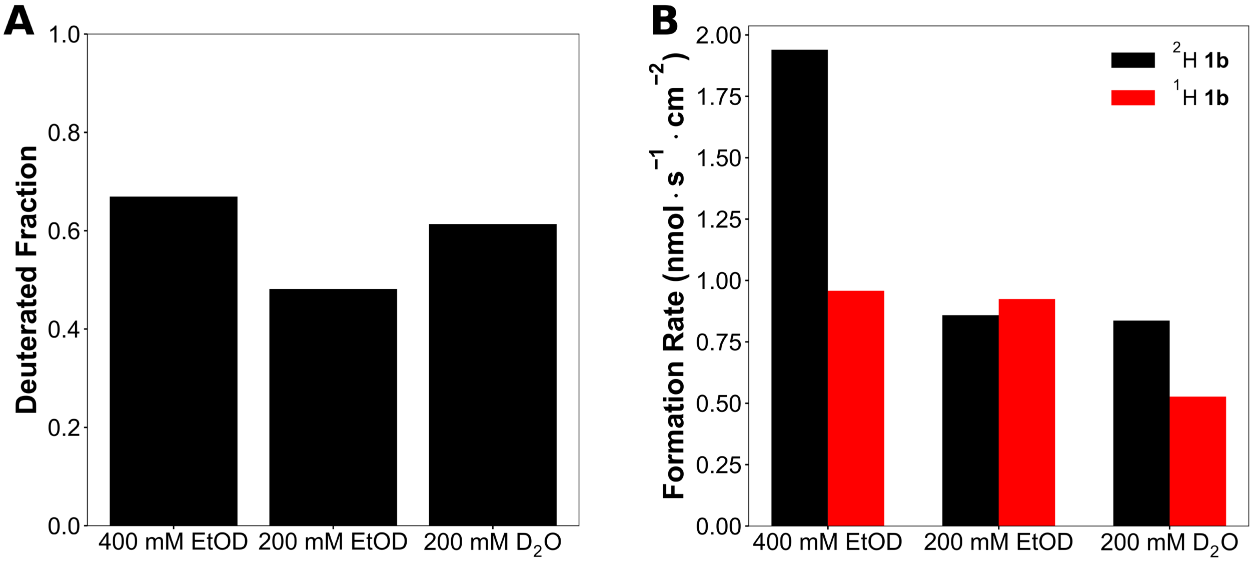

Figure S6. Effect of varying deuterated additive concentration and identity on (**A**) the fraction of deuterated **1b** and (**B**) the formation rates of protonated and deuterated **1b**. Reaction conditions: Ag cathode, Al anode, undivided cell, 20 mM **1a**, 90 mM TBA-BF_4, 10 mM TBA-Br, 20 sccm N_2, 2.5 mL DMF, -1.9 V vs. $Me_{10}Fc^{0/+}$.

Staircase voltammetry (SV) was chosen over linear sweep voltammetry (LSV) to determine onset potentials for deuterated additive reduction because SV helped reduce capacitive contributions to the current. This removal facilitates the observation of Faradaic onset for additive reduction as shown above (**Figure S5**). For 400 mM EtOD, this onset occurs around -2.6 V, making this voltage the most cathodic that could be tested with 400 mM EtOD without forming adsorbed deuterium. EtOD and D_2O work comparably well in terms of producing deuterated **1b** (**Figure S6**). EtOD was selected over D_2O because while D_2O is reduced at more cathodic potentials than EtOD in DMF on silver, OD^- can react with DMF to form deuterated products which could convolute the origin of deuterium in deuterated **1b**.

Reduction of **1a** in the presence of either 200 mM or 400 mM EtOD can give rise to intermediate deuterated fractions of the hydrogenolysis product, which facilitates tracking changes in this metric (i.e. both increases and decreases to the deuterated fraction are readily measurable) (**Figure S6A**). The doubling of the EtOD concentration roughly doubles the formation rate of deuterated **1b** while not having much of an effect on the formation rate of protonated **1b** (**Figure S6B**). The use of 400 mM EtOD was selected somewhat arbitrarily over 200 mM for this study.

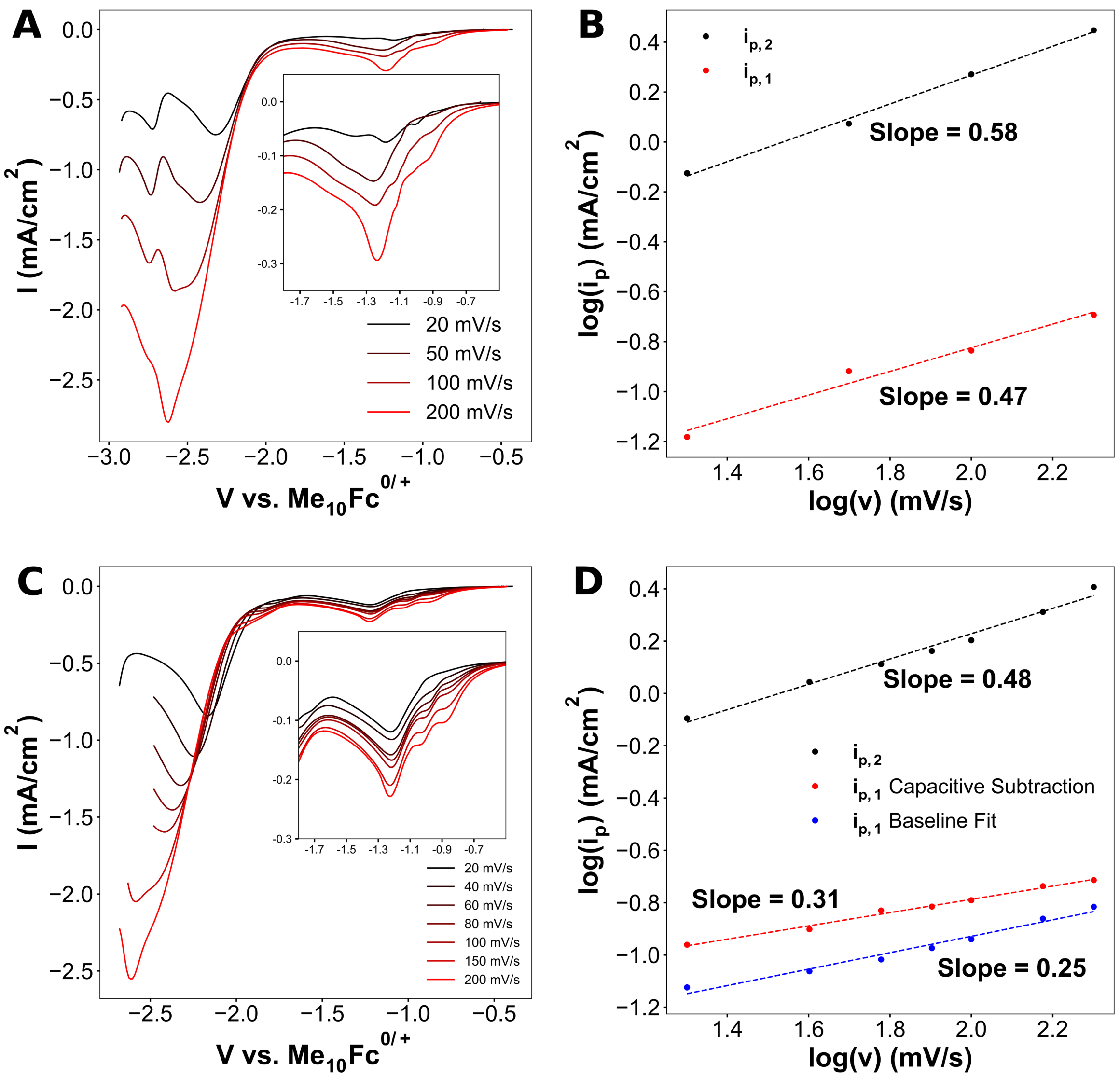

Figure S7. Linear sweep voltammograms of **1a** over a silver cathode in DMF and the scan rate dependencies of the peak currents. (**A**) LSVs and (**B**) scan-rate dependencies collected sequentially in an undivided cell with a Pt anode. Electrolyte: 100 mM TBA-BF_4, 10 mM **1a**. (**C**) LSVs and (**D**) scan-rate dependencies collected sequentially in a divided cell with a Daramic separator and Pt anode: Catholyte and anolyte: 100 mM TBA-BF_4, 10 mM **1a**, 2.5 mL DMF.

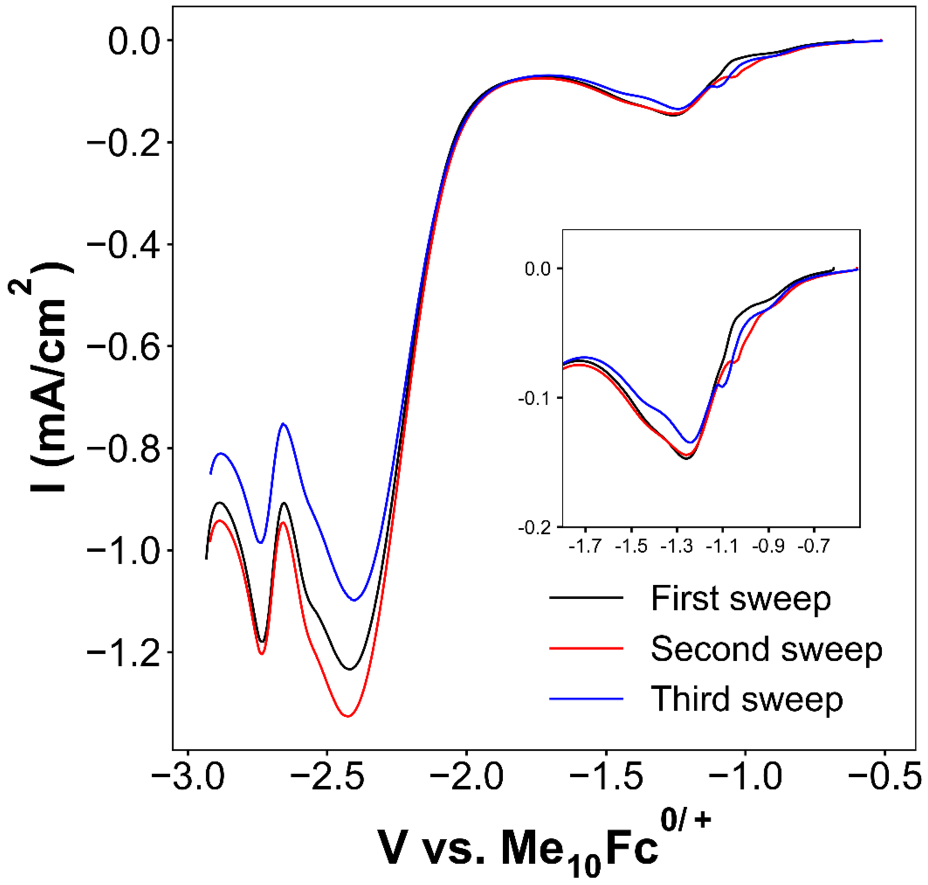

Figure S8. Repeatability of linear sweeps across consecutive experiments at 50 mV/s. These sweeps were conducted as part of the scan rate dependencies shown in **Figure S7A**. The first and second sweeps were done consecutively at the beginning of the run, while the third sweep was conducted after all of the other linear sweeps were performed. Experimental conditions are the same as those in **Figure S7A**.

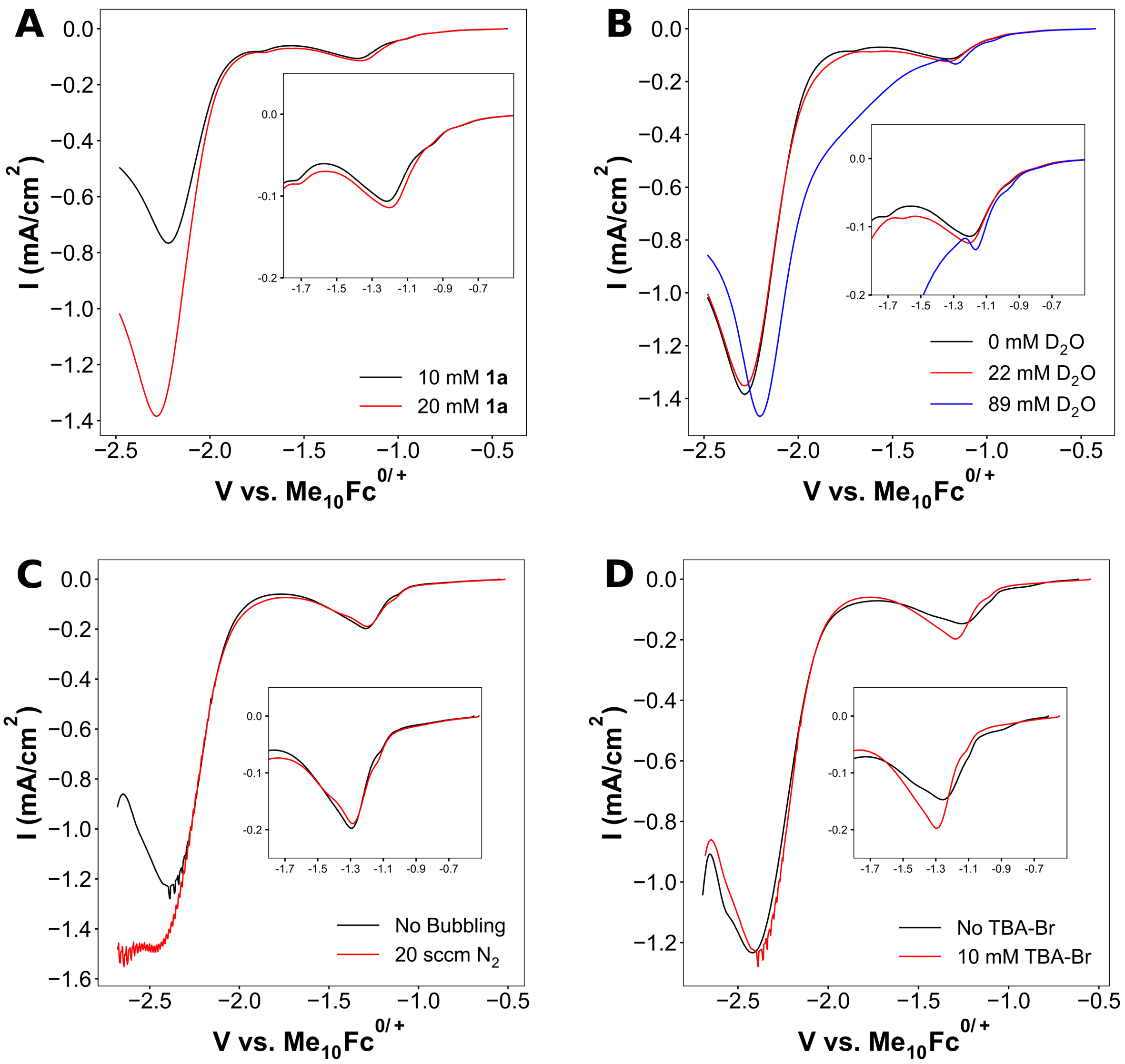

Figure S9. Impact of reaction parameters on the linear sweep voltammograms of **1a** in DMF on a silver cathode. (**A**) Effect of the concentration of **1a**. (**B**) Effect of D_2O concentration. (**C**) Effect of forced convection (with and without bubbling N_2). (**D**) Impact of TBA-Br. Standard reaction conditions for **A-B**: Divided cell, Ag cathode, Pt anode, Daramic separator. Catholyte and anolyte: 100 mM TBA-BF_4, 10 mM **1a**, 2.5 mL DMF. Standard reaction conditions for **C-D**: Undivided cell, Ag cathode, Al anode, 90 mM TBA-BF_4, 10 mM TBA-Br, 10 mM **1a**, 2.5 mL DMF (the No TBA-Br experiment in panel **D** had a Pt anode with 100 mM TBA-BF_4).

The reductive voltammogram of **1a** in DMF shows a small cathodic peak around -1.3 V, a larger second cathodic peak around -2.4 to -2.5 V, and a third peak around -2.75 V (**Figure S7A**). The third peak is beyond the range of voltages typically needed for carboxylation, so it is not considered further in this work. The second cathodic peak has roughly a $v^{0.5}$ dependence, which is indicative of a mass-transport limited peak (**Figure S7B**).[19] There is some loss of peak current upon repeated experimentation in the same cell, but the error (10–15%) is not large enough to affect the interpretation the scan-rate order dependence (**Figure S8**). The peak currents are also large enough that subtraction of an appropriate background current is not needed. The second

cathodic peak current does depend on the concentration of **1a** (approximately first-order) and the presence of forced convection, providing further support that the peak is related to mass transport limitations of **1a** (**Figure S9A** and **S9B**). Also consistent with this conclusion is the lack of dependence on the amount of D_2O and presence of Br^- (**Figure S9C** and **S9D**).

The repeatability of the first cathodic peak is better across experiments within the same cell (**Figure S8**), but its scan-rate dependence varies more between the two sets of experiments (**Figure S7B** and **S7D**). These peak currents are small enough to warrant baseline subtraction. For the divided cell experiment, two methods were used to estimate the peak current of the first cathodic peak: the baseline subtraction tool in the EC-Lab software and subtraction of the capacitive current estimated from LSVs without **1a** present. The peak currents and scan-rate dependencies are somewhat different between the two methods, although a greater difference lies between the divided- and undivided-cell experiments. Notably, this peak current does not exhibit a first-order dependence on the scan-rate, which would be indicative of a classical adsorption-limited reaction.[19] The first cathodic peak current is relatively unaffected by the concentration of **1a**, the concentration of D_2O, forced convection, or the presence of Br^- anions (**Figure S9**). In constant current experiments, the total current densities at potentials past the first reduction peak have a weak dependence on the applied potential (**Figure S22**). The reduction of adsorbed bromine atoms can be ruled out since bromide is desorbed at potentials more cathodic than -1.40 V vs. $Me_{10}Fc$ on silver in DMF.[20] This peak does arise from **1a**, as GC-MS analysis of **1a** indicates that any impurities are below 1 mol%. If such an impurity were causing this peak, it would have to be under mass transport control, which is inconsistent with our observations.

These observations are most consistent with an adsorption-site-limited process involving the reductive cleavage of the C-Br bond with subsequent binding of the organic radical to the surface. Adsorption of organic moieties to electrode surfaces from the reduction of organic halides has also been observed in the literature.[21,22] With 89 mM D_2O, while the peak current is relatively unchanged, the post-peak current is noticeably larger. As observed during chronoamperometric experiments, the addition of protic additives such as EtOD and D_2O increase the overall current at potentials between the first and second cathodic peaks. These additives may help clear the surface of intermediates by providing protons for reductive C-H bond formation. However, this process is not a standard adsorption one as indicated by the scan-rate dependencies all being much less than unity. This deviation may suggest a more complex adsorption process is occurring and further work would be needed to fully elucidate the process responsible for this peak which is beyond the scope of this work.

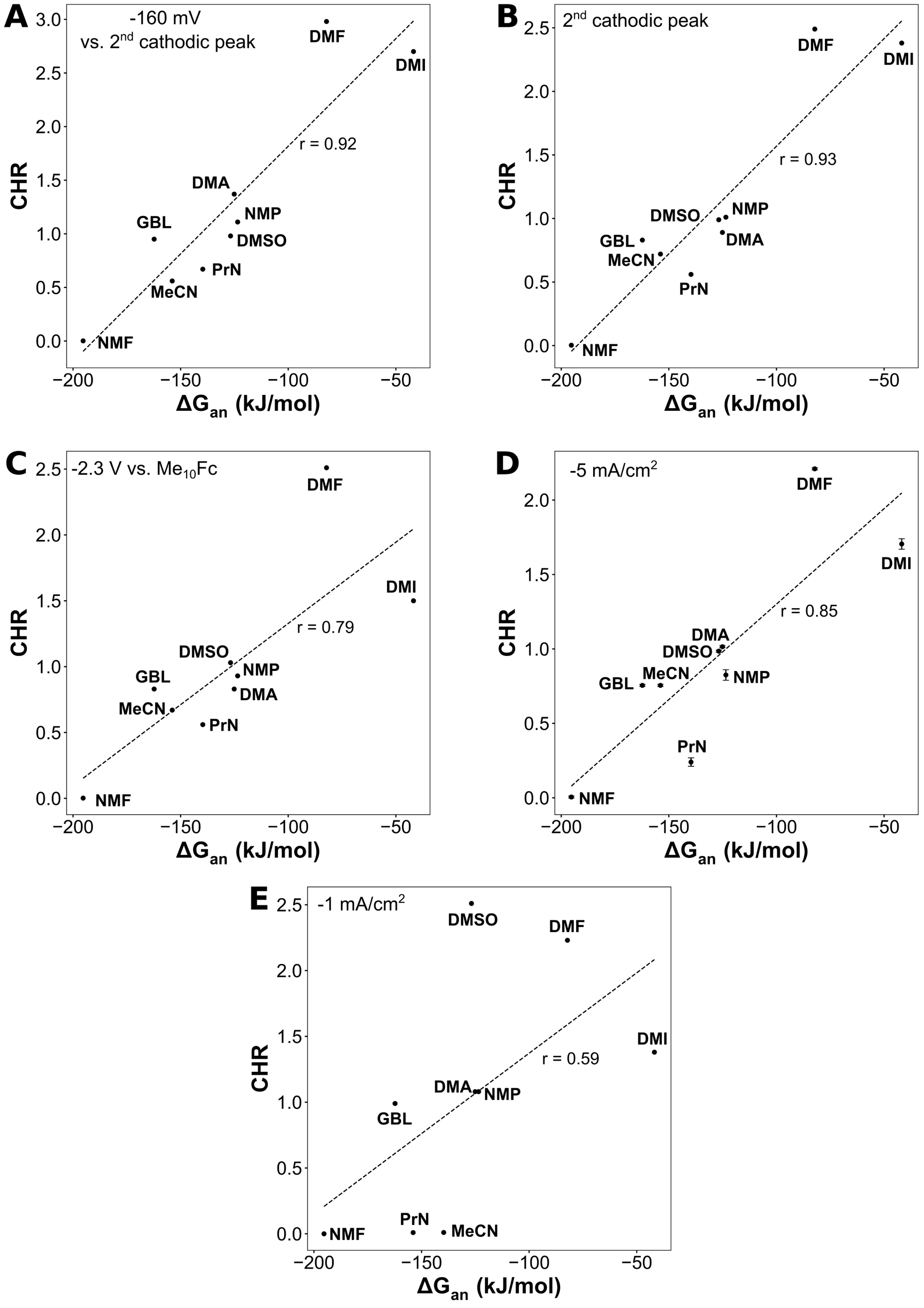

Figure S10. Correlations of ΔG_{an} (M06-2X/def2-TZVPD + PCM) with CHR for various experimental conditions. (**A**) Constant potential at -160 mV vs. the 2nd cathodic peak in each solvent. (**B**) Constant potential at the 2nd cathodic peak in each solvent. (**C**) Constant potential at -2.3 V vs. $Me_{10}Fc$. (**D**) Constant current at -5 mA/cm^2. Error bars represents standard deviations from duplicate measurements. (**E**) Constant current at -1 mA/cm^2. Reaction conditions for **A-C**: Ag cathode, Al anode, undivided cell, 20 mM **1a**, 90 mM TBA-BF_4, 10-15 mM TBA-Br (increased from base value of 10 mM to keep total cell voltage within potentiostat's limits), 2.5 mL solvent, 20 sccm CO_2, passed 4 C. Reaction conditions for **D-E**: Ag cathode, Al anode, undivided cell, 100 mM **1a**, 100 mM TBA-BF_4, 25 mM $MgBr_2$, 2.2 mL solvent, 20 sccm CO_2, 6 C passed (-5 mA/cm^2) or 3.6 C passed (-1 mA/cm^2). The Pearson correlation coefficient is given as r.

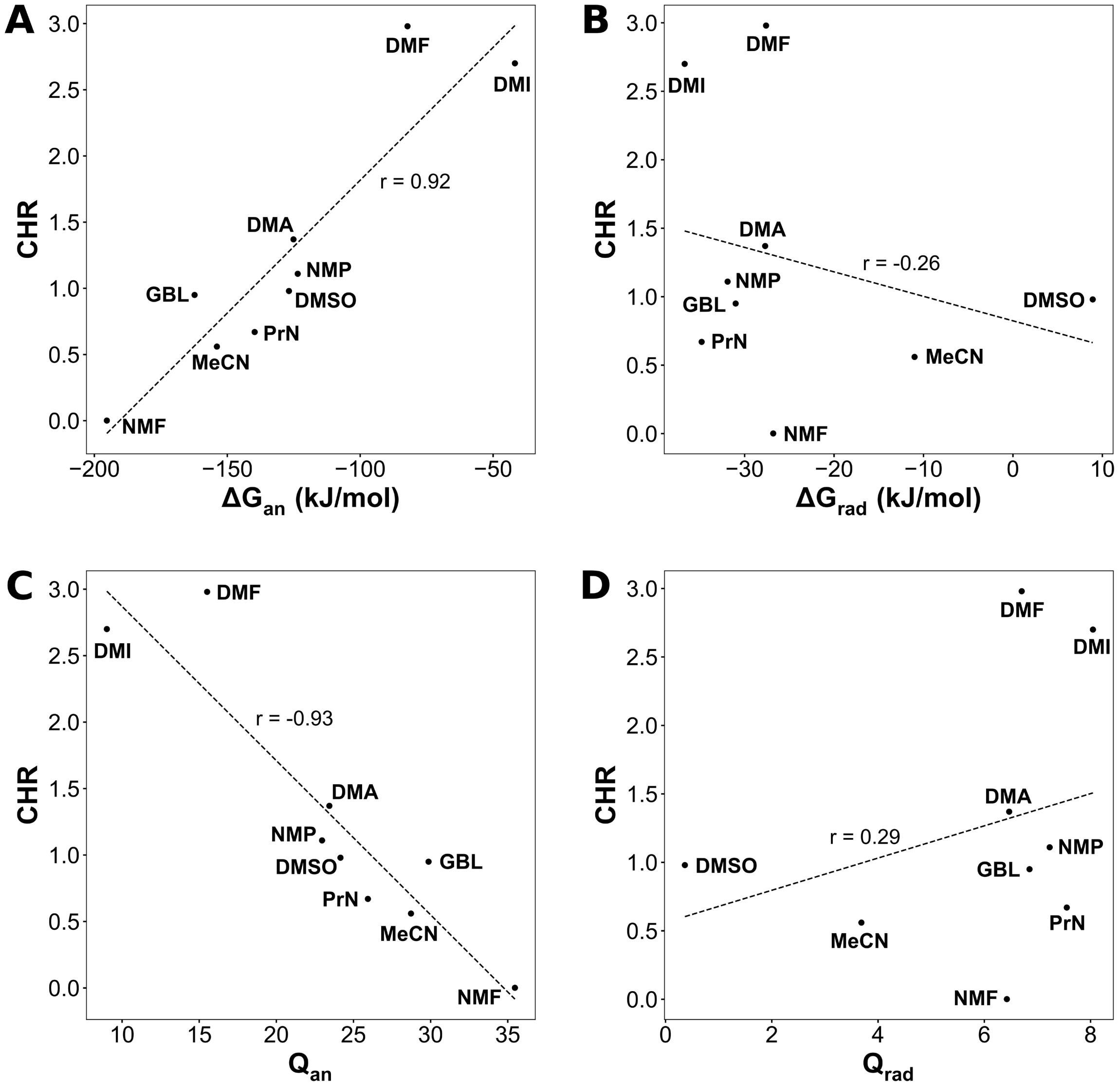

Figure S11. Comparison of computational descriptors (M06-2X/def2-TZVPD + PCM) with the CHR at -160 mV vs. the 2nd cathodic peak: (**A**) ΔG_{an}, (**B**) ΔG_{rad}, (**C**) Q_{an}, and (**D**) Q_{rad}. The Pearson correlation coefficient is given as *r*. Reaction conditions for CHR: Ag cathode, Al anode, undivided cell, 20 mM **1a**, 90 mM TBA-BF_4, 10-15 mM TBA-Br (increased from base value of 10 mM to keep total cell voltage within potentiostat's limits), 2.5 mL solvent, 20 sccm CO_2, passed 4 C.

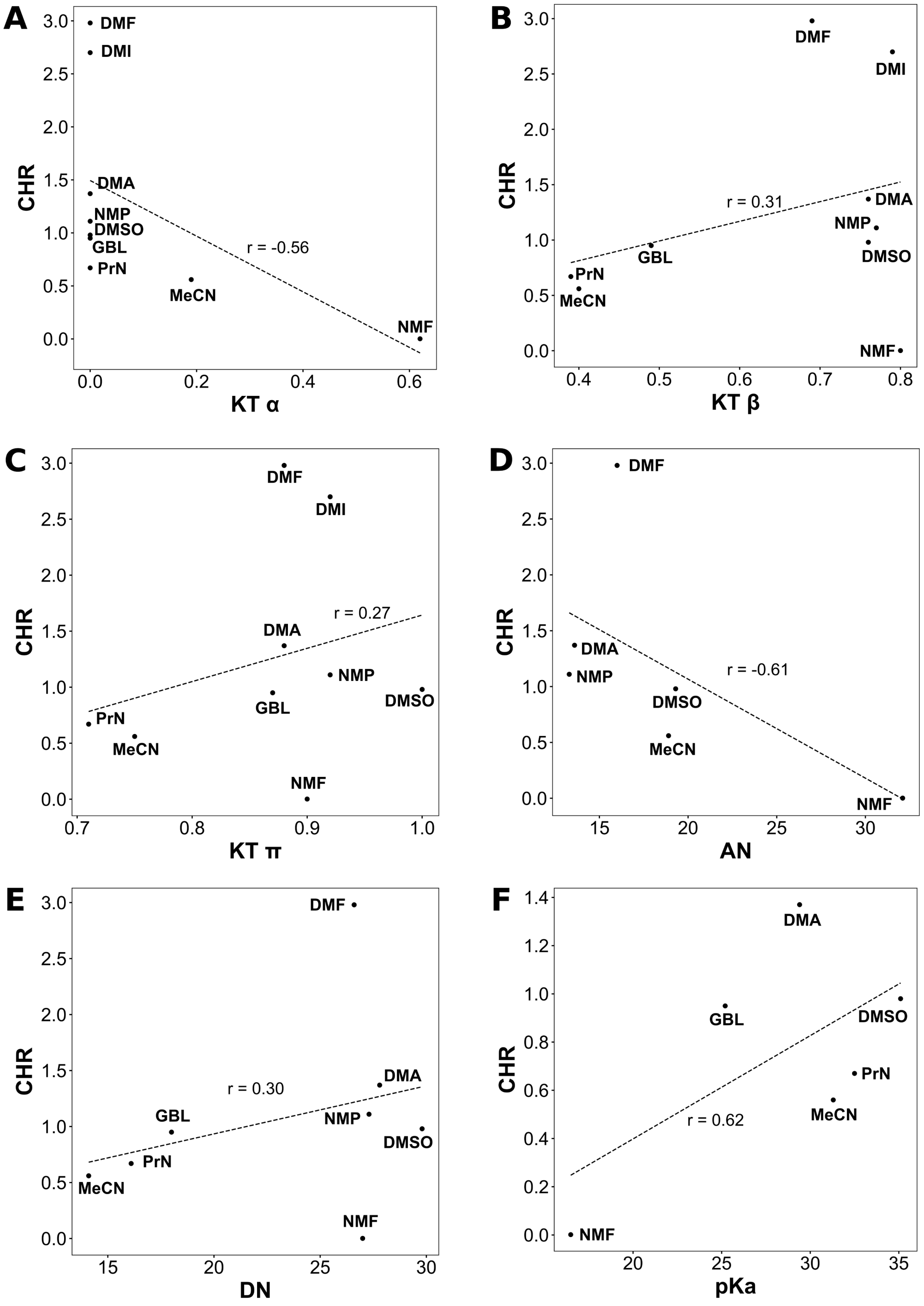

Figure S12. Correlations between experimental solvent properties and CHR at -160 mV vs. 2nd cathodic peak potential: (**A**) Kamlet-Taft (KT) α, (**B**) KT β, (**C**) KT π, (**D**) Gutmann acceptor number (AN), (**E**) Gutmann donor number (DN), (**F**) pKa. The Pearson correlation coefficient is given as *r*. Reaction conditions for CHR: Ag cathode, Al anode, undivided cell, 20 mM **1a**, 90 mM TBA-BF_4, 10-15 mM TBA-Br (increased from base value of 10 mM to keep total cell voltage within potentiostat's limits), 2.5 mL solvent, 20 sccm CO_2, passed 4 C.

Table S2. Compilation of experimental solvent descriptors from the literature including pKa, Kamlet-Taft (KT) solvatochromic parameters, and Gutmann acceptor (AN) and donor (DN) numbers.

Solvent	pKa	KT α	KT β	KT π	AN	DN
DMA	29.4[23,a]	0.00[24]	0.76[24]	0.88[24]	13.6[24]	27.8[24]
DMF	-[b]	0.00[24]	0.69[24]	0.88[24]	16.0[24]	26.6[24]
DMI	-[b]	0.00[25]	0.79[25]	0.92[25]	-[f]	-[f]
DMSO	35.1[26,c]	0.00[24]	0.76[24]	1.00[24]	19.3[24]	29.8[24]
GBL	25.2[26,cd]	0.00[24]	0.49[24]	0.87[24]	17.3[24]	18.0[24]
MeCN	31.3[26,c]	0.19[24]	0.40[24]	0.75[24]	18.9[24]	14.1[24]
NMF	16.48[e]	0.62[24]	0.80[24]	0.90[24]	32.1[24]	27.0[24]
NMP	-[b]	0.00[24]	0.77[24]	0.92[24]	13.3[24]	27.3[24]
PrN	32.5[26,c]	0.00[24]	0.39[24]	0.71[24]	-[f]	16.1[24]

[a] Determined in D_2O

[b] Experimental and alculated value not available

[c] Determined in DMSO

[d] pKa of delta-valerolactone in DMSO used instead

[e] Calculated (Chem Axon)

[f] Experimental value not available

In many cases, properties of a chemical reaction in solution can be correlated to experimentally derived solvent properties such as Kamlet-Taft parameters[27–29] and Gutmann numbers.[24] From these two types, acidity parameters show some degree of correlation with CHR but not nearly as strong as the computational descriptors (**Figure S12**). A major problem with these descriptors is that in some cases, they are not sensitive enough to differentiate solvents. As an example, while the Kamlet-Taft α parameter shows some correlation with CHR, most of the solvents examined have $\alpha = 0$. This parameter could predict CHR up to a certain point but is not useful to find the most selective solvents. Solvent pKa also shows a decent correlation with CHR, but it is not readily measurable for the most basic solvents. These situations highlight the advantage of using a computational descriptor based on the molecular structure of the solvent since it can be calculated in principle for any solvent molecule.

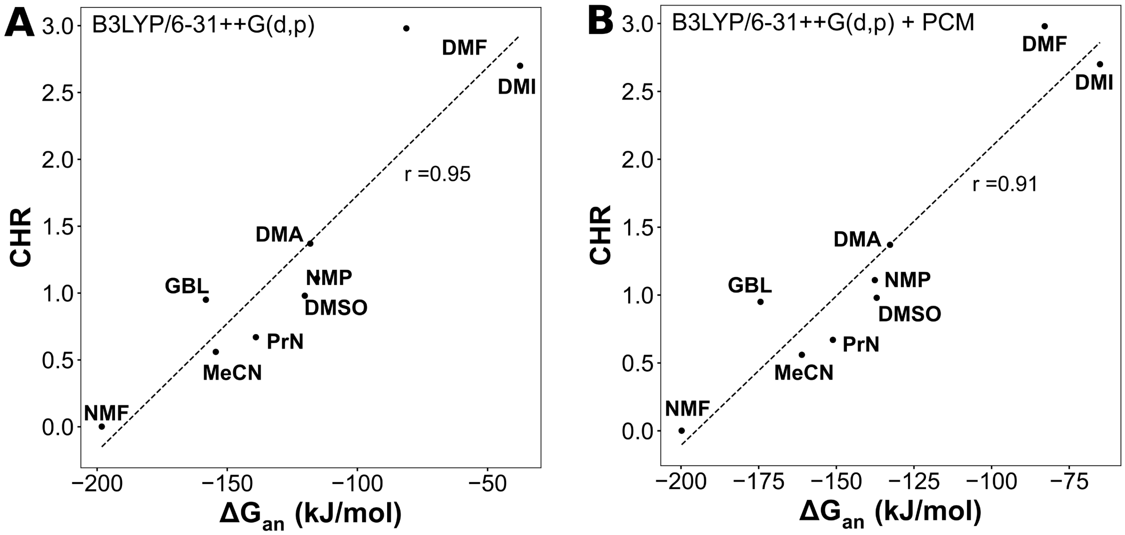

Figure S13. Correlations between CHR at -168 mV vs. 2nd cathodic peak and *ΔG*$_{an}$ from other DFT methodologies: (**A**) B3LYP/6-31++G(d,p) and (**B**) B3LYP/6-31++G(d,p) with PCM solvation. The Pearson correlation coefficient is given as *r*. Reaction conditions for CHR: Ag cathode, Al anode, undivided cell, 20 mM **1a**, 90 mM TBA-BF_4, 10–15 mM TBA-Br (increased from base value of 10 mM to keep total cell voltage within potentiostat's limits as needed), 2.5 mL solvent, 20 sccm CO_2, passed 4 C.

Less computationally intensive methods may be used to generate *ΔG*$_{an}$ values with strong correlations to experimental CHRs. Strong correlations were obtained using the B3LYP/6-31++G(d,p) level of theory both with and without PCM solvation (**Figure S13**). This result suggests that the key property of the solvent is related to its molecular structure and not its solvating ability. The ability to use cheaper computational methods bodes well for using the *ΔG*$_{an}$ descriptor for rapid solvent screening.

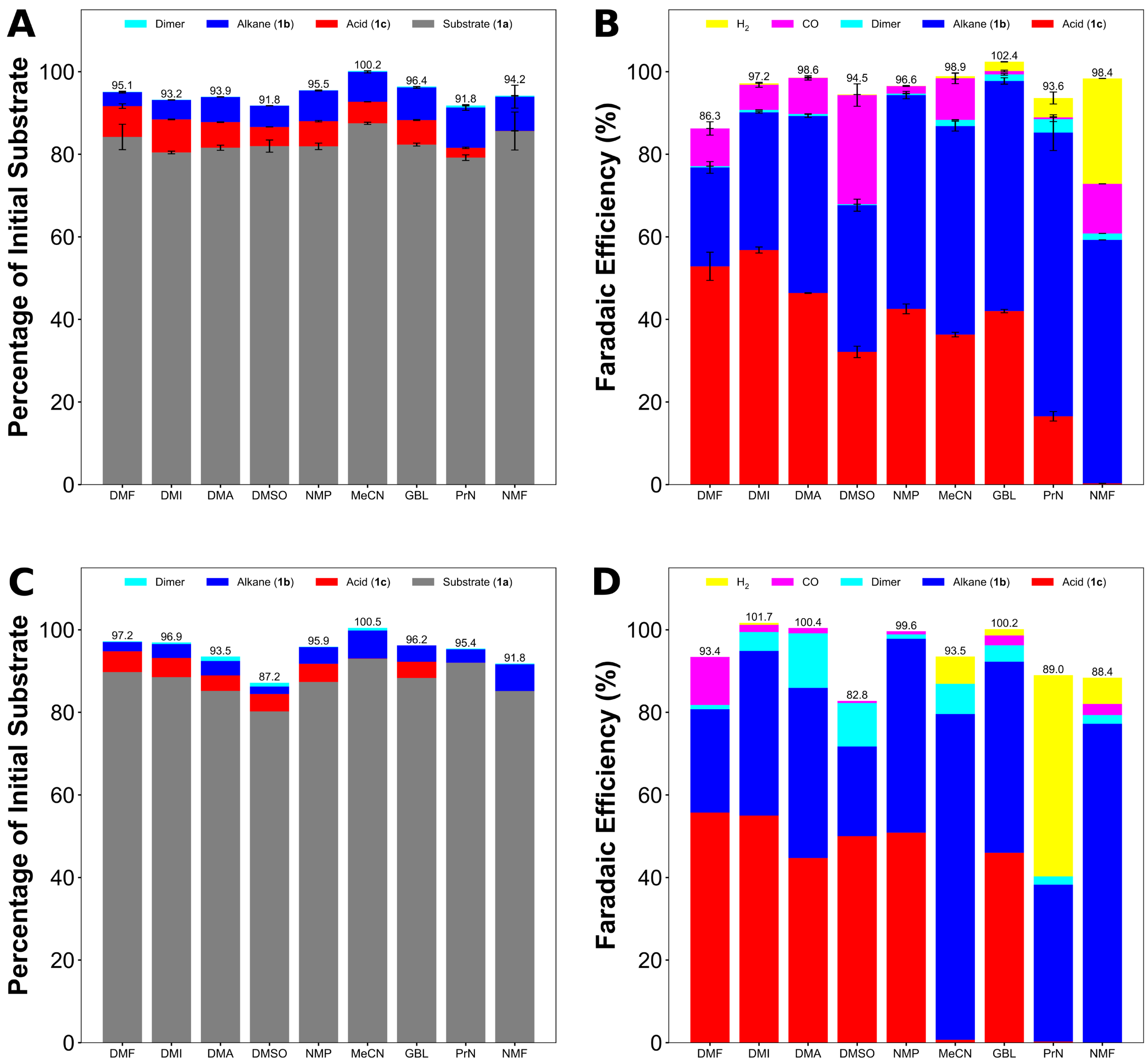

Figure S14. Mass and charge balances for carboxylation of **1a** under constant current conditions. (**A**) Mass balance, represented as the percentage of each species relative to the initial amount of **1a**, at -5 mA/cm^2. (**B**) Charge balance, represented as the Faradaic efficiency (FE), describing the percentage of the total passed charge toward each product at -5 mA/cm^2. (**C**) Mass balance at -1 mA/cm^2. (**D**) Charge balance at -1 mA/cm^2. Reaction conditions: Ag cathode, Al anode, undivided cell, 100 mM **1a**, 100 mM TBA-BF$_4$, 25 mM MgBr$_2$, 2.2 mL solvent, 20 sccm CO_2, 6 C passed (-5 mA/cm^2) or 3.6 C passed (-1 mA/cm^2). Error bars in **A** and **B** are standard deviations from duplicate measurements.

For the majority of solvents tested, the primary products are the carboxylic acid and hydrogenolysis alkane product (**Figure S14** and **Tables S2 – S5**), which supports the initial assumption that the hydrogenolysis product is the primary side product across all solvents. In terms of closing the charge balance, the reduction of CO_2 to CO occurs to a moderate extent in most solvents. Some of the more acidic solvents also display some hydrogen evolution activity. The

comprehensive mass and charge balances across all solvents reveals that hydrogenolysis is the primary side reaction that consumes the organic halide substrate.

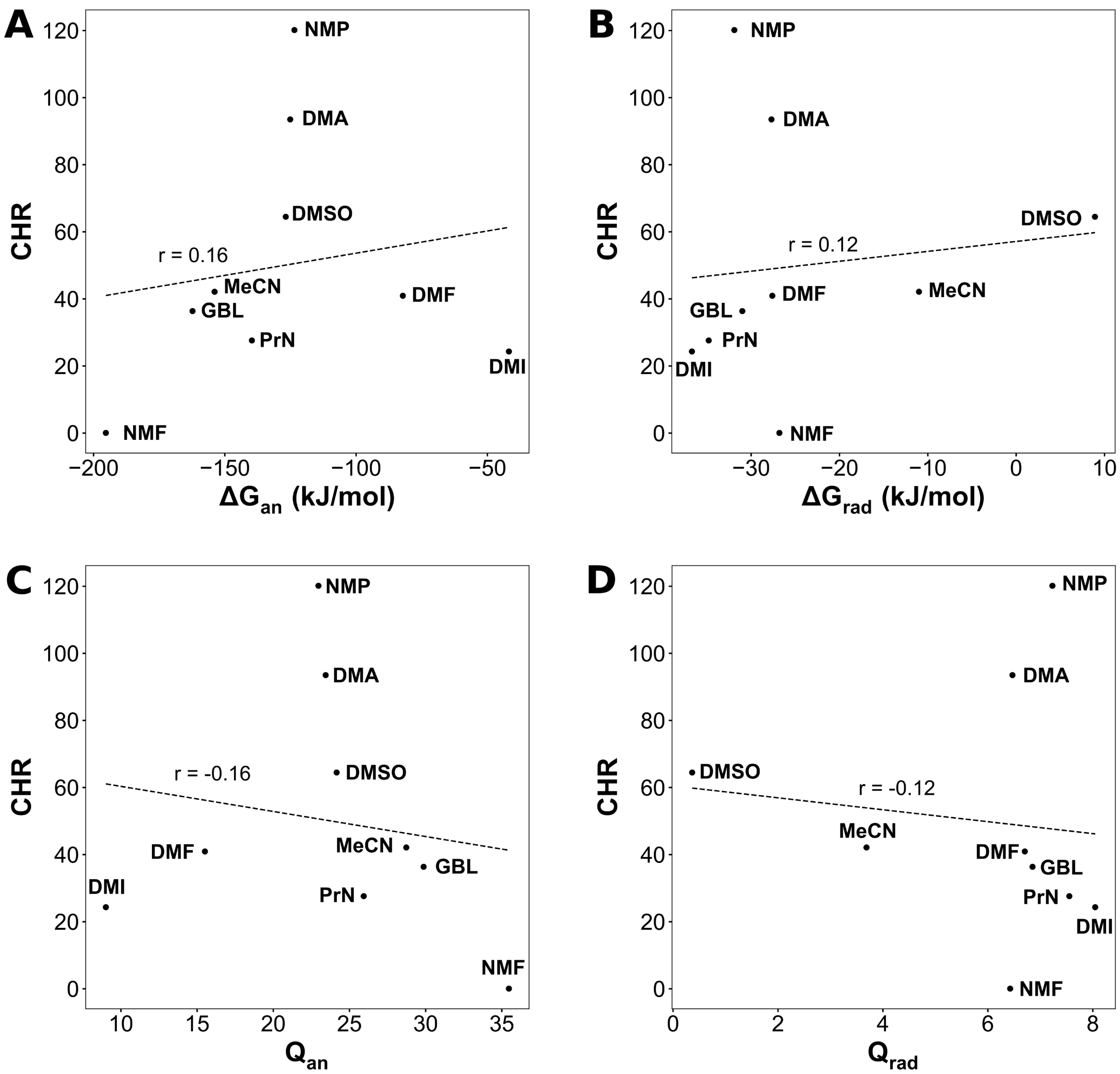

Figure S15. Correlations between experimental carboxylation-to-hydrogenolysis ratio (CHR) and computational descriptors for (1-bromoethyl)benzene (**2a**). Descriptors are (**A**) ΔG_{an}, (**B**) ΔG_{rad}, (**C**) Q_{an}, and (**D**) Q_{rad}. Descriptors were calculated assuming n-propylbenzene radicals and anions as references for convenience, although this choice does not impact the strength of the correlations. Reaction conditions for CHR: Ag cathode, Al anode, undivided cell, -270 mV of the second cathodic peak potential from an LSV in each solvent, 20 mM **2a**, 90 mM TBA-BF_4, 10–15 mM TBA-Br (increased from base value of 10 mM to keep total cell voltage within potentiostat's limits), 2.5 mL solvent, 20 sccm CO_2, passed 4 C.

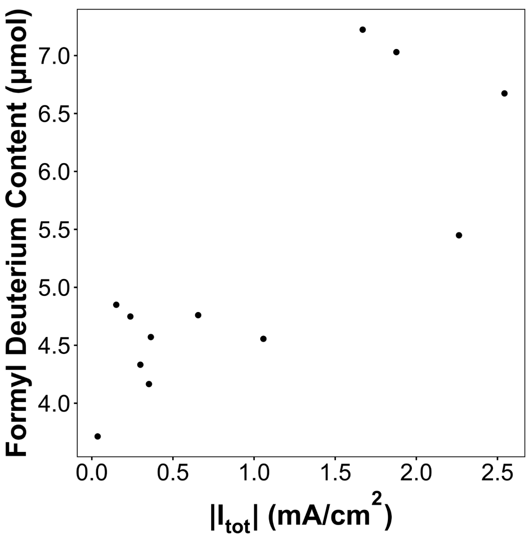

Figure S16. Comparison of the magnitude of the total current density to the amount of formyl deuterium content in DMF during reduction of **1a** in DMF with EtOD. Reaction conditions: 20 mM **1a**, 90 mM TBA-BF_4, 10 mM TBA-Br, 20 sccm N_2, DMF, 400 mM EtOD. Experiments were run for either 4 C or 1 hr, whichever condition was met first.

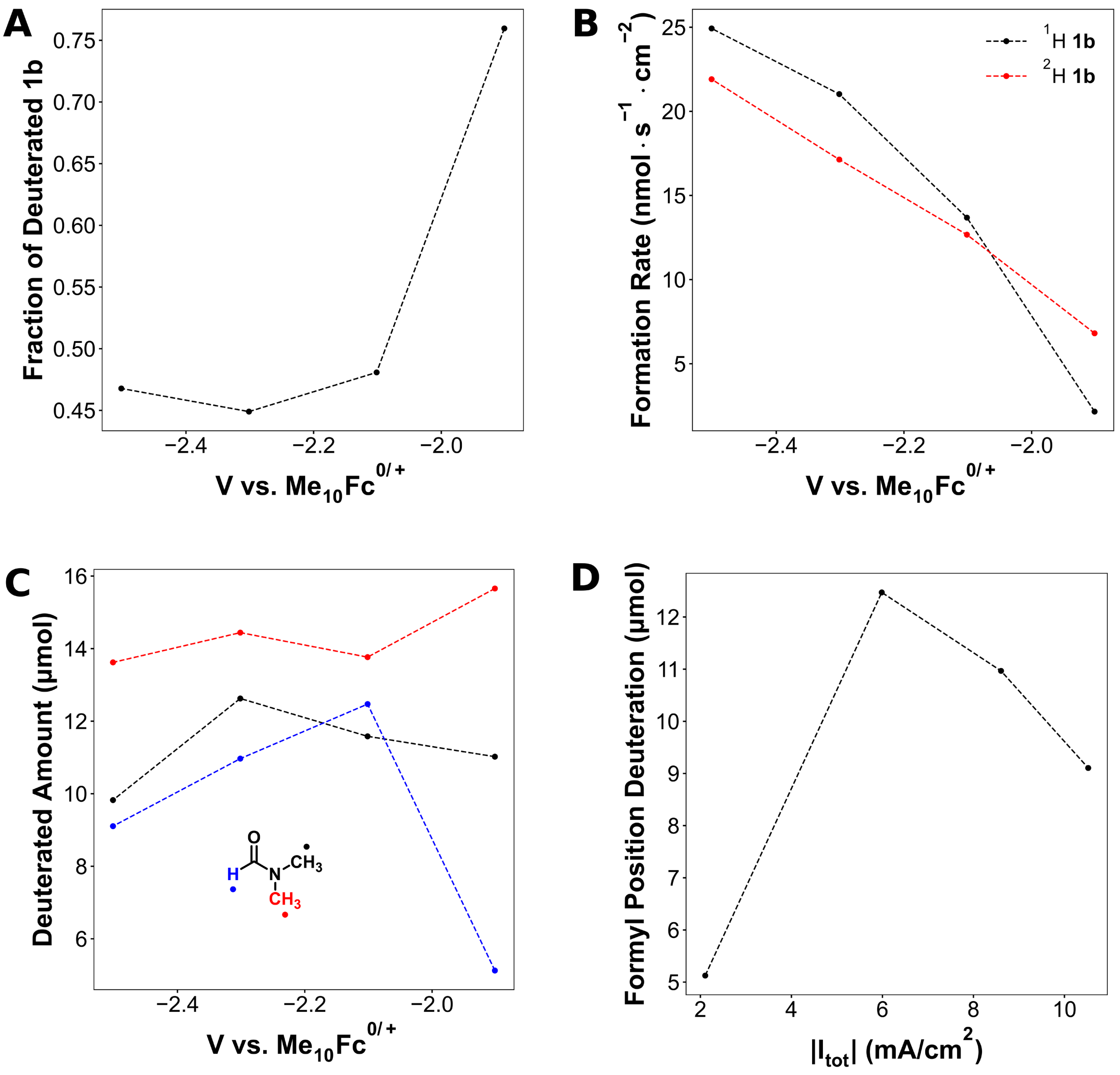

Figure S17. Reduction of 100 mM **1a** in the presence of EtOD in DMF at various potentials. (**A**) Fraction of deuterated hydrogenolysis product (**1b**) as a function of potential. (**B**) Formation rates of protonated and deuterated **1b** as a function of potential. Dashed lines are to guide the eye. Amount of deuterium incorporated into DMF as a function of (**C**) potential and (**D**) total current density. Dashed lines are to guide the eye. Reaction conditions: Ag cathode, Al anode, undivided cell, 100 mM **1a**, 90 mM TBA-BF_4, 10 mM TBA-Br, 20 sccm N_2, 2.5 mL DMF, 400 mM EtOD. Experiments were run for either 20 C or 1 hr, whichever condition was met first.

Similar trends for the fraction of deuerated **1b**, formation rates of protonated and deuterated **1b**, and amount of deuterium incorporated into DMF are seen for 100 mM **1a** as for 20 mM **1a** (**Figure S17**). A sharp change in the deuterated fraction of **1b** and amount of formyl deuterium in DMF occurs beginning around -1.9 V vs. $Me_{10}Fc$ for both initial concentrations. The formation rate of protonated **1b** also increases faster than the formation rate of deuterated **1b** beginning at around -1.9 V. To rule out the impact of current density affecting the EtOD concentration near the cathode via exchange with the solvent, the amount of formyl-postion

deuterium and the total current density are compared (**Figure S16** and **Figure S17D**). With 100 mM **1a** initially, the formyl deuterium content of DMF is around 5 μmol at 2 mA. At 20 mM **1a** initially, the formyl deuterium content in DMF is noticeably higher at 2 mA. Even though the deuterium content in DMF correlates positive with current density magnitude for a fixed initial concentration of **1a**, comparing similar current densities across initial concentrations reveals that voltage, not current density, is the governing factor determining when deuterium content in DMF increases. This finding rules out local depletion of EtOD as a reason for the observed changes in deuterium contents in **1b** and DMF at potentials more reductive than -1.9 V.

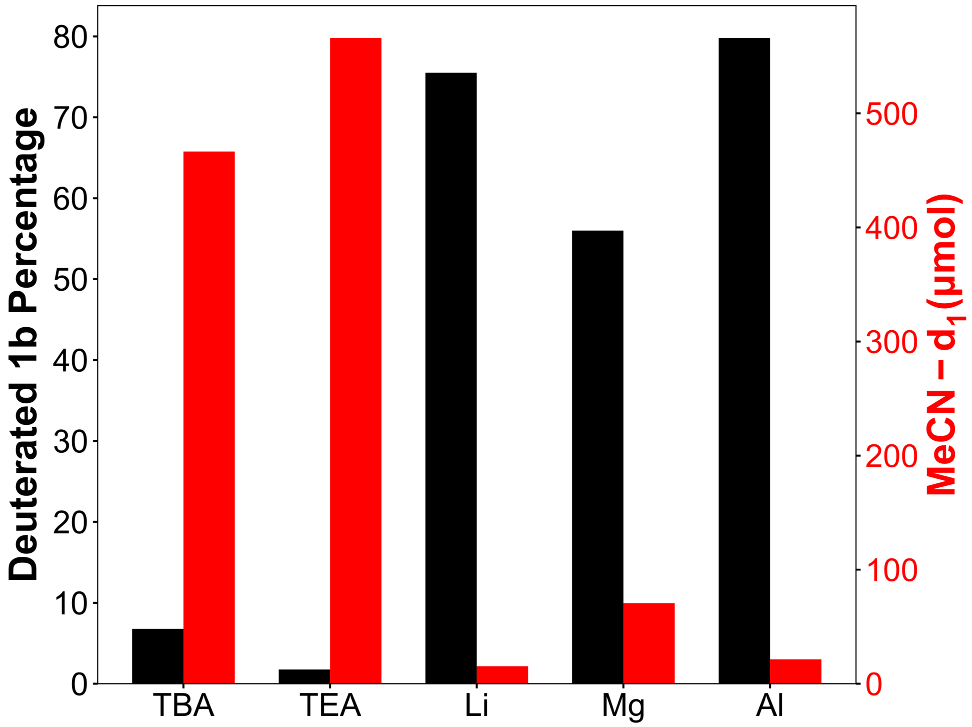

Figure S18. Percentage of deuterated **1b** (left axis) and amount of deuterium incorporated into MeCN (right axis) as a function of cation identity. Reaction conditions: Ag cathode, Al anode, undivided cell, 400 mM EtOD, 100 mM **1a**, 2.5 mL MeCN, 20 sccm N_2, -1.71 V vs. $Me_{10}Fc$. Electrolyte salts 95 mM X-BF_4, 5 mM XBr (X = TBA, TEA, or Li); 100 mM TBA-BF_4, 25 mM XBr_n (X = Mg, Al). 25 mM $AlBr_3$ was found to not be entirely soluble in the electrolyte, so it was at the saturation limit. Stopping condition: 7 C of charge or 1 hr elapsed time. For the experiment with lithium salts, the current became very low, possibly due to precipitation of Li salts on the cathode.

In MeCN, the amount and fraction of hydrogenolysis product that is deuterated after electrolysis is highly dependent on the nature of the cation in the electrolyte. Weakly coordinating cations such as TBA and TEA result in low deuteration amounts with subsequently high deuteration of MeCN. Conversely, with strongly coordinating cations, the deuterated amount of **1b** increased with a concomitant decrease in the deuteration content of MeCN. Based off these observations, the production of ethoxide by deprotonation of EtOD can cause proton/deuterium exchange reactions with MeCN as follows:

$$EtO^- + CH_3CN \rightarrow EtOH + CH_2CN^-$$

$$CH_2CN^- + EtOD \rightarrow EtO^- + CH_2DCN$$

Acetonitrile is an acidic enough solvent such that these exchange reactions proceed rapidly in the presence of weakly coordinating cations. At statistical equilibrium, about 0.7% of the initially 400 mM EtOD will remain deuterated. Near the cathode where the ethoxide concentration will be highest, the amount of EtOD will be significantly reduced, resulting in low amounts of deuterated **1b**. With strongly coordinating cations, the ethoxide loses its basicity and is unable to exchange with MeCN.

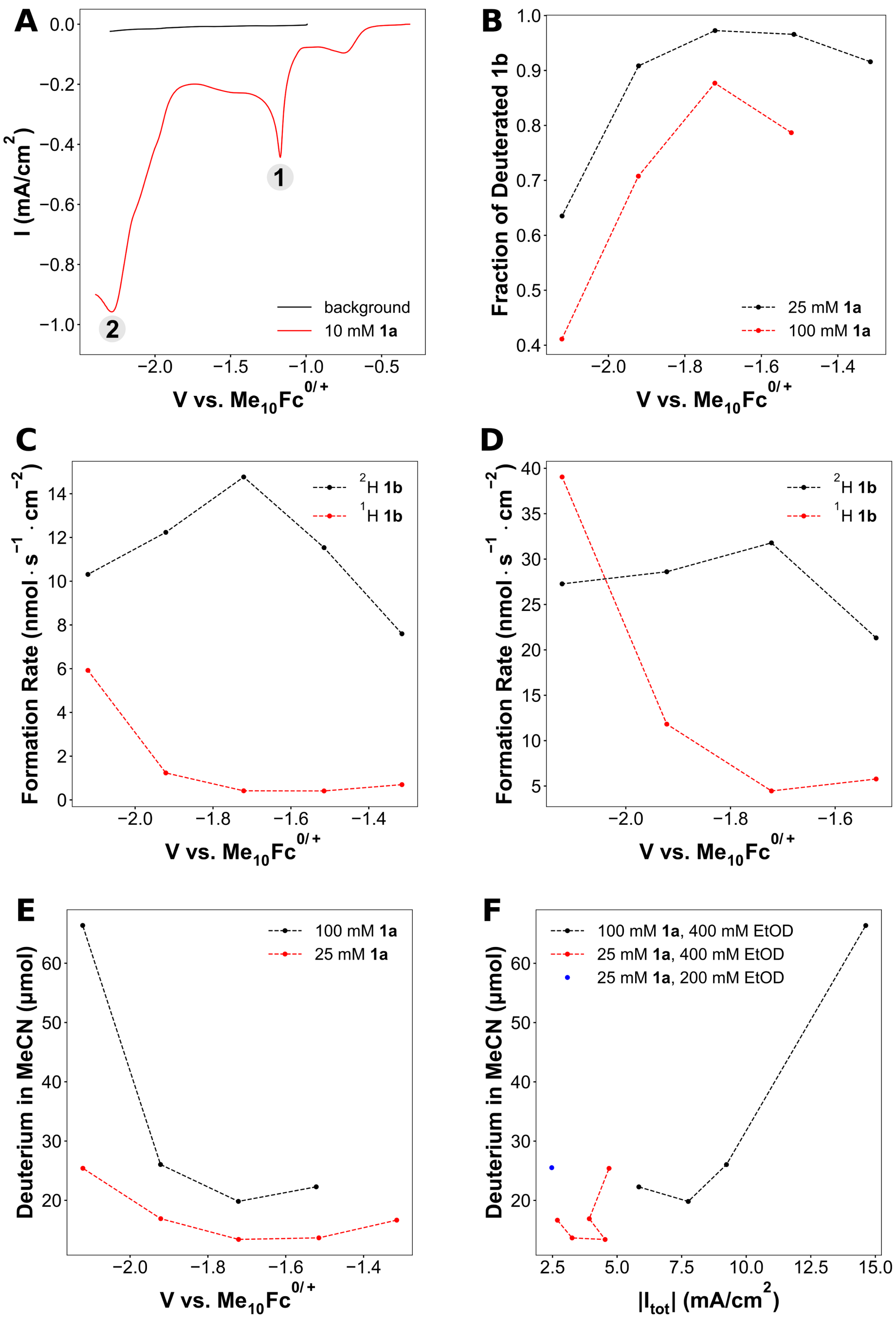

Figure S19. Reduction of **1a** in the presence of EtOD in MeCN at various potentials. (**A**) Linear sweep voltammograms (LSVs) at 10 mV/s in MeCN with and without 10 mM **1a**. Electrolyte: 92.5 mM TBA-BF_4 and 7.5 mM TBA-Br. (**B**) Fraction of deuterated hydrogenolysis product (**1b**) as a function of potential. Formation rates of protonated and deuterated **1b** as a function of potential initially with (**C**) 25 mM **1a** and (**D**) 100 mM **1a**. Amount of deuterium incorporated into MeCN as a function of (**E**) potential and (**F**) total current density. Dashed lines are to guide the eye. Reaction conditions: 25 or 100 mM **1a**, 100 mM TBA-BF_4, 25 mM $AlBr_3$, 20 sccm N_2, 2.5 mL MeCN, 400 mM EtOD. Experiments were run for either 7 C or 1 hr, whichever condition was met first.

Similar trends regarding the deuterated fraction of **1b**, formation rates of deuterated and protonated **1b**, and deuterium content in the solvent are found in MeCN as in DMF (**Figure S19**). The deuterated fraction of **1b** decreases and the amount of deuterated MeCN increases below -1.8 V, which is around the same voltage where the second major reduction wave begins in the LSV of **1a** in MeCN. These changes do not appear as sharp as they do in DMF; additionally, more reductive voltages were not probed due to the onset of direct EtOD reduction. For these experiments, $AlBr_3$ was added to the electrolyte to prevent deuterium exchange with the solvent.

To rule out current density directly leading to increased levels of deuterium in the solvent, a comparison of deuterated MeCN content across different initial concentrations of **1a** and EtOD was done (**Figure S19F**). As noted for DMF, while for a fixed set of initial reagent conditions the deuterated MeCN content correlates positively with total current density, this correlation breaks when looking across different initial reagent conditions. This data further supports that the change in deuterated **1b** and the solvent are a result of the applied potential, not current density. It is worth noting that the $AlBr_3$ may not be able to fully suppress deuterium exchange with the solvent at 100 mM **1a** since the initial deuterated MeCN levels are higher than those with 25 mM **1a**. However, the important conclusion is that the large change in deuterated MeCN content is a result of the applied potential, not the total current density and therefore not EtOD depletion near the cathode.

The presence of exchange reactions can complicate the interpretation of deuterium incorporation experiments. As noted above, in MeCN, ethoxide anions can rapidly exchange protons with MeCN, creating a chain reaction resulting in nearly all of the EtOD near the cathode being converted into EtOH. The amount of deuterated hydrogenolysis product becomes significantly lower as a result. This problem is exacerbated at higher current densities where the interfacial concentration of ethoxide becomes higher. In light of these observations, care must be taken to ensure the drop in deuterated **1b** fraction is a genuine result of solvent deprotonation and not due to a decrease in EtOD concentration from exchange reactions. By comparing data across different reaction conditions, we are able to rule out current density as a direct cause for changes in deuteration content, which supports the claim that changes in deuteration content are caused by the applied potential and the occurrence new electrochemical reactions.

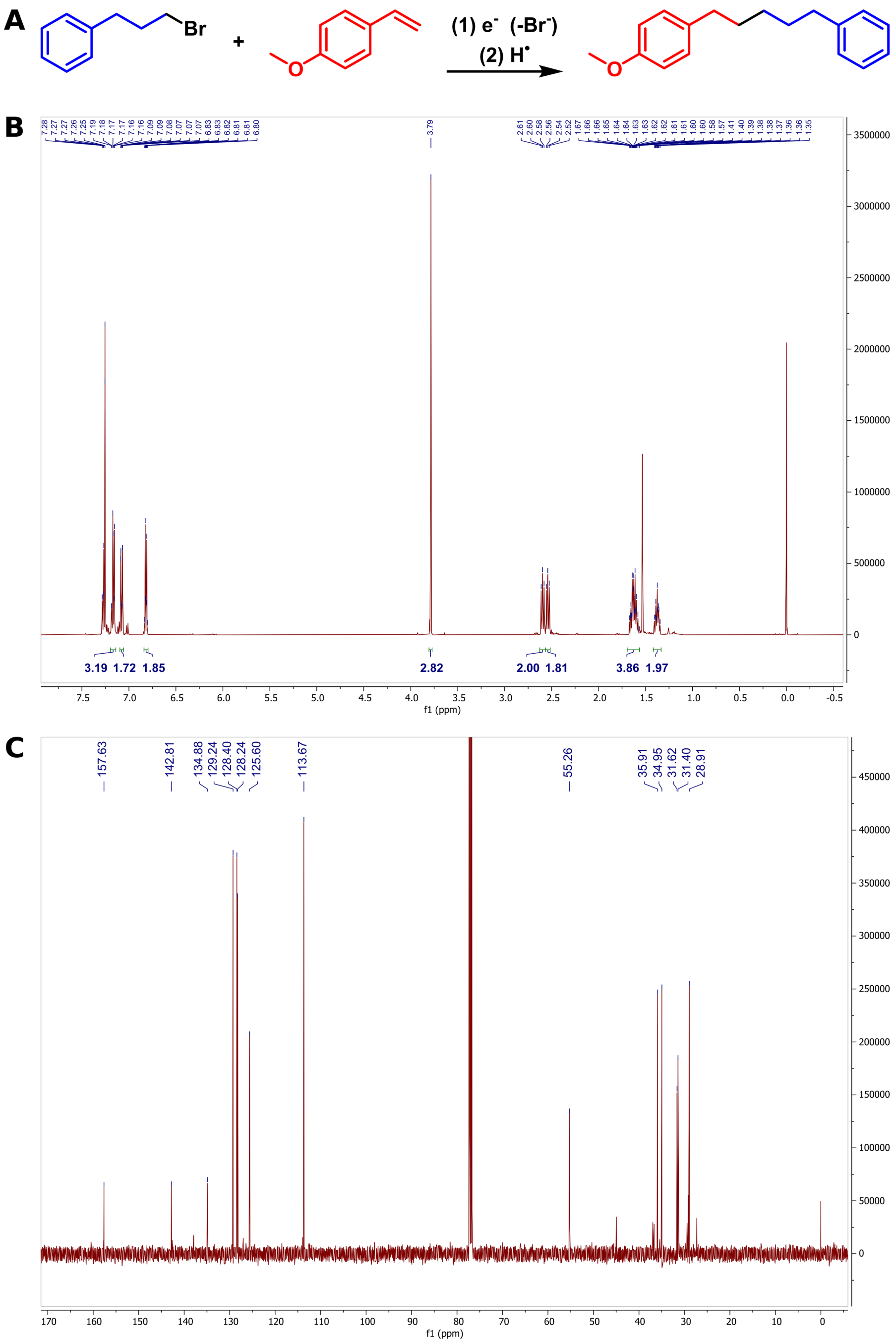

Figure S20. Confirmation of the reductive coupling between **1a** and 4-vinylanisole (4VA). (**A**) Overall reductive coupling reaction. (**B**) ^{1}H and (**C**) ^{13}C NMR spectra of the purified coupling product.

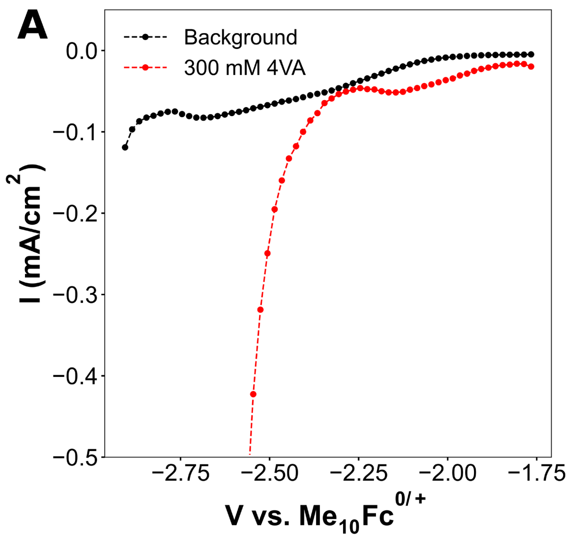

Figure S21. Staircase voltammograms (20 mV steps with 30 s hold time) of 4VA in (**A**) DMF and (**B**) MeCN. Experimental conditions: Ag cathode, Al anode, undivided cell, 2.5 mL solvent, 90 mM TBA-BF_4, 10 mM TBA-Br, 20 sccm N_2. The increase in apparent background current with 4VA may be attributed to small amounts of phenol in the commercial 4VA reagent.

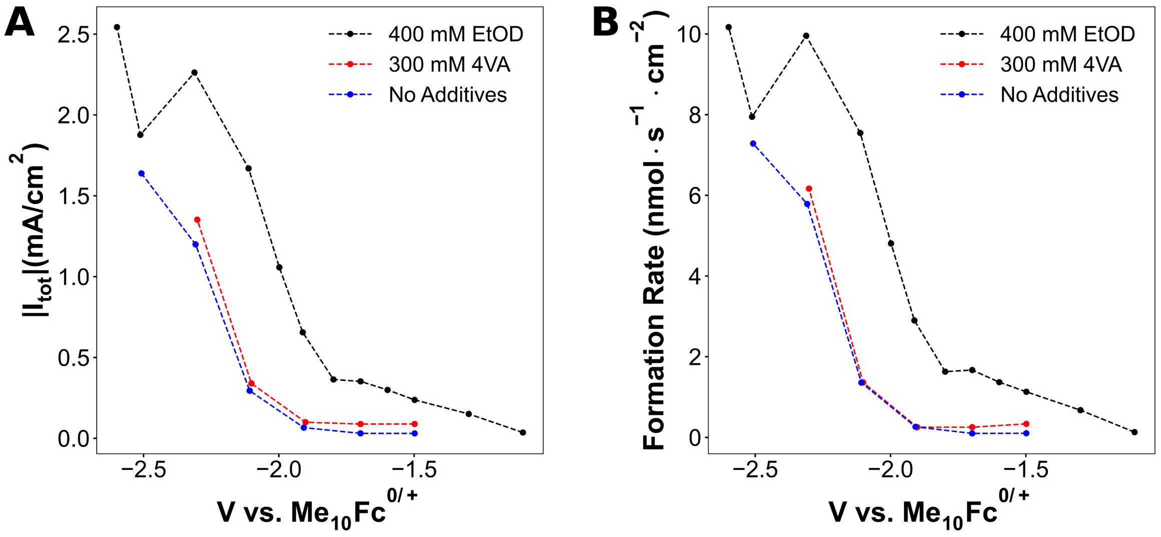

Figure S22. Reduction of **1a** with an anion trap (EtOD) and radical trap (4VA) in DMF as a function of applied potential. Impact of traps on (**A**) the total current density magnitude and (**B**) formation rate of **1b** (protonated + deuterated). Reaction conditions: Ag cathode, Al anode, undivided cell, 20 mM **1a**, 90 mM TBA-BF_4, 10 mM TBA-Br, 20 sccm N_2, 2.5 mL DMF. Experiments were run for either 4 C or 1 hr, whichever condition was met first.

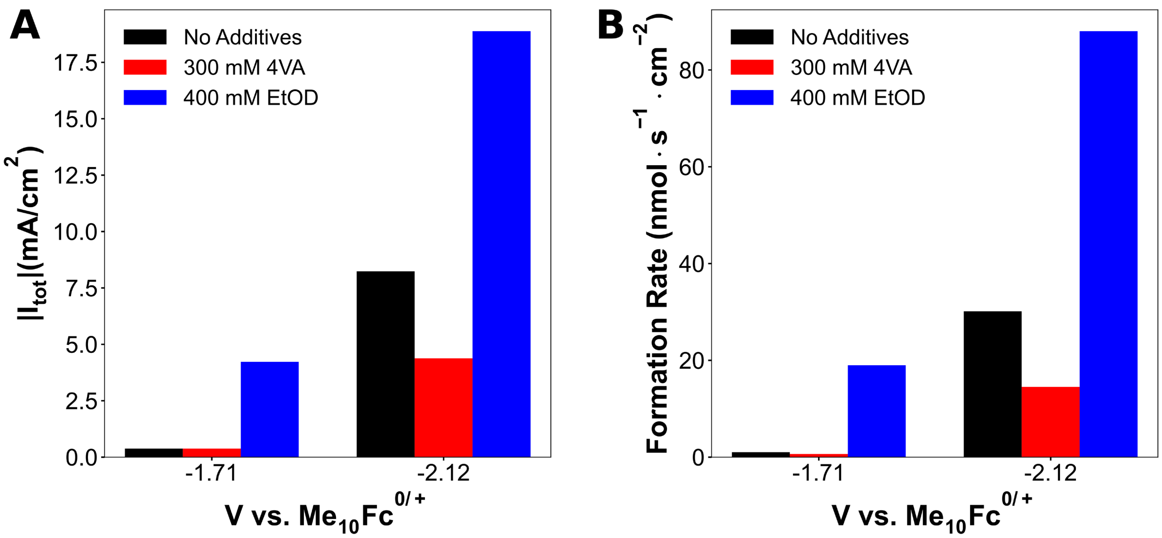

Figure S23. Reduction of **1a** with an anion trap (EtOD) and radical trap (4VA) in MeCN as a function of applied potential. Impact of traps on (**A**) the total current density magnitude and (**B**) formation rate of **1b** (protonated + deuterated). Reaction conditions: Ag cathode, Al anode, undivided cell, 100 mM **1a**, 90 mM TBA-BF_4, 10 mM TBA-Br, 20 sccm N_2, 2.5 mL MeCN. Experiments were run for either 7 C or 1 hr, whichever condition was met first.

To test for radical intermediates, 4-vinylanisole (4VA) was added to trap radicals but not anions. At potentials where the reduction of **1a** occurs but the reduction of 4VA does not, a coupling product between 4VA and **1a** was observed (**Figure S20** and **Figure S21**). The coupling product proves radical intermediates are formed after the initial reduction of **1a**, although many of these radicals may be adsorbed to the silver electrode. The addition of 300 mM 4VA for the reduction of **1a** increases the total current density slightly and barely affects the formation rate of **1b** (**Figure S22**). In comparison, EtOD increases the formation rate of **1b** and the total current density more significantly. Similar trends regarding the additions of EtOD and 4VA are seen in MeCN as in DMF (**Figure S23**). 4VA has an almost negligible impact on the total current density while EtOD increases it significantly. At -2.12 V, 4VA did reduce the amount of **1b** that was formed relative to without additives. Coupled with the nearly unchanged total current density, this result suggests that it can intercept intermediates that lead to **1b**, although the magnitude may be solvent dependent. However, unlike EtOD, 4VA does not result in a noticeable increase in the reduction rate of **1a**, so it is likely only intercepting already-formed radical intermediates and not inducing new electrochemical reactions.

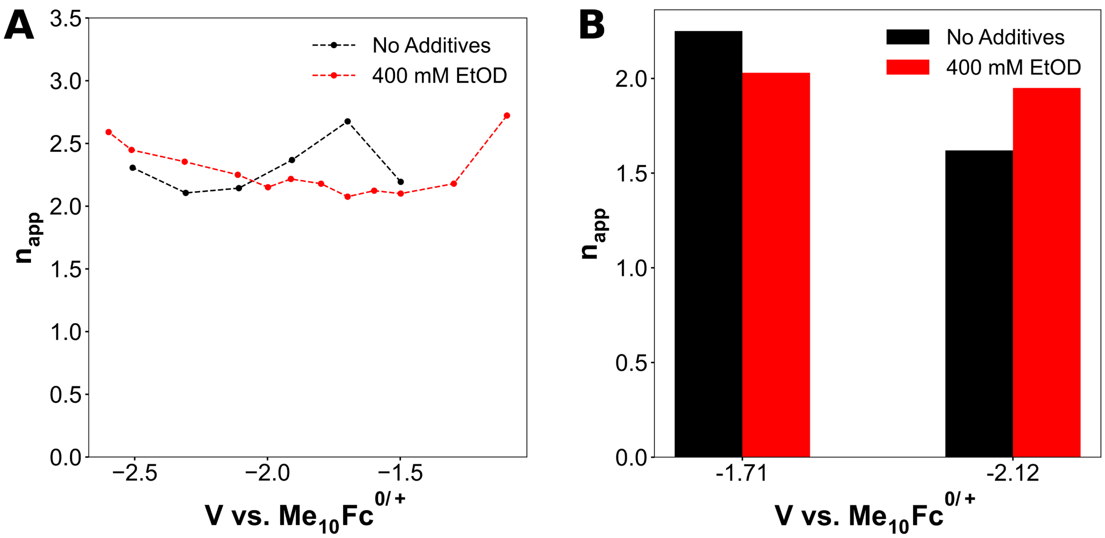

Figure S24. Apparent number of electrons transferred (n_{app}) for the reduction of (**A**) 20 mM **1a** in DMF and (**B**) 100 mM **1a** in MeCN as a function of potential. The value of n_{app} was calculated by dividing the total charge passed during the experiment by the moles of **1a** converted to **1b** and **1d**. Reaction conditions for **A**: Ag cathode, Al anode, undivided cell, 20 mM **1a**, 90 mM TBA-BF_4, 10 mM TBA-Br, 20 sccm N_2, 2.5 mL DMF. Experiments were run for either 4 C or 1 hr, whichever condition was met first. Reaction conditions for **B**: Ag cathode, Al anode, undivided cell, 100 mM **1a**, 90 mM TBA-BF_4, 10 mM TBA-Br, 20 sccm N_2, 2.5 mL MeCN. Experiments were run for either 7 C or 1 hr, whichever condition was met first.

For total current densities above 0.1 mA/cm^2 in magnitude, the apparent number of electrons is similar with and without EtOD. When the total current density drops below 0.1 mA/cm^2 in magnitude, n_{app} begins to increase due to the greater relative importance of background reductive processes on the total current, e.g. trace O_2 reduction. The use of n_{app} to make mechanistic judgments can be complicated by the reduction of trace impurities (more relevant at low current densities) and the reduction of byproducts of electrochemical reactions. As an example, the reduction of **1a** may involve just 1e$^-$ at potentials more anodic of -2 V, but the byproducts of hydrogen abstraction could be further reduced by 1e$^-$. The overall process would appear as 2e$^-$ per molecule of **1a**, but only 1e$^-$ is transferred to **1a**. We resorted to more detailed isotopic and product quantification experiments to more rigorously identify the mechanistic pathways of hydrogenolysis.

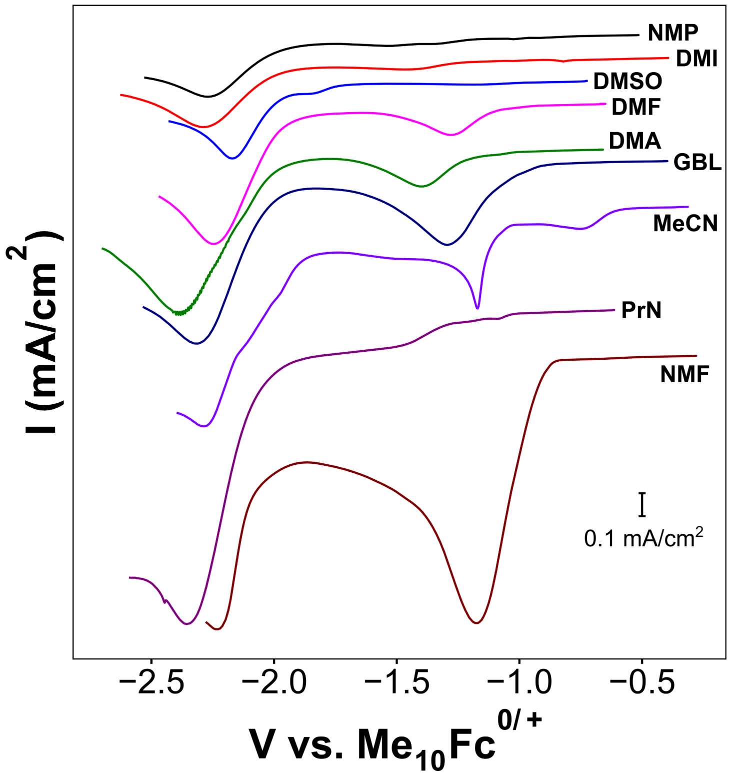

Figure S25. Linear sweep voltammograms of **1a** in various solvents. Experimental conditions: Ag cathode, Al anode, undivided cell, 10 mM **1a**, 90 mM TBA-BF$_4$, 10 mM TBA-Br, 2.5 mL solvent. LSVs began at open circuit voltage with a sweep rate of 10 mV/s.

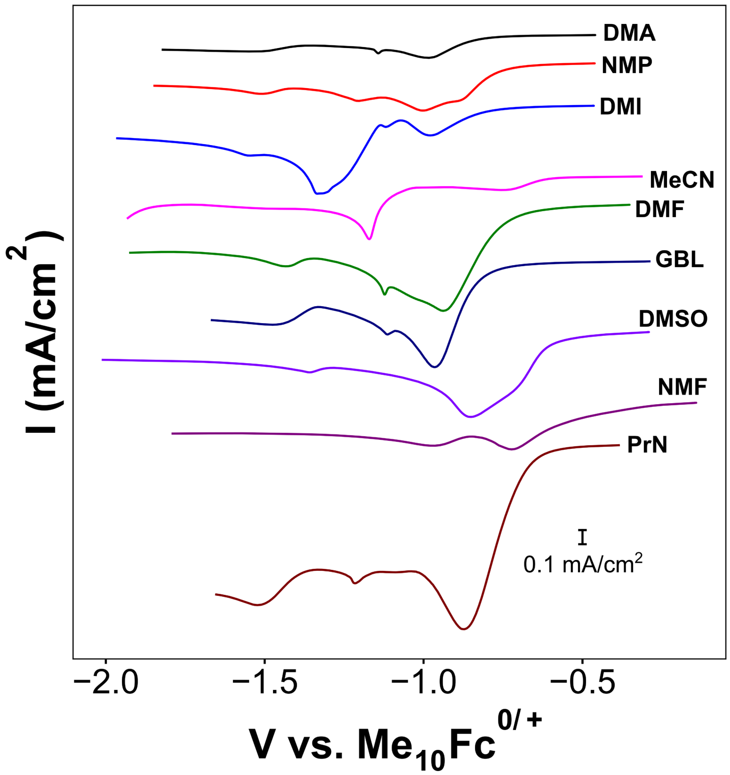

Figure S26. Linear sweep voltammograms of (1-bromoethyl)benzene (**2a**) in various solvents. Experimental conditions: Ag cathode, Al anode, undivided cell, 10 mM **2a**, 90 mM TBA-BF_4, 10 mM TBA-Br, 2.5 mL solvent. LSVs began at open circuit voltage with a sweep rate of 10 mV/s.

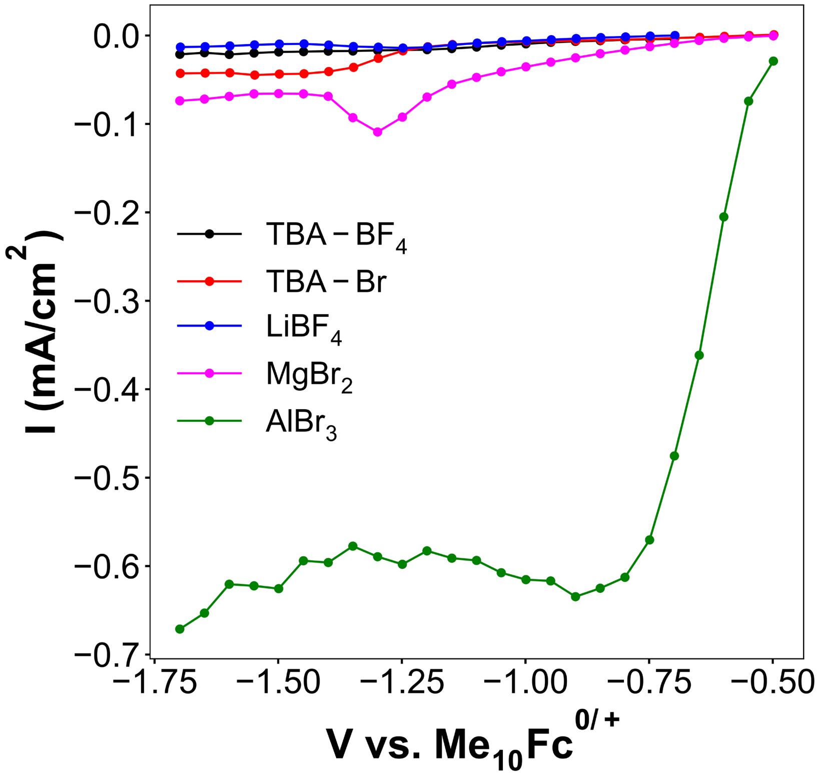

Figure S27. Staircase voltammograms (50 mV steps with 30 s hold time) of various background electrolytes in MeCN. Experimental conditions: Ag cathode, Al anode, undivided cell, 2.5 mL solvent, 20 sccm N_2. Electrolytes: 100 mM **TBA-BF_4**, 100 mM **TBA-Br**, 95 mM TBA-BF_4 + 5 mM TBA-Br + 25 mM **$LiBF_4$**, 100 mM TBA-BF_4 + 25 mM **$MgBr_2$**, 100 mM TBA-BF_4 + 25 mM **$AlBr_3$**. Bolded compounds correspond to the entries in the legend.

The addition of $MgBr_2$ and $AlBr_3$ resulted in noticeably increases in background current. We hypothesize that this increase in background current is due to water absorbed by these very hygroscopic salts. Magnesium and aluminum cations are also Lewis acidic, so water coordinated to these cations may have enhanced acidity and reduce at less negative potentials, resulting in higher apparent background currents. Similar behavior has been seen for the reduction of water in aprotic electrolytes with alkali metal cations.[30] We avoided using these salts for our experiments to eliminate the confounding effects this background current could have. For carboxylation experiments, the lack of initial inorganic halide salts in the electrolyte does lead to the formation of some ester product. The ester product was added to the carboxylic acid product to obtain the total amount of carboxylated product, as the ester is obtained from a non-electrochemical nucleophilic reaction of the carboxylate product in the electrolyte with the organic halide substrate.[7]

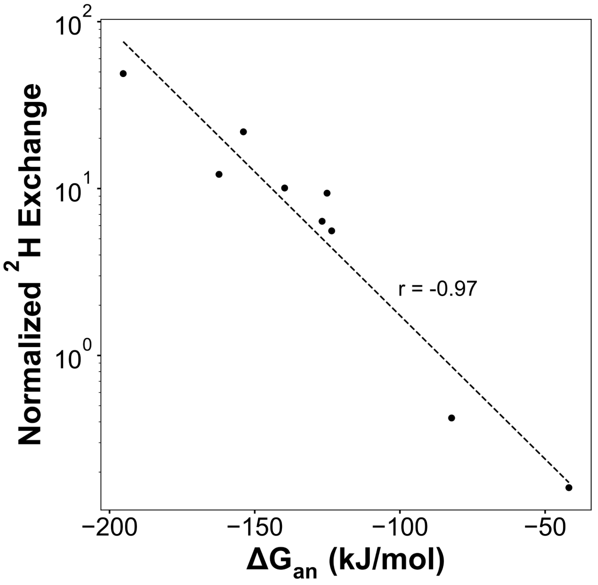

Figure S28. Correlation between *ΔG*$_{an}$ and the normalized amount of deuterium exchange at the most acidic position in each solvent. Experimental conditions: Ag cathode, Al anode, undivided cell, 400 mM EtOD, 20 sccm N_2, -5 mA/cm^2 for 30 min. The Pearson correlation coefficient is given as *r*.

The ability of DFT to predict reasonable deprotonation free energies was validated by comparing the normalized solvent-EtOD deuterium exchange from experiments described in Section 3.3. to the computed deprotonation free energies for the most acidic position on each solvent (**Figure S28**). The normalized deuterium exchange was calculated as the total amount of additional deuterium (subtracting off the naturally occurring amount) in the solvent at the most acidic position (μmol) divided by the concentration of hydrogen atoms at that position (mol/L). The concentration of hydrogen atoms was found by multiply the concentration of the molar concentration of the solvent by the number of equivalent hydrogen atoms at the most acidic position. This normalization accounts for the concentration of acidic centers on each solvent molecule. Since each experiment was run for the same amount of time, this normalized deuterium exchange is proportional to the turnover frequency per acidic site. The turnover frequency is exponentially proportional to the free energy of activation.

A plot of *ΔG*$_{an}$ versus the logarithm of the normalized ^{2}H exchange shows a fairly linear trend with a negative slope. This strong correlation confirms that the *ΔG*$_{an}$ is representative of kinetic solvent acidity and validates its use as a computational descriptor. The most acidic position on each solvent model determined by DFT is also in agreement with the position that had the most deuterium exchange. Additionally, negligible deuterium exchange was seen for the less acidic sites on each molecule (except NMF, where some exchange was also seen at the formyl position), in agreement with DFT which predicts *ΔG*$_{an}$ > -51 kJ/mol; noticeable deuterium exchange was only noticed on sites with *ΔG*$_{an}$ < -90 kJ/mol. The consistency between the deuterium-exchange acidity measurements and computationally derived *ΔG*$_{an}$ validates the computational methodology used in this work to assess the acidity of solvents.

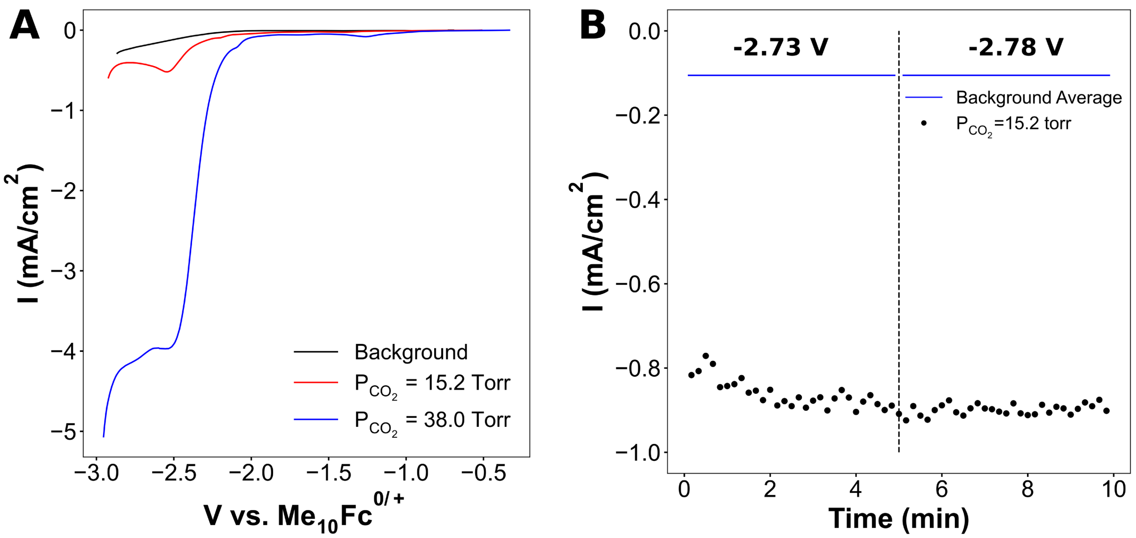

Figure S29. Analysis of CO_2 mass transport. (**A**) LSV's of CO_2 at reduced pressures to enable visualization of mass transport limitations. (**B**) Evaluation of the transport limited CO_2 current at 15.2 Torr under the same hydrodynamic conditions as carboxylation experiments. Two potentials (V vs. $Me_{10}Fc$) were tested to ensure the invariance current density, indicating the current density is mass transport limited. Conditions: Ag cathode, Pt anode, divided cell w/ Daramic separator, 20 sccm total gas flow (CO_2 + N_2), 0.1 M TBA-BF_4 (both compartments), 2.5 mL DMF (both compartments).

The transport limited current density of CO_2 in DMF under typical hydrodynamic conditions was evaluated at reduced CO_2 pressure to obtain current densities (**Figure S29**). At 15.2 Torr CO_2 (2% of atmospheric pressure), the transport limited current density is between -0.8 and -0.9 mA/cm^2 at 20 sccm total gas flow as averaged from the data at -2.78 V vs. $Me_{10}Fc$. The background current at these potentials is -0.1 mA/cm^2. We report the transport limited current density as a range to indicate uncertainty in the nature of this background current. We can rule out O_2 impurities as a primary contributor to this current since the background current density at -2 V, a potential where O_2 reduction would be mass transport limited, is less than -10 μA/cm^2. Since the transport limited current should be linear in CO_2 pressure under dilute conditions (valid here since the solubility of CO_2 in DMF is 199 mM at 25 °C)[31]. At 1 atm of pressure, the transport limited current is between -40 and -45 mA/cm^2 in our system with 20 sccm of bubbling. CO_2 reduction in aprotic solvents consumes one molecule of CO_2 per electron transferred:[32]

$$2CO_2 + 2e^- \rightarrow CO + CO_3^{2-}$$

At the most reductive potential studied for carboxylation in DMF (-2.41 V vs. $Me_{10}Fc$), the current density of CO_2 reduction to CO is estimated to be -10 mA/cm^2 assuming the Faradaic efficiency (FE) not accounted for by the reduction of **1a** goes toward CO (**Table S4**). This assumption is justified by FE closures at constant current which indicated CO accounts for the remaining current not going toward the reduction of **1a** (**Table S6** and **Table S7**). CO_2 is also consumed by the carboxylation reaction, which has a current density of -2.14 mA/cm^2 at -2.41 V vs. $Me_{10}Fc$. Accounting for all CO_2 consuming reactions at the cathode, the flux of CO_2 is 25–28% of its transport limited value. With a stagnant boundary layer assumption, the interfacial

concentration of CO_2 is 72–75% of the bulk concentration. The effect of this concentration drop on the rate of carboxylation relative to the rate of hydrogenolysis will depend on the CO_2 order dependence of carboxylation. Since carboxylation involves one molecule of CO_2, the CO_2 order dependence is likely between zero and one. The carboxylation rate would be 72–100% of its value in the absence of CO_2 mass transport limitations. Therefore, modest transport effects are in play at -2.41 V, but these effects are likely not enough to meaningfully alter correlations with descriptors. Analysis for CO_2 transport limitations in MeCN and DMSO revealed similar results, suggesting interfacial CO_2 concentrations at the most reductive potentials of 75% (MeCN) and 67% (DMSO) of their bulk values. At less cathodic potentials, CO_2 mass transport would have a smaller effect. The carboxylation of **2a** is much less impacted by CO_2 mass transport since it occurs at less cathodic potentials where direct CO_2 reduction is much less prevalent.

Table S3. Kinetic isotope effect data for the carboxylation of **1a** and **2a** in MeCN.

**Reaction Conditions**	**Carboxylation rate ($nmol \cdot s^{-1} \cdot cm^{-2}$)**	**Hydrogenolysis rate ($nmol \cdot s^{-1} \cdot cm^{-2}$)**	**CHR**
MeCN, 20 mM **1a**, -2.28 V	10.1	15.1	0.67
MeCN-d_3, 20 mM **1a**, -2.20 V	10.3	11.2	0.92
MeCN, 20 mM **1a**, -2.33 V	9.31	16.7	0.56
MeCN-d_3, 20 mM **1a**, -2.36 V	9.67	12.5	0.77
MeCN, 20 mM **2a**, -1.75 V	39.4	0.93	42.1
MeCN-d_3, 20 mM **2a**, -1.75 V	31.9	1.24	25.7

All potential are reference to $Me_{10}Fc^{0/+}$

Reaction conditions: See **Table S4** for **1a** and **Table S5** for **2a**.

Carboxylation was performed in protonated and deuterated MeCN to assess whether a kinetic isotope effect existed for either product. For the carboxylation of **1a** at two potentials (2nd cathodic peak potential and -160 mV of the 2nd cathodic peak potential), the carboxylation rates are comparable, but the hydrogenolysis rate lowers when using MeCN-d_3. This result is consistent with the hydrogenolysis rate depending on deprotonating MeCN, which becomes slow when MeCN is deuterated. For the carboxylation of **2a**, this effect is not seen. The hydrogenolysis rate appears higher in MeCN-d_3, but the fraction of deuterated **2b** is less than unity. Other sources of hydrogen atoms become relevant at the low hydrogenolysis rates seen during the carboxylation of **2a**.

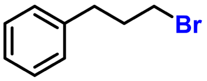

1a

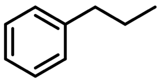

1b

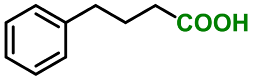

1c

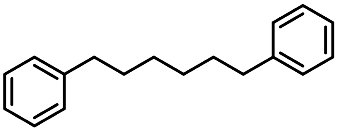

1d

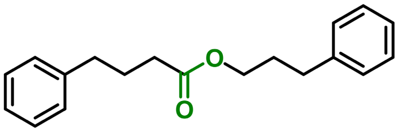

1e

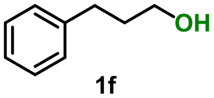

1f

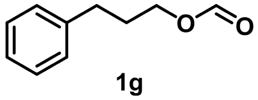

1g

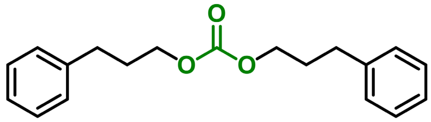

1h

Table S4. Electrical and product distribution data for the carboxylation of **1a** at constant potential.

Solvent	E_{app}	I_{avg}	Q		1a	1b	1c	1e	1d	1f	MB	CHR
DMA	-2.37 (+13 mV)	4.97	10	**N**	25.6	11.1	9.2	0.66	6.6×10^{-3}	0.24	97.6	0.89
				**Yield**	52.7	22.8	19.0	2.7	0.027	0.50		
				**FE**		21.4	17.8	1.3	0.013			
	-2.52 (-143 mV)	9.33	16	**N**	29.6	7.0	8.7	0.79	3.9×10^{-3}	1.2	90.0	1.37
				**Yield**	55.3	13.1	16.2	2.9	0.015	2.2		
				**FE**		8.4	10.5	0.9	4.7×10^{-3}			
	-2.33	3.77	9	**N**	25.4	11.3	8.7	0.68	N.D.	0.25	95.4	0.83
				**Yield**	51.7	23.1	17.8	2.8		0.51		
				**FE**		24.2	18.7	1.5				

Solvent	E_{app}	I_{avg}	Q		1a	1b	1c	1e	1d	1f	1g	MB	CHR
DMF	-1.91	0.12	0.44	**N**	41.0	0.26	2.16	0.093	0.046	N.D.	N.D.	89.8	8.52
				**Yield**	84.2	0.54	4.4	0.38	0.19				
				**FE**		11.7	95.2	4.1	2.0				
	-2.01	0.10	0.36	**N**	37.4	0.18	1.59	0.047	0.012	N.D.	N.D.	75.2	9.35
				**Yield**	71.6	0.33	3.0	0.18	0.05				
				**FE**		9.3	84.5	2.5	0.7				
	-2.11	0.15	0.52	**N**	40.6	0.40	1.99	0.075	0.009	N.D.	N.D.	88.6	5.14
				**Yield**	83.3	0.82	4.1	0.31	0.04				
				**FE**		14.7	73.0	2.8	0.3				
	-2.21 (+3 mV)	1.58	4	**N**	36.2	3.5	8.7	0.19	N.D.	N.D.	0.41	91.7	2.52
				**Yield**	67.5	6.6	16.2	0.70			0.76		
				**FE**		17.0	41.8	0.9					
	-2.41 (-168 mV)	12.9	8	**N**	35.5	2.3	6.6	0.31	N.D.	N.D.	0.26	93.2	2.98
				**Yield**	73.0	4.8	13.6	1.3			0.54		
				**FE**		5.6	15.9	0.7					
	-2.31	7.25	6	**N**	34.1	3.1	7.3	0.48	N.D.	N.D.	0.42	94.6	2.51
				**Yield**	70.3	6.4	15.1	2.0			0.88		
				**FE**		10.0	23.5	2.0					
	-2.51	14.2	12	**N**	33.3	2.11	6.64	0.33	0.005	N.D.	0.18	88.5	3.31
				**Yield**	68.7	4.3	13.7	1.3	0.02		0.37		
				**FE**		3.4	10.7	0.5	8×10^{-3}				

Solvent	E_{app}	I_{avg}	Q		1a	1b	1c	1e	1d	1f	MB	CHR
				**N**	31.8	4.8	9.2	1.5		0.99		
	-2.34 (-48 mV)	2.13	6	**Yield**	62.4	9.5	17.9	5.9	N.D.	1.9	100.9	2.38
				**FE**		15.5	17.9	5.9				
				**N**	31.1	2.2	4.7	0.78		3.5		
DMI	-2.48 (-188 mV)	5.14	10	**Yield**	62.3	4.4	9.5	3.1	N.D.	7.1	88.4	2.70
				**FE**		4.2	9.5	3.1				
				**N**	28.4	6.1	3.3	4.7		1.8		
	-2.30	1.50	5	**Yield**	57.0	12.3	6.6	18.7	N.D.	3.6	102.2	1.50
				**FE**		23.7	12.7	18.0				
**Solvent**	**E_{app}**	**I_{avg}**	**Q**		**1a**	**1b**	**1c**	**1e**	**1d**	**1f**	**MB**	**CHR**
				**N**	37.0	4.2	3.6	0.54				
	-2.22 (-53 mV)	0.89	3.2	**Yield**	76.4	8.6	7.4	2.2	N.D.	N.D.	95.1	0.99
				**FE**		25.1	21.6	3.3				
				**N**	35.6	6.2	6.0	0.11		0.18		
DMSO	-2.32 (-145 mV)	4.28	8	**Yield**	71.2	12.4	11.9	0.44	N.D.	0.36	96.8	0.98
				**FE**		15.0	14.4	0.27				
				**N**	27.3	6.5	3.7	3.0		1.2		
	-2.31	2.4	8	**Yield**	56.2	13.4	7.7	12.2	N.D.	2.4	92.6	1.03
				**FE**		15.7	9.0	7.2				
**Solvent**	**E_{app}**	**I_{avg}**	**Q**		**1a**	**1b**	**1c**	**1e**	**1d**	**1f**	**MB**	**CHR**
				**N**	34.7	8.1	6.6	0.12		0.057		
	-2.30 (+13 mV)	3.97	8	**Yield**	67.4	15.8	12.8	0.45	N.D.	0.11	96.6	0.83
				**FE**		19.6	15.9	0.3				
				**N**	35.2	6.7	6.2	0.088		0.083		
GBL	-2.46 (-144 mV)	7.30	12	**Yield**	69.7	13.3	12.4	0.35	N.D.	0.16	95.8	0.95
				**FE**		10.8	10.0	0.14				
				**N**	32.7	8.9	7.3	0.12		0.045		
	-2.29	3.74	8	**Yield**	65.2	17.8	14.5	0.49	N.D.	0.089	98.0	0.83
				**FE**		21.6	17.5	0.29				
**Solvent**	**E_{app}**	**I_{avg}**	**Q**		**1a**	**1b**	**1c**	**1e**	**1d**	**1f**	**MB**	**CHR**
				**N**	30.7	10.3	6.9		0.068			
	-2.28 (+6.8 mV)	14.6	10	**Yield**	62.4	20.9	14.0	N.D.	0.28	N.D.	97.7	0.67
MeCN				**FE**		19.9	13.3					
				**N**	26.0	12.9	6.4	0.76	0.029	0.18		
	-2.33 (-36 mV)	25.9	20	**Yield**	53.0	26.2	13.1	3.1	0.12	0.37	95.4	0.56
				**FE**		12.4	6.2	0.73	0.028			
**Solvent**	**E_{app}**	**I_{avg}**	**Q**		**1a**	**1b**	**1c**	**1e**	**1d**	**1f**	**MB**	**CHR**
				**N**	23.8	12.4	11.4	0.042	0.27			
	-2.20 (+ 88 mV)	7.22	8	**Yield**	46.2	24.0	22.1	0.16	1.0	N.D.	93.7	0.92
MeCN-d_3				**FE**		29.8	27.4	0.10	0.64			
				**N**	19.4	19.5	14.3	0.050	0.13			
	-2.36 (-72 mV)	13.5	20	**Yield**	34.9	33.2	25.6	0.18	0.46	N.D.	95.3	0.77
				**FE**		17.9	13.8	0.048	0.12			

Solvent	E_{app}	I_{avg}	Q		1a	1b	1c	1e	1d	1f	MB	CHR
				N	28.5	18.6	0.054			0.11		
	-1.17 (+0.3 mV)	1.03	4	Yield	57.8	37.7	0.11	N.D.	N.D.	0.22	96.1	2.91×10^{-3}
				FE		89.6	0.26					
				N	29.9	18.1	0.010			0.10		
NMF	-1.33 (-157 mV)	1.24	4	Yield	58.9	35.7	0.020	N.D.	N.D.	0.20	95.1	1.24×10^{-3}
				FE		87.3	0.049					
				N	39.5	9.4	0.017			0.087		
	-2.17	35.8	20	Yield	79.1	18.8	0.034	N.D.	N.D.	0.18	98.4	1.98×10^{-3}
				FE		9.0	0.017					

Solvent	E_{app}	I_{avg}	Q		1a	1b	1c	1e	1d	1f	1h	MB	CHR
				N	40.6	3.4	2.9	0.47		0.17			
	-2.28 (-9.0 mV)	0.51	1.85	Yield	82.4	6.9	6.0	1.9	N.D.	0.35	N.D.	97.5	1.01
				FE		35.3	30.7	5.0					
				N	29.2	5.1	2.6	2.9		1.5	0.088		
NMP	-2.42 (-151 mV)	3.92	8	Yield	55.2	9.6	4.9	10.8	N.D.	2.8	0.33	83.5	1.07
				FE		12.3	6.3	6.9					
				N	33.7	6.6	5.4	0.77		0.24			
	-2.30	1.74	4	Yield	68.3	13.4	10.9	3.1	N.D.	0.48	N.D.	95.7	0.93
				FE		31.8	25.9	3.7					

Solvent	E_{app}	I_{avg}	Q		1a	1b	1c	1e	1d	1f	MB	CHR
				N	28.1	12.3	7.3	0.72		0.072		
	-2.43 (-72 mV)	18.3	15	Yield	52.9	23.2	13.8	2.7	N.D	0.14	92.8	0.65
				FE		15.8	9.4	0.93				
				N	14.7	14.9	5.2	4.5		0.25		
PrN	-2.50 (-150 mV)	17.3	20	Yield	30.3	30.7	10.8	18.7	N.D.	0.52	90.4	0.65
				FE		14.4	5.0	4.4				
				N	22.5	12.2	5.7	1.1		0.078		
	-2.31	10.1	12	Yield	47.0	25.6	11.9	4.7	N.D.	0.16	89.1	0.56
				FE		19.7	9.2	1.8				

E_{app} (V vs. $Me_{10}Fc$): Average applied potential after IR correction. For each solvent, potentials are listed for the experiment type from top to bottom: 2nd cathodic peak potential, -160 mV vs. 2nd cathodic peak potential, -2.3 V vs. $Me_{10}Fc$. For the first two, the actual potential is given relative to the 2nd cathode peak in parentheses below. Note that the actual applied potentials may deviate from the target potential due to incomplete IR compensation.

I_{avg} (mA/cm^2): Magnitude of the average current density, defined as $I_{avg} = \frac{Q}{t}$, where Q is the total charge passed and t is the time of the experiment.

Q (C): Magnitude of the total charge passed.

N (µmol): Amount of compound measured at the end of the experiment. N.D. = not detected.

Yield (%): Percentage of the initial substrate ending up in each compound, defined as $\frac{m_i N_i}{N_0} \times 100\%$, where N_i is the moles of species *i* at the end of the experiment, m_i is the number of molecules of **1a** needed to form species *i*, and N_0 is the initial moles of **1a**. For **1d**, **1e**, and the carbonate, $m_i = 2$.

FE (%): Percentage of the total passed charge going toward a given product. FE is only reported for species produced directly by Faradaic reactions. The formation of product **1b** was assumed to involve two electrons, either directly or indirectly.

MB (%): Total mass balance closure, calculated by summing up the individual yields of all detected species.

CHR: Carboxylation-to-hydrogenolysis ratio, calculated by taking the ratio of the moles of carboxylate products (**1c** and **1e**) to the hydrogenolysis product (**1b**).

N.D. Not Detected

Table S5. Electrical and product distribution data for the carboxylation of **2a** in various solvents.

Solvent	E_{app}	I_{avg}	Q		2b	2c	2e	CHR
DMA	-1.80<br>(-269 mV)	2.72	4	**N**	0.18	16.0	0.46	93.5
				**Yield**	0.35	32.0	1.8	
				**FE**	0.85	77.2	2.2	
**Solvent**	E_{app}	I_{avg}	**Q**		**2b**	**2c**	**2e**	**CHR**
DMF	-1.71<br>(-280 mV)	2.49	4	**N**	0.47	19.2	N.D.	40.9
				**Yield**	0.94	38.4		
				**FE**	2.3	92.7		
**Solvent**	E_{app}	I_{avg}	**Q**		**2b**	**2c**	**2e**	**CHR**
DMI	-1.71<br>(-279 mV)	1.78	4	**N**	0.76	17.9	0.43	24.3
				**Yield**	1.5	35.9	1.7	
				**FE**	3.6	86.5	2.1	
**Solvent**	E_{app}	I_{avg}	**Q**		**2b**	**2c**	**2e**	**CHR**
DMSO	-1.64<br>(-275 mV)	0.41	4	**N**	0.24	15.6	0.070	64.5
				**Yield**	0.49	31.3	0.28	
				**FE**	1.2	75.4	0.34	
**Solvent**	E_{app}	I_{avg}	**Q**		**2b**	**2c**	**2e**	**CHR**
GBL	-1.74<br>(-262 mV)	1.96	4	**N**	0.54	19.3	0.25	36.4
				**Yield**	1.1	38.5	1.0	
				**FE**	2.6	93.0	1.2	
**Solvent**	E_{app}	I_{avg}	**Q**		**2b**	**2c**	**2e**	**CHR**
MeCN	-1.75<br>(-257 mV)	8.21	4	**N**	0.46	19.2	N.D.	42.1
				**Yield**	0.91	38.4		
				**FE**	2.2	92.5		
**Solvent**	E_{app}	I_{avg}	**Q**		**2b**	**2c**	**2e**	**CHR**
MeCN-d_3	-1.75<br>(-259 mV)	6.91	5	**N**	0.90	23.1	N.D.	25.7
				**Yield**	1.8	46.1		
				**FE**	3.5	89.0		
**Solvent**	E_{app}	I_{avg}	**Q**		**2b**	**2c**	**2e**	**CHR**
NMF	-1.22<br>(-249 mV)	1.89	4	**N**	17.4	0.78	N.D.	0.045
				**Yield**	34.7	1.6		
				**FE**	83.8	3.8		
**Solvent**	E_{app}	I_{avg}	**Q**		**2b**	**2c**	**2e**	**CHR**
NMP	-1.77<br>(-258 mV)	2.24	4.38	**N**	0.18	20.5	1.1	120.1
				**Yield**	0.36	40.9	4.3	
				**FE**	0.79	90.1	4.7	
**Solvent**	E_{app}	I_{avg}	**Q**		**2b**	**2c**	**2e**	**CHR**
PrN	-1.79<br>(-261 mV)	4.54	3.97	**N**	0.72	19.2	0.72	27.6
				**Yield**	1.4	38.3	2.9	
				**FE**	3.5	93.2	3.5	

E_{app} (V vs. $Me_{10}Fc$): Average applied potential after IR correction. The target voltage was -268 mV of the 2nd cathodic peak from the LSV of 10 mM **2a** in each solvent. Note that the actual applied potentials may deviate from the target potential due to incomplete IR compensation.

I_{avg} (mA/cm^2): Magnitude of the average current density, defined as $I_{avg} = \frac{Q}{t}$, where Q is the total charge passed and t is the time of the experiment.

Q (C): Magnitude of the total charge passed.

N (µmol): Amount of compound measured at the end of the experiment. Note that **2a** could not be reliably quantified by GCMS due to elimination to make styrene. The alcohol side product could also not be accurately quantified because some could form during the workup steps from unreacted **2a**. N.D. = not detected.

Yield (%): Percentage of the initial substrate ending up in each compound, defined as $\frac{m_i N_i}{N_0} \times 100\%$, where N_i is the moles of species *i* at the end of the experiment, m_i is the number of molecules of **2a** needed to form species *i*, and N_0 is the initial moles of **2a** (assumed to be 50 μmol for all experiments).
FE (%): Percentage of the total passed charge going toward a given product. FE is only reported for species produced directly by Faradaic reactions. The formation of product **2b** was assumed to involve two electrons, either directly or indirectly.
CHR: Carboxylation-to-hydrogenolysis ratio, calculated by taking the ratio of the moles of carboxylate products (**2c** and **2e**) to the hydrogenolysis product (**2b**).
N.D. Not Detected

Table S6. Product distribution data for the carboxylation of **1a** at -5 mA/cm^2 for 20 min.

**Solvent**		**1a**	**1b**	**1c**	**1e**	**1d**	**CO**	**H_2**	**MB**	**FE_{tot}**	**CHR**
DMA	**Yield**	81.6 ± 0.6	6.1 ± 0.03	6.2 ± 0.08	0.14 ± 0.04	0.067 ± 8×10^{-3}			94.5	99.1	1.09
	**FE**		42.9 ± 0.4	46.4 ± 0.09	0.48 ± 0.2	0.47 ± 0.06	8.7 ± 0.5	0.07 ± 0.1			
DMF	**Yield**	84.2 ± 3.1	3.4 ± 0.2	7.5 ± 0.6	0.035 ± 0.01	0.067 ± 0.03		N.D.	95.2	86.5	2.22
	**FE**		23.9 ± 1.4	52.9 ± 3.4	0.25 ± 0.08	0.31 ± 0.01	9.1 ± 1.6				
DMI	**Yield**	80.4 ± 0.3	4.7 ± 0.02	8.0 ± 0.1	N.D.	0.084 ± 6×10^{-3}			93.5	97.2	1.73
	**FE**		33.3 ± 0.2	56.8 ± 0.7		0.59 ± 0.04	6.1 ± 0.6	0.36 ± 0.01			
DMSO*	**Yield**	82.0 ± 1.5	5.1 ± 0.03	4.6 ± 0.02	0.038 ± 2×10^{-3}	0.036 ± 8×10^{-5}			94.4	94.8	0.91
	**FE**		35.5 ± 1.5	32.1 ± 1.4	0.26 ± 0.03	0.25 ± 0.01	26.4 ± 2.7	0.14 ± 0.01			
GBL	**Yield**	82.3 ± 0.4	7.9 ± 0.2	6.0 ± 0.1	N.D.	0.23 ± 0.03			96.6	102.4	0.76
	**FE**		55.7 ± 0.8	42.0 ± 0.4		1.6 ± 0.2	0.81 ± 0.2	2.2 ± 0.06			
MeCN	**Yield**	87.5 ± 0.3	7.3 ± 0.3	5.2 ± 0.02	0.017 ± 0.018	0.22 ± 4×10^{-3}			100.2	99.0	0.72
	**FE**		50.5 ± 1.2	36.3 ± 0.55	0.12 ± 0.13	1.5 ± 0.05	10.1 ± 1.3	0.51 ± 0.7			
NMF	**Yield**	85.6 ± 4.6	8.3 ± 2.8	0.04 ± 0.05	0.032 ± 0.05	0.22 ± 0.07			94.5	98.4	5×10^{-3}
	**FE**		59.0 ± 19.7	0.30 ± 0.33	0.23 ± 0.3	1.6 ± 0.49	12.0 ± 8.2	25.5 ± 31.2			
NMP	**Yield**	81.9 ± 0.8	7.4 ± 0.1	6.1 ± 0.2	0.13 ± 9×10^{-3}	0.050 ± 7×10^{-3}			95.8	97.5	0.84
	**FE**		51.8 ± 0.9	42.6 ± 1.2	0.94 ± 0.06	0.35 ± 0.06	1.8 ± 0.07	0.06 ± 0.09			
PrN	**Yield**	79.2 ± 0.7	9.8 ± 0.7	2.8 ± 0.2	N.D.	0.47 ± 0.09			92.8	96.4	0.28
	**FE**		68.7 ± 4.3	19.3 ± 1.2		3.3 ± 0.6	0.45 ± 0.04	4.7 ± 1.4			

N.D. Not Detected

DMSO: 3-phenylpropionaldehyde observed

Table S7. Product distribution data for the carboxylation of **1a** at -1 mA/cm^2 for 1 hr.

Solvent		1a	1b	1c	1e	1d	CO	H_2	MB	FE_{tot}	CHR
DMA	**Yield**	85.2	1.8	4.2	0.044	0.89		N.D.	94.7	100.7	1.09
	**FE**		41.2	44.7	0.26	13.2	1.3				
DMF	**Yield**	89.7	2.3	5.0	0.011	0.18		N.D.	97.2	93.5	2.23
	**FE**		25.0	55.7	0.13	1.0	11.6				
DMI	**Yield**	88.5	3.4	4.7	N.D.	0.38			97.3	101.7	1.38
	**FE**		39.9	55.0		4.6	1.8	0.47			
DMSO*	**Yield**	80.2	1.8	4.2	4.3×10^{-3}	0.89		N.D.	88.0	82.8	2.30
	**FE**		21.7	50.0	0.051	10.5	0.48				
GBL	**Yield**	88.3	4.0	3.9	N.D.	0.68			96.9	100.2	0.99
	**FE**		46.3	46.0		4.0	2.4	1.5			
MeCN	**Yield**	93.0	6.8	0.059	6.8×10^{-3}	0.63	N.D.		101.1	93.5	0.01
	**FE**		78.9	0.69	0.079	7.3		6.6			
NMF	**Yield**	85.1	6.5	4.2×10^{-3}	6.6×10^{-3}	0.18			92.0	88.4	6×10^{-4}
	**FE**		77.2	0.050	0.079	2.1	2.7	6.3			
NMP	**Yield**	87.4	4.1	4.4	0.018	0.090		N.D.	96.0	99.8	1.09
	**FE**		46.7	50.9	0.21	1.0	0.76				
PrN	**Yield**	92.0	3.3	0.023	N.D.	0.17	N.D.		95.6	89.0	7×10^{-3}
	**FE**		38.0	0.27		2.0		48.7			

N.D. Not Detected

DMSO: 3-phenylpropionaldehyde observed

Table S8. DFT-calculated free energies of deprotonation (ΔG_{an}) and hydrogen abstraction (ΔG_{rad}) from solvent molecules (kJ/mol). The following reference reaction is used for deprotonation

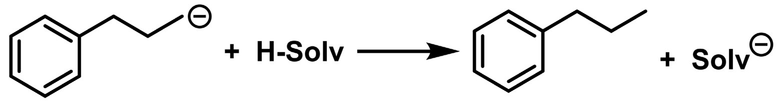

The following reaction is used for hydrogen abstraction

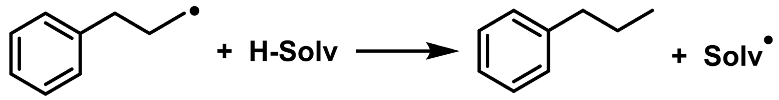

Site	ΔG_{an} M06-2X/def2-TZVPD + PCM	ΔG_{an} B3LYP/6-31++G(d,p) + PCM	ΔG_{an} B3LYP/6-31++G(d,p)	ΔG_{rad} M06-2X/def2-TZVPD + PCM
DMA (PCM solvent = DMA)				
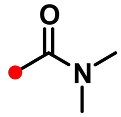	-125.1	-118.1	-132.7	-4.5
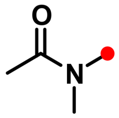	-37.0	-34.1	-37.8	-24.4
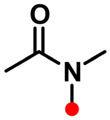	-41.0	-38.0	-67.2	-27.7
DMF (PCM solvent = DMF)				
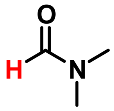	-82.2	-82.6	-82.9	-20.6
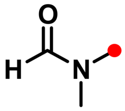	-50.5	-51.1	-54.5	-27.6
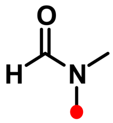	-45.6	-46.2	-66.4	-27.3
DMI (PCM solvent = DMA)				
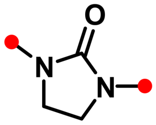	-37.9	-33.6	-39.3	-29.9
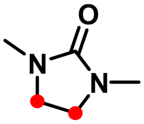	-41.8	-37.5	-65.1	-36.7

DMSO (PCM solvent = DMSO)				
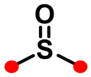	-126.8	-120.2	-137.0	8.92
GBL (PCM solvent = DMA)				
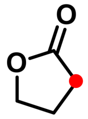	-162.3	-158.2	-174.4	-31.0
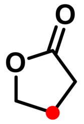	-35.6	--*	--*	-11.6
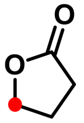	-46.9	-46.3	-66.4	-12.4
MeCN (PCM solvent = MeCN)				
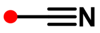	-153.9	-154.4	-161.1	-11.00
NMF (PCM solvent = NMF)				
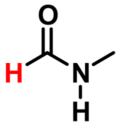	-91.8	-93.3	-94.1	-20.7
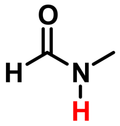	-195.3	-198.2	-199.8	40.6
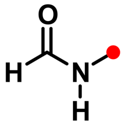	-49.3	-44.9	-67.8	-26.8
NMP (PCM solvent = DMA)				
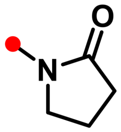	-48.1	-45.4	-56.0	-29.7
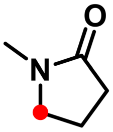	-31.2	-27.0	-55.0	-31.9
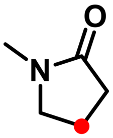	-24.4	-18.0	-39.8	-14.7
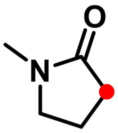	-123.5	-115.5	-137.6	-30.2

PrN (PCM solvent = PrN)				
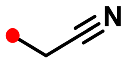	-32.6	-25.7	-38.8	8.14
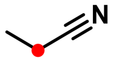	-139.7	-139.0	-151.1	-34.8
Ethylbenzene (**2b**) (PCM solvent = DMA)				
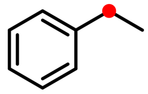	-87.3	–	–	–

5 Geometries of Molecules, Anions, and Radicals

Geometries optimized at the M06-2X/def2-TZVPD level with PCM solvation. Coordinates are in Angstroms. PCM solvents for calculations of solvent molecules are given in **Table S8**.

5.1 DMA

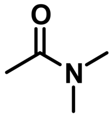

C -0.7199890000 -0.2902970000 0.0000010000
C -1.7605640000 0.8073930000 0.0000000000
H -1.6662940000 1.4409750000 -0.8818840000
H -1.6663030000 1.4409680000 0.8818900000
H -2.7386300000 0.3368690000 -0.0000070000
N 0.5807710000 0.0748530000 0.0000080000
O -1.0666400000 -1.4704880000 -0.0000030000
C 1.0623820000 1.4422750000 -0.0000030000
H 0.2448880000 2.1533720000 0.0000290000
H 1.6780030000 1.6182270000 -0.8849240000
H 1.6780600000 1.6182160000 0.8848800000
C 1.6265250000 -0.9302530000 0.0000000000
H 1.1786470000 -1.9174700000 0.0000180000
H 2.2546330000 -0.8129570000 0.8856550000
H 2.2546030000 -0.8129760000 -0.8856800000

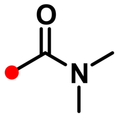

Anion

C 0.8276390000 -0.1048280000 -0.0627940000
O 1.3160870000 -1.2808480000 -0.1420200000
C 1.5527240000 1.0410570000 0.2007720000
H 1.1126520000 2.0192280000 0.3029280000
H 2.6265060000 0.9522340000 0.2966970000
N -0.5809680000 -0.0052510000 -0.2973160000
C -1.3704400000 -1.1038350000 0.2271710000
H -2.3594460000 -1.0973240000 -0.2338490000
H -1.5051360000 -1.0292350000 1.3182820000
H -0.8777640000 -2.0445570000 0.0047670000
C -1.2197630000 1.2742320000 -0.0894270000
H -2.2683770000 1.1985440000 -0.3799420000
H -0.7525000000 2.0406740000 -0.7059280000
H -1.1788240000 1.6042260000 0.9600860000

Radical

C 0.7803880000 -0.1950010000 -0.0204690000
O 1.2392710000 -1.3320030000 -0.0215030000
C 1.6834600000 0.9480230000 0.0365030000
H 1.3670460000 1.9650760000 0.2007080000
H 2.7358720000 0.7214860000 -0.0200950000
N -0.5661630000 0.0507430000 -0.0599130000
C -1.4905550000 -1.0587750000 0.0477430000
H -2.2068220000 -1.0337250000 -0.7762090000
H -2.0439390000 -1.0105960000 0.9902890000
H -0.9274300000 -1.9857010000 0.0103280000
C -1.1345830000 1.3816850000 -0.0213420000
H -2.1659680000 1.3246600000 -0.3662590000
H -0.6041060000 2.0577140000 -0.6898340000
H -1.1379380000 1.8063180000 0.9878830000

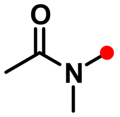

Anion

C 0.6747280000 -0.2660640000 -0.0140490000
O 1.1082890000 -1.4404440000 0.0186130000
C 1.6729170000 0.8800580000 -0.0150760000
H 2.6678540000 0.4456010000 0.0218790000
H 1.5433390000 1.5337630000 0.8489690000
H 1.5945700000 1.4953130000 -0.9123640000
N -0.6270730000 0.0044990000 -0.0248190000
C -1.6466870000 -1.0332600000 0.1326460000
H -2.4878750000 -0.7381250000 -0.5067930000
H -1.2370360000 -1.9658880000 -0.2560520000
C -1.1442740000 1.3543290000 0.0198240000
H -1.9292800000 1.4628190000 -0.7317000000
H -0.3769370000 2.0946940000 -0.1776550000
H -1.5915340000 1.5535100000 0.9984750000

Radical

C 0.7062770000 -0.2706360000 -0.0058920000
O 1.1277740000 -1.4085920000 0.0022380000
C 1.6258440000 0.9274100000 0.0054640000
H 2.6366080000 0.5593630000 0.1507760000
H 1.3732490000 1.6290030000 0.8011610000
H 1.5779030000 1.4650640000 -0.9432780000
N -0.6551560000 -0.0166320000 -0.0274820000
C -1.5376040000 -1.0663440000 0.0151970000
H -2.5892880000 -0.8472600000 -0.0319210000
H -1.1330780000 -2.0612640000 0.0295760000
C -1.1807240000 1.3394390000 0.0080890000
H -2.2067350000 1.3222520000 -0.3513500000
H -0.6055120000 1.9953700000 -0.6403280000
H -1.1719970000 1.7434180000 1.0226930000

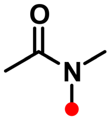

Anion

C 0.6678960000 -0.2812630000 0.0177260000
O 1.0079060000 -1.4905020000 0.0494900000
C 1.7216750000 0.7966490000 -0.0316800000
H 2.6971970000 0.3324120000 -0.1467610000
H 1.7109340000 1.3933380000 0.8828120000
H 1.5259940000 1.4824210000 -0.8591100000
N -0.5927880000 0.1380490000 0.0109650000
C -1.6617960000 -0.8354480000 -0.0283360000
H -2.3013300000 -0.6266000000 -0.8900520000
H -2.2758670000 -0.7579930000 0.8727390000
H -1.2533760000 -1.8377230000 -0.1051080000
C -1.0289650000 1.5390680000 -0.1251480000
H -1.9928700000 1.5949730000 0.3947030000
H -0.3398550000 2.1731030000 0.4398450000

Radical

C 0.6751630000 -0.3259980000 0.0000060000
O 0.8733060000 -1.5249190000 0.0000000000
C 1.8042760000 0.6723520000 -0.0000030000
H 2.7359950000 0.1160800000 -0.0000110000
H 1.7593990000 1.3125380000 0.8824280000
H 1.7593850000 1.3125410000 -0.8824310000
N -0.6074140000 0.1939790000 0.0000000000
C -1.7337000000 -0.7309810000 -0.0000020000
H -2.3434180000 -0.5647680000 -0.8885760000
H -2.3433980000 -0.5648000000 0.8885920000
H -1.3499440000 -1.7448960000 -0.0000240000
C -0.8971870000 1.5334140000 0.0000010000
H -1.9343410000 1.8195400000 -0.0000040000
H -0.1095360000 2.2625340000 0.0000080000

5.2 DMF

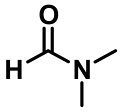

C -0.8561860000 -0.6392370000 -0.0000010000
H -0.7653390000 -1.7350370000 0.0000010000
O -1.9474880000 -0.0858980000 0.0000010000
N 0.3331740000 -0.0234460000 0.0000000000
C 1.5761470000 -0.7658520000 0.0000000000
H 1.3673690000 -1.8335670000 0.0000000000
H 2.1631360000 -0.5190860000 0.8865150000
H 2.1631370000 -0.5190860000 -0.8865140000
C 0.4350680000 1.4213520000 0.0000000000
H -0.5636960000 1.8468710000 -0.0000010000
H 0.9764520000 1.7568180000 -0.8864420000
H 0.9764510000 1.7568180000 0.8864430000

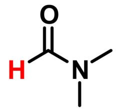

Anion
C 0.8623030000 -0.8207920000 0.0000030000
N -0.3086390000 -0.0656070000 0.0000030000
O 1.9315840000 -0.1593890000 -0.0000030000
C -1.5944760000 -0.7084720000 -0.0000020000
C -0.3414890000 1.3808040000 0.0000000000
H -1.4429560000 -1.7873860000 -0.0000080000
H -2.1829610000 -0.4332410000 -0.8835640000
H -2.1829660000 -0.4332510000 0.8835600000
H -0.8619590000 1.7658250000 0.8838830000
H -0.8619640000 1.7658250000 -0.8838810000
H 0.6825770000 1.7473440000 -0.0000030000

Radical
C 0.8651280000 -0.6432890000 0.0000000000
N -0.3114540000 -0.0489840000 0.0000000000
O 1.9808210000 -0.2013160000 0.0000000000
C -1.5443350000 -0.8162590000 0.0000000000
C -0.4361110000 1.4061420000 0.0000000000
H -1.3038200000 -1.8760550000 -0.0000020000
H -2.1318040000 -0.5761040000 -0.8873020000
H -2.1318010000 -0.5761080000 0.8873050000
H -0.9817070000 1.7273990000 0.8879810000
H -0.9817060000 1.7274000000 -0.8879820000
H 0.5563510000 1.8473170000 0.0000010000

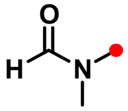

Anion
C 0.8118170000 -0.6113000000 0.0219270000
N -0.3384600000 0.0392790000 0.0106090000
O 1.9420150000 -0.0826370000 -0.0074970000
H 0.7003400000 -1.7048610000 0.0476750000
C -1.5796950000 -0.6993590000 -0.0078980000
C -0.4512370000 1.4793760000 -0.1402770000
H -1.3888040000 -1.7684140000 0.0695780000
H -2.1211670000 -0.4924290000 -0.9349050000
H -2.2109150000 -0.3891270000 0.8281930000
H -1.2944830000 1.7989970000 0.4823950000
H 0.4628150000 1.9296720000 0.2502690000

Radical
C 0.8223290000 -0.6334830000 0.0000190000
N -0.3529520000 0.0581890000 0.0000080000
O 1.9216490000 -0.1040480000 -0.0000120000
H 0.6817250000 -1.7203450000 -0.0000120000
C -1.6238750000 -0.6561550000 -0.0000060000
C -0.3669480000 1.4224300000 0.0000010000
H -1.4358430000 -1.7264310000 -0.0000080000
H -2.1955910000 -0.3919730000 -0.8885250000
H -2.1956100000 -0.3919800000 0.8885040000
H -1.3215380000 1.9189120000 -0.0000120000
H 0.5753020000 1.9401260000 0.0000080000

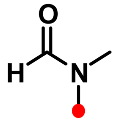

Anion	Radical
C 0.7914730000 -0.6366920000 0.0448780000	C 0.8452340000 -0.6232830000 -0.0105240000
N -0.3941380000 -0.0509960000 0.0228800000	N -0.4005200000 -0.0791310000 -0.0140380000
O 1.9049440000 -0.0804450000 -0.0157230000	O 1.8839370000 0.0156650000 0.0083570000
H 0.7141180000 -1.7329180000 0.1018860000	H 0.8256200000 -1.7209350000 -0.0276930000
C -1.6703930000 -0.7673780000 -0.1391810000	C -1.5266510000 -0.8655450000 0.0255730000
C -0.4648160000 1.3930730000 -0.0126430000	C -0.5552490000 1.3702580000 0.0020300000
H -1.4241810000 -1.8311680000 -0.2360840000	H -1.4073340000 -1.9358690000 0.0173320000
H -2.2240730000 -0.6538970000 0.8054230000	H -2.4839290000 -0.3853850000 -0.0735540000
H -1.2623280000 1.7243670000 0.6538090000	H -0.8486530000 1.7038420000 0.9973240000
H -0.7044660000 1.7382500000 -1.0221980000	H -1.3244390000 1.6526420000 -0.7140510000
H 0.4827630000 1.8218830000 0.3044590000	H 0.3908720000 1.8257260000 -0.2704260000

5.3 DMI

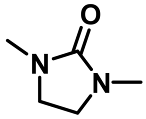

C -0.7577120000 1.4891980000 0.0979050000
N -1.0927650000 0.1073690000 -0.2015290000
C 0.0000000000 -0.6969900000 0.0000030000
N 1.0927660000 0.1073690000 0.2015300000
C 0.7577110000 1.4891980000 -0.0979050000
H 1.0253990000 1.7318800000 -1.1327310000
H 1.2722640000 2.1776020000 0.5700530000
H -1.0254000000 1.7318800000 1.1327310000
H -1.2722640000 2.1776010000 -0.5700530000
O 0.0000010000 -1.9211120000 0.0000000000
C -2.4322120000 -0.3815100000 0.0183140000
H -3.1289750000 0.1509100000 -0.6282560000
H -2.7417380000 -0.2413120000 1.0595550000
H -2.4613250000 -1.4413770000 -0.2219530000
C 2.4322110000 -0.3815100000 -0.0183170000
H 2.4613260000 -1.4413770000 0.2219520000
H 3.1289770000 0.1509120000 0.6282500000
H 2.7417350000 -0.2413130000 -1.0595590000

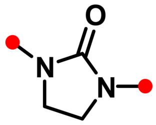

Anion

C 0.7073690000 1.4801550000 -0.0965930000
C -0.8018040000 1.4671860000 0.1238040000
N 1.0682310000 0.1262210000 0.2906710000
H 1.2268330000 2.2253090000 0.5044170000
H 0.9456700000 1.6517070000 -1.1550920000
N -1.1363900000 0.0773710000 -0.1055760000
H -1.3381860000 2.1233060000 -0.5615880000
H -1.0700390000 1.7479340000 1.1504000000
C -0.0507480000 -0.6911540000 0.0511490000
O -0.0006210000 -1.9277600000 0.0457120000
C -2.5212340000 -0.3723360000 0.0384530000
H -3.0782020000 0.0243200000 -0.8223970000
H -2.5052680000 -1.4593490000 -0.0757930000
C 2.3643780000 -0.3598300000 -0.1219310000
H 2.4782880000 -1.3926770000 0.1980440000
H 3.1440710000 0.2431650000 0.3432670000
H 2.4911480000 -0.3109120000 -1.2119120000

Radical

C 0.6670590000 1.4971330000 -0.1174470000
C -0.8469540000 1.4698540000 0.1151320000
N 1.0519110000 0.1378910000 0.2001520000
H 1.1737030000 2.2161690000 0.5257890000
H 0.9057320000 1.7340970000 -1.1619890000
N -1.1583890000 0.0650370000 -0.0651650000
H -1.3949060000 2.0870320000 -0.5960560000
H -1.1036360000 1.7857520000 1.1305880000
C -0.0175780000 -0.7187610000 0.0431790000
O 0.0128540000 -1.9265650000 0.0165340000
C -2.4255260000 -0.4519770000 -0.0318420000
H -2.5140670000 -1.5220170000 -0.1106030000
H -3.2452020000 0.2200850000 -0.2233980000
C 2.3919690000 -0.3253220000 -0.0565070000
H 2.4301070000 -1.3905220000 0.1594270000
H 3.0987750000 0.1997860000 0.5862680000
H 2.6781820000 -0.1639180000 -1.1022970000

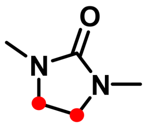

Anion

C -0.7601030000 1.4919940000 0.1447570000
C 0.7408610000 1.6073050000 -0.0721420000
N -1.0852300000 0.1146940000 -0.2660510000
H -1.3337700000 2.2169320000 -0.4355600000
H -1.0513890000 1.5968860000 1.2046510000
N 1.0914400000 0.1810970000 0.1464040000
H 0.8814930000 1.7740650000 -1.1593670000
C 0.0493660000 -0.6527610000 -0.0570560000
O 0.0685450000 -1.8926030000 -0.0570050000
C 2.4477130000 -0.2774620000 0.0781550000
H 2.4826290000 -1.3531510000 0.2431030000
H 3.0475060000 0.2275410000 0.8372670000
H 2.8918670000 -0.0583460000 -0.9012440000
C -2.3731880000 -0.4350680000 0.0756910000
H -2.4037830000 -1.4878210000 -0.1979630000
H -3.1577340000 0.0947600000 -0.4653350000
H -2.5765550000 -0.3446240000 1.1515860000

Radical

C -0.7790600000 1.5132410000 -0.0124810000
C 0.7168330000 1.4991150000 0.0760930000
N -1.0896210000 0.1036330000 0.0896800000
H -1.1488110000 1.9258710000 -0.9653010000
H -1.2540080000 2.0840630000 0.7934250000
N 1.1142070000 0.1837970000 -0.0409020000
H 1.3675420000 2.2921860000 -0.2579870000
C 0.0248650000 -0.6821130000 0.0093910000
O 0.0779490000 -1.8924640000 -0.0095420000
C 2.4741020000 -0.2863220000 0.0031080000
H 2.4550830000 -1.3663100000 -0.1233740000
H 2.9392810000 -0.0398810000 0.9600180000
H 3.0560940000 0.1653220000 -0.8016690000
C -2.4227820000 -0.4184920000 -0.0268650000
H -2.3653420000 -1.5029150000 0.0393430000
H -2.8741850000 -0.1413550000 -0.9845550000
H -3.0550960000 -0.0418530000 0.7795190000

5.4 DMSO

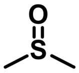

C 0.2597050000 -0.7647530000 1.3458510000
S 0.2597050000 0.4176870000 0.0000000000
H 1.1885950000 -1.3324450000 1.3213100000
H -0.6048370000 -1.4185090000 1.2366480000
H 0.1957670000 -0.1953300000 2.2704490000
C 0.2597050000 -0.7647530000 -1.3458510000
H 1.1885950000 -1.3324450000 -1.3213100000
H 0.1957670000 -0.1953300000 -2.2704490000
H -0.6048370000 -1.4185090000 -1.2366480000
O -1.1038480000 1.0483260000 0.0000000000

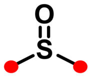

Anion

C 1.3109050000 -0.8115020000 0.1212430000
S -0.1188570000 0.1526850000 -0.4029060000
H 1.3432160000 -1.7523890000 -0.4217950000
H 1.2118870000 -0.9953540000 1.1926710000
H 2.2078840000 -0.2267560000 -0.0731510000
C -1.3593250000 -0.8186120000 0.1916240000
H -1.3239400000 -0.9873210000 1.2700190000
H -2.3273440000 -0.4970830000 -0.1850920000
O 0.1350660000 1.4745790000 0.3483300000

Radical

C 1.5319530000 -0.2728370000 0.1955210000
S -0.1131440000 0.1417860000 -0.4034450000
H 1.8203810000 -1.2342770000 -0.2258220000
H 1.5056790000 -0.3066160000 1.2826450000
H 2.1983270000 0.5101680000 -0.1587180000
C -0.9682890000 -1.2172450000 0.2225940000
H -2.0053920000 -1.0585240000 0.4805630000
H -0.5970860000 -2.2046640000 -0.0127340000
O -0.5616980000 1.3707280000 0.3225620000

5.5 GBL

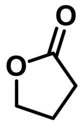

C -1.3981940000 0.6591940000 -0.2255500000
C -1.2630840000 -0.8187250000 0.1309310000
C -0.0294890000 1.2004230000 0.1717220000
H -2.2237410000 1.1312180000 0.3001490000
H -1.5546100000 0.7735840000 -1.2976020000
O 0.1370140000 -1.1193680000 -0.0451540000
H -1.8251370000 -1.4858180000 -0.5160640000
H -1.5195460000 -1.0155200000 1.1723160000
C 0.8750390000 0.0014140000 0.0036690000
H 0.0062220000 1.4864650000 1.2257760000
H 0.3325970000 2.0359430000 -0.4206990000
O 2.0728080000 -0.0280960000 -0.0734090000

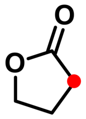

Anion

C 1.4182850000 0.7065310000 0.1215310000
C 1.2478330000 -0.7947630000 -0.1382990000
C 0.0041130000 1.1683970000 -0.1029690000
H 2.1605620000 1.1277350000 -0.5608390000
H 1.7840140000 0.8911870000 1.1415180000
O -0.1201450000 -1.0958260000 0.1271740000
H 1.8730980000 -1.4305220000 0.4882330000
H 1.4550000000 -1.0195320000 -1.1905110000
C -0.8591830000 0.1081910000 -0.0088320000
H -0.3273150000 2.1951450000 -0.1059920000
O -2.1063120000 -0.0159430000 -0.0022990000

Radical

C 1.4142940000 0.7215970000 0.0000100000
C 1.2670570000 -0.8118830000 -0.0000080000
C 0.0127340000 1.1797080000 -0.0000060000
H 1.9592380000 1.0828160000 -0.8761380000
H 1.9591970000 1.0827860000 0.8761980000
O -0.1438870000 -1.0930940000 0.0000100000
H 1.6958990000 -1.2717970000 0.8871370000
H 1.6958710000 -1.2717750000 -0.8871770000
C -0.8788860000 0.0446070000 -0.0000300000
H -0.3375600000 2.1981500000 -0.0000070000
O -2.0890920000 0.0150500000 0.0000130000

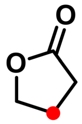

Anion

C 1.3678070000 0.6910970000 -0.4307460000
C 1.3238300000 -0.7225670000 0.1095110000
O -0.0796460000 -1.1103460000 -0.0204430000
H 1.5417800000 -0.8311560000 1.1896300000
H 1.9075440000 -1.4705370000 -0.4277190000
C -0.8280240000 -0.0033120000 0.0037930000
C 0.0608690000 1.2003440000 0.1745490000
H 0.0845420000 1.3843230000 1.2717270000
H -0.4056450000 2.0716910000 -0.2836410000
H 2.2364310000 1.2200660000 -0.0273340000
O -2.0342970000 -0.0606240000 -0.0877200000

Radical

C 1.3772940000 0.7329460000 -0.0000010000
C 1.3516320000 -0.7532690000 0.0000010000
O -0.0435350000 -1.1047250000 0.0000000000
H 1.8108260000 -1.2015420000 0.8853530000
H 1.8108280000 -1.2015440000 -0.8853500000
C -0.8390430000 -0.0230930000 -0.0000050000
C -0.0101910000 1.2449460000 0.0000000000
H -0.2771530000 1.8441260000 0.8768010000
H -0.2771520000 1.8441320000 -0.8767970000
H 2.2781110000 1.3216380000 0.0000040000
O -2.0344160000 -0.1222730000 0.0000020000

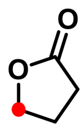

Anion	Radical
C -1.4600040000 0.5307310000 -0.2599020000	C -1.4857940000 0.5578960000 0.1281700000
C -1.3218170000 -0.9569210000 0.0828170000	C -1.2083200000 -0.8861970000 -0.1320400000
C -0.1409470000 1.1492340000 0.1995960000	C -0.1125700000 1.1878560000 -0.1105310000
H -2.3317430000 0.9671240000 0.2298380000	H -1.8114460000 0.7138250000 1.1620770000
H -1.5724900000 0.6903940000 -1.3377450000	H -2.2616340000 0.9558320000 -0.5233080000
O 0.1826530000 -1.1339160000 -0.0884880000	O 0.1469000000 -1.1369340000 -0.0171000000
H -1.4248510000 -1.0222760000 1.1794880000	H -1.8331440000 -1.7234880000 0.1341590000
C 0.8317980000 0.0036340000 0.0073020000	C 0.8481580000 0.0282820000 -0.0004700000
H -0.1578840000 1.3795040000 1.2695720000	H 0.1640850000 1.9692960000 0.5917710000
H 0.2043360000 2.0331770000 -0.3348380000	H -0.0138610000 1.5897240000 -1.1205840000
O 2.0459040000 0.0829160000 -0.0596610000	O 2.0414940000 0.0329080000 0.0727390000

5.6 MeCN

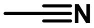

C -1.1753120000 0.0000070000 -0.0000100000
C 0.2804760000 -0.0000250000 0.0000520000
H -1.5394830000 -0.7834720000 -0.6614440000
H -1.5394100000 0.9645810000 -0.3478210000
H -1.5395480000 -0.1810610000 1.0091640000
N 1.4267790000 0.0000080000 -0.0000220000

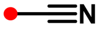

Anion

C -1.1917150000 0.0000000000 -0.0480190000
C 0.1811050000 0.0000000000 0.0005810000
H -1.7183720000 0.9285240000 0.1187220000
H -1.7183720000 -0.9285240000 0.1187220000
N 1.3572010000 0.0000000000 0.0067400000

Radical

C -1.1955540000 -0.0000030000 -0.0000020000
C 0.1944060000 0.0000120000 0.0000000000
H -1.7284060000 0.9374070000 0.0000060000
H -1.7283890000 -0.9374230000 0.0000060000
N 1.3519540000 -0.0000050000 0.0000010000

5.7 NMF

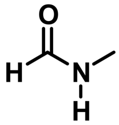

C 0.7160070000 0.3434210000 0.0000090000
O 1.8589260000 -0.0898350000 -0.0000050000
N -0.3892100000 -0.4081750000 0.0000020000
H 0.5034470000 1.4230420000 -0.0000070000
C -1.7391630000 0.1182510000 -0.0000020000
H -2.2813200000 -0.2062680000 0.8872200000
H -2.2813140000 -0.2062690000 -0.8872280000
H -1.6910620000 1.2054600000 -0.0000030000
H -0.2577540000 -1.4100980000 0.0000010000

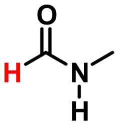

Anion

C -0.7384100000 -0.5084450000 0.0000000000
O -1.8491230000 0.0868080000 0.0000000000
N 0.3498220000 0.3336990000 0.0000010000
C 1.7240270000 -0.1018730000 0.0000000000
H 2.2663610000 0.2433660000 0.8842890000
H 2.2663590000 0.2433620000 -0.8842920000
H 1.7292850000 -1.1913140000 0.0000020000
H 0.1685230000 1.3361360000 0.0000000000

Radical

C -0.7224510000 -0.3312770000 0.0000000000
O -1.8790720000 -0.0210690000 0.0000000000
N 0.3780460000 0.3957450000 -0.0000010000
C 1.7151650000 -0.1736450000 0.0000000000
H 2.2619930000 0.1390580000 0.8879340000
H 2.2620050000 0.1390890000 -0.8879150000
H 1.6231070000 -1.2568710000 -0.0000190000
H 0.2828630000 1.4065840000 0.0000020000

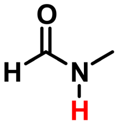

Anion

C 0.6367380000 0.2613230000 0.0000000000
O 1.8578590000 -0.0688730000 0.0000000000
N -0.3910360000 -0.5327740000 0.0000000000
C -1.6940790000 0.0953280000 0.0000000000
H -2.2775820000 -0.2047690000 0.8768760000
H -2.2775830000 -0.2047690000 -0.8768750000
H -1.6442290000 1.1941070000 0.0000000000
H 0.4178170000 1.3559330000 0.0000000000

Radical

C 0.6793430000 0.3196180000 -0.0000010000
O 1.8149360000 -0.1112430000 0.0000020000
N -0.4049420000 -0.5330040000 -0.0000030000
C -1.6833740000 0.1030050000 0.0000010000
H -2.2423300000 -0.2559800000 0.8706870000
H -2.2423410000 -0.2559880000 -0.8706740000
H -1.6525980000 1.1947440000 -0.0000030000
H 0.4765640000 1.4024550000 -0.0000060000

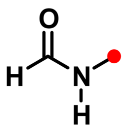

Anion	Radical
C 0.6310590000 0.3382020000 -0.0000480000	C 0.6592960000 0.3600050000 -0.0000070000
O 1.8035770000 -0.0515970000 0.0000210000	O 1.7803630000 -0.1210090000 0.0000060000
N -0.4518140000 -0.4293080000 -0.0000050000	N -0.4765370000 -0.3768490000 -0.0000110000
C -1.8590230000 0.0160000000 0.0000790000	C -1.7547350000 0.1262240000 0.0000200000
H -2.0148910000 0.6506090000 0.8813680000	H -2.5749270000 -0.5672550000 -0.0000420000
H -2.0152300000 0.6499460000 -0.8816320000	H -1.8848550000 1.1947670000 -0.0000060000
H 0.3856330000 1.4130330000 -0.0001400000	H 0.4730380000 1.4422980000 -0.0000160000
H -0.2536400000 -1.4208690000 0.0000930000	H -0.3477690000 -1.3811710000 0.0000130000

5.8 NMP

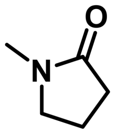

C -1.7749840000 -0.5339580000 -0.1911630000
C -0.5327550000 -1.3751160000 0.1271470000
N 0.5596550000 -0.4311330000 -0.0457780000
H -0.4100160000 -2.2229800000 -0.5472320000
H -0.5421390000 -1.7546280000 1.1540400000
C -1.3367770000 0.8940440000 0.1349820000
H -2.6445430000 -0.8586330000 0.3744050000
H -2.0065960000 -0.6172980000 -1.2525400000
C 0.1738170000 0.8596790000 -0.0043140000
H -1.5598600000 1.1670020000 1.1693530000
H -1.7649410000 1.6565980000 -0.5116350000
C 1.9354920000 -0.8601930000 -0.0141660000
H 2.5732140000 0.0097440000 -0.1488690000
H 2.1250690000 -1.5778350000 -0.8133610000
H 2.1684890000 -1.3334800000 0.9427970000
O 0.9193730000 1.8303380000 -0.0451790000

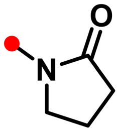

Anion

C -0.2921860000 0.7990270000 -0.0126900000
N -0.5649330000 -0.5007420000 -0.0274770000
C 1.2080360000 0.9988500000 0.1106780000
C 1.7807440000 -0.3847730000 -0.1934630000
H 1.4304170000 1.3213400000 1.1327850000
H 1.5541330000 1.7817350000 -0.5630060000
C 0.6179720000 -1.3222780000 0.1465590000
H 2.6788340000 -0.6207880000 0.3740070000
H 2.0200800000 -0.4641490000 -1.2541520000
H 0.6618980000 -1.6832780000 1.1812670000
H 0.5679080000 -2.1962850000 -0.5058350000
C -1.8763340000 -1.1016180000 0.1034140000
H -1.9094570000 -1.9599950000 -0.5787910000
H -2.6070010000 -0.3653930000 -0.2379210000
O -1.1339590000 1.7195950000 -0.0353750000

Radical

C -0.3289330000 0.8105980000 0.0082040000
N -0.5471440000 -0.5433570000 -0.0031380000
C 1.1586470000 1.0478440000 0.1365830000
C 1.7802900000 -0.3028250000 -0.2198860000
H 1.3641420000 1.3322370000 1.1721310000
H 1.4725140000 1.8693870000 -0.5036090000
C 0.6880320000 -1.3142250000 0.1403770000
H 2.7072200000 -0.5035240000 0.3108360000
H 1.9804140000 -0.3479970000 -1.2898760000
H 0.7758520000 -1.6661410000 1.1706330000
H 0.6648390000 -2.1773750000 -0.5226690000
C -1.7822750000 -1.1120430000 -0.0304230000
H -1.8521130000 -2.1857350000 -0.0441610000
H -2.6353250000 -0.4588080000 -0.0892990000
O -1.2177620000 1.6456700000 -0.0488940000

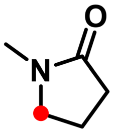

Anion

C -0.1656180000 0.8166900000 -0.0275740000
N -0.5314690000 -0.4616950000 -0.0259820000
C 1.3370280000 0.8441840000 0.1761770000
C 1.7586170000 -0.5757130000 -0.1968090000
H 1.5012910000 1.0289070000 1.2464860000
H 1.8111540000 1.6474150000 -0.3858350000
C 0.5582070000 -1.4158330000 0.2674350000
H 2.7063970000 -0.8657650000 0.2564270000
H 1.8912150000 -0.6056880000 -1.2916250000
H 0.4102740000 -2.2714870000 -0.4045070000
C -1.9056750000 -0.8760810000 0.0110070000
H -2.1588560000 -1.2523200000 1.0078130000
H -2.0755040000 -1.6810690000 -0.7069400000
H -2.5472060000 -0.0317400000 -0.2313300000
O -0.9142280000 1.8130160000 -0.0862520000

Radical

C -0.1257740000 0.8419220000 -0.0079220000
N -0.5626450000 -0.4515780000 0.0067770000
C 1.3871260000 0.8141200000 0.0907130000
C 1.7682830000 -0.6616960000 -0.0967610000
H 1.6542830000 1.1843280000 1.0825810000
H 1.8249950000 1.4872060000 -0.6428700000
C 0.4663890000 -1.3741260000 0.1071390000
H 2.5409450000 -0.9907300000 0.5969300000
H 2.1496150000 -0.8354560000 -1.1085560000
H 0.2596650000 -2.4059920000 -0.1320990000
C -1.9622320000 -0.8173180000 0.0054370000
H -2.2213430000 -1.3134560000 0.9410520000
H -2.1695190000 -1.4916040000 -0.8251000000
H -2.5516600000 0.0887800000 -0.1022970000
O -0.8439030000 1.8275700000 -0.0560900000

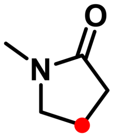

Anion

C -0.0663520000 0.8474920000 -0.0148560000
N -0.5515150000 -0.4032150000 -0.0711900000
C 1.4431210000 0.7451750000 0.1716770000
C 1.8200150000 -0.7043620000 -0.1609550000
H 1.6188910000 1.0207300000 1.2228980000
H 1.9480270000 1.5025480000 -0.4312550000
C 0.5093530000 -1.4156490000 0.1431500000
H 1.9305320000 -0.7426450000 -1.2540060000
H 0.4096270000 -1.7597460000 1.1843210000
H 0.3201970000 -2.2788650000 -0.5012750000
C -1.9508050000 -0.7277170000 -0.0096850000
H -2.1928060000 -1.2088990000 0.9429390000
H -2.2234770000 -1.4121670000 -0.8157510000
H -2.5348650000 0.1860300000 -0.1032770000
O -0.7434400000 1.8807360000 -0.0652810000

Radical

C -0.0608390000 0.8564570000 0.0000000000
N -0.5585670000 -0.3959420000 -0.0000010000
C 1.4579320000 0.7732380000 0.0000000000
C 1.7267840000 -0.6860350000 0.0000000000
H 1.8472490000 1.3025640000 0.8764630000
H 1.8472510000 1.3025640000 -0.8764620000
C 0.4448410000 -1.4466230000 0.0000000000
H 2.6983400000 -1.1499890000 0.0000000000
H 0.3243180000 -2.0898840000 0.8814170000
H 0.3243190000 -2.0898840000 -0.8814170000
C -1.9664030000 -0.7061680000 0.0000000000
H -2.2279540000 -1.2861920000 0.8869400000
H -2.2279550000 -1.2861960000 -0.8869370000
H -2.5258870000 0.2256070000 -0.0000020000
O -0.7204500000 1.8872240000 0.0000000000

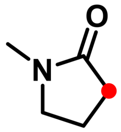

Anion

C -0.2228620000 0.8727350000 -0.0312900000
N -0.4523860000 -0.5171450000 -0.3492250000
C 1.1237810000 1.0582120000 0.1988400000
C 1.8670440000 -0.2232850000 -0.0767290000
H 1.5810740000 2.0363370000 0.2518490000
C 0.7361100000 -1.2448100000 0.0738130000
H 2.6884780000 -0.4461420000 0.6082120000
H 2.2813020000 -0.2742670000 -1.0954030000
H 0.6379520000 -1.5267230000 1.1360210000
H 0.8760950000 -2.1571250000 -0.5097300000
C -1.6992540000 -1.0819110000 0.1084500000
H -1.7314690000 -1.1685280000 1.2073060000
H -1.8325840000 -2.0802900000 -0.3122660000
H -2.5265000000 -0.4516030000 -0.2105130000
O -1.2045690000 1.6753380000 -0.0336750000

Radical

C -0.2201480000 0.8746210000 0.0000030000
N -0.4944250000 -0.4586730000 0.0000080000
C 1.2244950000 1.0039050000 -0.0000020000
C 1.8687820000 -0.3305810000 -0.0000010000
H 1.7299510000 1.9557460000 -0.0000310000
C 0.6787560000 -1.3124430000 0.0000020000
H 2.5088010000 -0.4718040000 0.8748300000
H 2.5088000000 -0.4718030000 -0.8748320000
H 0.6746490000 -1.9556860000 0.8826410000
H 0.6746410000 -1.9556840000 -0.8826370000
C -1.8232260000 -1.0086840000 -0.0000060000
H -1.9820400000 -1.6268000000 0.8865420000
H -1.9820110000 -1.6268160000 -0.8865480000
H -2.5354770000 -0.1873860000 -0.0000250000
O -1.0635360000 1.7737550000 0.0000030000

5.9 PrN

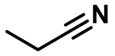

C 1.5395040000 -0.4529290000 0.0000000000
C 0.4854510000 0.6556020000 -0.0000010000
H 1.4407150000 -1.0798420000 -0.8843620000
H 1.4407090000 -1.0798450000 0.8843600000
H 2.5312610000 -0.0052630000 0.0000040000
C -0.8722030000 0.1139820000 0.0000050000
H 0.5869540000 1.2930730000 -0.8787110000
H 0.5869500000 1.2930730000 0.8787090000
N -1.9290140000 -0.3315900000 -0.0000020000

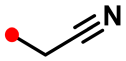

Anion

C 1.5713380000 -0.5455060000 -0.1493470000
C 0.5579690000 0.5966250000 0.0481490000
H 2.5667730000 -0.0830350000 -0.1627100000
H 1.5446740000 -1.1800980000 0.7465120000
C -0.8187060000 0.1034320000 -0.0025620000
H 0.6412510000 1.3337580000 -0.7555660000
H 0.6257950000 1.1704550000 0.9874560000
N -1.8917280000 -0.3097700000 -0.0275900000

Radical

C 1.5694880000 -0.4898120000 -0.0400820000
C 0.5610940000 0.6060750000 0.0410190000
H 2.5817390000 -0.2416500000 -0.3162760000
H 1.3514660000 -1.4650850000 0.3653420000
C -0.8128700000 0.1039230000 -0.0019110000
H 0.6843460000 1.3191390000 -0.7768660000
H 0.6649270000 1.1852670000 0.9677190000
N -1.8841080000 -0.3026830000 -0.0334390000

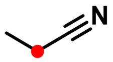

Anion

C 1.6185630000 -0.3383030000 0.0110800000
C 0.4345300000 0.5977670000 -0.0587690000
H 2.4598250000 0.0227050000 -0.5878810000
H 1.3552250000 -1.3218380000 -0.3853870000
H 2.0090220000 -0.5047180000 1.0252560000
C -0.8468350000 0.1209250000 -0.0035320000
H 0.5780080000 1.6459410000 0.1717030000
N -1.9485190000 -0.3034890000 0.0119480000

Radical

C 1.6205510000 -0.3425920000 -0.0000010000
C 0.4510350000 0.5733320000 -0.0000040000
H 2.2450410000 -0.1540880000 -0.8769430000
H 1.3173620000 -1.3863830000 -0.0000850000
H 2.2449440000 -0.1542060000 0.8770360000
C -0.8618280000 0.1097530000 0.0000090000
H 0.5937880000 1.6446620000 -0.0000010000
N -1.9513830000 -0.2847070000 -0.0000040000

5.10 Ethylbenzene

PCM solvent = DMA.

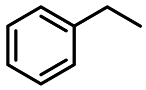

C 1.6214280000 -1.2004260000 0.0951760000
C 2.3061220000 0.0000040000 0.2389160000
C 1.6214210000 1.2004300000 0.0951710000
C 0.2619190000 1.1972310000 -0.1897240000
C -0.4361420000 -0.0000040000 -0.3371880000
C 0.2619250000 -1.1972350000 -0.1897190000
H -0.2676290000 -2.1368300000 -0.3033740000
H 2.1475110000 -2.1407870000 0.2019590000
H 3.3661140000 0.0000080000 0.4579490000
H 2.1475000000 2.1407950000 0.2019500000
H -0.2676410000 2.1368230000 -0.3033830000
C -1.9199250000 -0.0000080000 -0.5938230000
C -2.7175950000 0.0000060000 0.7117190000
H -2.4776980000 -0.8825280000 1.3068980000
H -3.7901400000 0.0000030000 0.5154240000
H -2.4776990000 0.8825520000 1.3068790000
H -2.1876160000 0.8787470000 -1.1837440000
H -2.1876150000 -0.8787760000 -1.1837260000

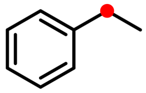

Anion

C -1.8946190000 -0.9627960000 0.0000000000
C -2.3115740000 0.3819390000 0.0000000000
C -1.3095920000 1.3556010000 0.0000000000
C 0.0353860000 1.0341080000 0.0000010000
C 0.4991210000 -0.3355830000 0.0000010000
C -0.5670210000 -1.3136410000 0.0000000000
H -2.6418380000 -1.7513610000 0.0000000000
H -3.3600500000 0.6475990000 -0.0000010000
H -0.2935520000 -2.3650560000 0.0000000000
H -1.5903740000 2.4053820000 0.0000000000
H 0.7665910000 1.8345320000 0.0000010000
C 1.8365340000 -0.6760760000 -0.0000010000
C 2.9285210000 0.3541540000 0.0000000000
H 2.1123050000 -1.7251300000 0.0000010000
H 2.8964090000 1.0220540000 -0.8743710000
H 3.9135560000 -0.1163080000 -0.0000070000
H 2.8964170000 1.0220460000 0.8743780000

5.11 *n*-Propylbenzene

PCM solvent = DMA

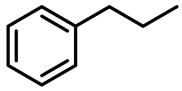

C -2.8739700000 0.3305000000 -0.0000010000
C -2.4481980000 -0.9944170000 -0.0000010000
C -1.9311190000 1.3466720000 0.0000000000
C -0.5715120000 1.0457870000 0.0000010000
C -0.1321170000 -0.2748820000 0.0000010000
C -1.0939760000 -1.2882280000 0.0000000000
C 1.3312100000 -0.6536360000 0.0000020000
C 2.3256780000 0.4986770000 -0.0000010000
H 1.5227640000 -1.2884160000 -0.8714530000
H 1.5227640000 -1.2884120000 0.8714600000
C 3.7640330000 -0.0032140000 -0.0000010000
H 2.1573870000 1.1300840000 0.8765160000
H 2.1573860000 1.1300810000 -0.8765200000
H 3.9610410000 -0.6161490000 0.8817330000
H 4.4741470000 0.8238950000 -0.0000030000
H 3.9610400000 -0.6161520000 -0.8817340000
H -0.7682070000 -2.3228620000 0.0000000000
H -3.1742450000 -1.7977490000 -0.0000020000
H -3.9307470000 0.5644930000 -0.0000010000
H -2.2495260000 2.3816680000 0.0000000000
H 0.1460220000 1.8559570000 0.0000020000

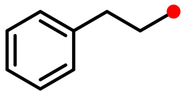

Anion

C 2.8155700000 0.3310390000 0.0830630000
C 2.3881930000 -0.9925620000 0.0821110000
C 1.8741910000 1.3472050000 0.0008940000
C 0.5181800000 1.0457790000 -0.0832890000
C 0.0736590000 -0.2753210000 -0.0889450000
C 1.0348810000 -1.2856740000 -0.0007200000
C -1.3880450000 -0.6417550000 -0.2096080000
C -2.4065160000 0.4619750000 0.0619820000
H -1.5885050000 -1.4779110000 0.4693940000
H -1.5655370000 -1.0399200000 -1.2150620000
C -3.8445240000 -0.0551540000 0.0205180000
H -2.2728970000 1.2485130000 -0.6912370000
H -2.1129140000 0.9325530000 1.0199230000
H -4.5379490000 0.7820370000 0.1875770000
H -3.9982600000 -0.7410330000 0.8688890000
H 0.7091530000 -2.3204840000 0.0034590000
H 3.1110920000 -1.7962690000 0.1494070000
H 3.8703030000 0.5652720000 0.1503730000
H 2.1936260000 2.3821910000 0.0045000000
H -0.2016470000 1.8518600000 -0.1432570000

Radical

C 2.8085010000 0.3389850000 0.0187370000
C 2.3894810000 -0.9879870000 0.0066150000
C 1.8608710000 1.3505700000 0.0117150000
C 0.5028880000 1.0432450000 -0.0074120000
C 0.0705840000 -0.2796700000 -0.0197940000
C 1.0369750000 -1.2885720000 -0.0122260000
C -1.3891270000 -0.6686710000 -0.0461650000
C -2.3890130000 0.4812090000 0.0079930000
H -1.5834750000 -1.3441660000 0.7926450000
H -1.5765520000 -1.2582650000 -0.9488440000
C -3.7949650000 0.0016550000 0.0143630000
H -2.2323110000 1.1600140000 -0.8360090000
H -2.1924660000 1.0838510000 0.9077520000
H -4.6063860000 0.6648210000 -0.2477450000
H -4.0424460000 -0.9547570000 0.4551150000
H 0.7162910000 -2.3246840000 -0.0208820000
H 3.1193850000 -1.7877360000 0.0125260000
H 3.8639840000 0.5781520000 0.0338790000
H 2.1739960000 2.3870720000 0.0212880000
H -0.2171940000 1.8511110000 -0.0126760000

6 References

(1) Armarego, W. L. F. *Purification of Laboratory Chemicals*, 9th ed.; Elsevier: Amsterdam, 2017.

(2) Zeyer, C.; Grüniger, H. R.; Dossenbach, O. A Well-Defined Reference Electrode in Acetonitrile. *J. Appl. Electrochem. 1992 223* **1992**, *22* (3), 304–306.

(3) Coetzee, J. F.; Gardner, C. W. Exchange Current Densities and Other Properties of Reference Electrodes Based on the Triiodide Iodide and Silver(I) Ion-Silver Couples in Organic Solvents. *Anal. Chem.* **2002**, *54* (14), 2530–2532.

(4) Izutsu, K. Reference Electrodes for Use in Nonaqueous Solutions. In *Handbook of Reference Electrodes*; Inzelt, G., Lewnestam, A., Sholz, F., Eds.; 2013; pp 145–188.

(5) Noviandri, I.; Brown, K. N.; Fleming, D. S.; Gulyas, P. T.; Lay, P. A.; Masters, A. F.; Phillips, L. The Decamethylferrocenium/Decamethylferrocene Redox Couple: A Superior Redox Standard to the Ferrocenium/Ferrocene Redox Couple for Studying Solvent Effects on the Thermodynamics of Electron Transfer. **1999**.

(6) Aranzaes, J. R.; Daniel, M. C.; Astruc, D. Metallocenes as References for the Determination of Redox Potentials by Cyclic Voltammetry — Permethylated Iron and Cobalt Sandwich Complexes, Inhibition by Polyamine Dendrimers, and the Role of Hydroxy-Containing Ferrocenes. *https://doi.org/10.1139/v05-262* **2011**, *84* (2), 288–299.

(7) Corbin, N.; Yang, D.-T.; Lazouski, N.; Steinberg, K.; Manthiram, K. Suppressing Carboxylate Nucleophilicity with Inorganic Salts Enables Selective Electrocarboxylation without Sacrificial Anodes. *Chem. Sci.* **2021**, *12* (37), 12365–12376.

(8) Frisch, M. J.; Trucks, G. W.; Schlegel, H. B.; Scuseria, G. E.; Robb, M. A.; Cheeseman, J. R.; Scalmani, G.; Barone, V.; Petersson, G. A.; Nakatsuji, H.; Li, X.; Caricato, M.; Marenich, A. V; Bloino, J.; Janesko, B. G.; Gomperts, R.; Mennucci, B.; Hratchian, H. P.; Ortiz, J. V; Izmaylov, A. F.; Sonnenberg, J. L.; Williams-Young, D.; Ding, F.; Lipparini, F.; Egidi, F.; Goings, J.; Peng, B.; Petrone, A.; Henderson, T.; Ranasinghe, D.; Zakrzewski, V. G.; Gao, J.; Rega, N.; Zheng, G.; Liang, W.; Hada, M.; Ehara, M.; Toyota, K.; Fukuda, R.; Hasegawa, J.; Ishida, M.; Nakajima, T.; Honda, Y.; Kitao, O.; Nakai, H.; Vreven, T.; Throssell, K.; Montgomery Jr., J. A.; Peralta, J. E.; Ogliaro, F.; Bearpark, M. J.; Heyd, J. J.; Brothers, E. N.; Kudin, K. N.; Staroverov, V. N.; Keith, T. A.; Kobayashi, R.; Normand, J.; Raghavachari, K.; Rendell, A. P.; Burant, J. C.; Iyengar, S. S.; Tomasi, J.; Cossi, M.; Millam, J. M.; Klene, M.; Adamo, C.; Cammi, R.; Ochterski, J. W.; Martin, R. L.; Morokuma, K.; Farkas, O.; Foresman, J. B.; Fox, D. J. Gaussian 16, Revision C.01. Gaussian Inc. Wallingford CT 2016.

(9) Zhao, Y.; Truhlar, D. G. The M06 Suite of Density Functionals for Main Group Thermochemistry, Thermochemical Kinetics, Noncovalent Interactions, Excited States, and Transition Elements: Two New Functionals and Systematic Testing of Four M06-Class Functionals and 12 Other Function. *Theor. Chem. Acc.* **2008**, *120* (1–3), 215–241.

(10) Weigend, F.; Ahlrichs, R. Balanced Basis Sets of Split Valence, Triple Zeta Valence and Quadruple Zeta Valence Quality for H to Rn: Design and Assessment of Accuracy. *Phys. Chem. Chem. Phys.* **2005**, *7* (18), 3297–3305.

(11) Rappoport, D.; Furche, F. Property-Optimized Gaussian Basis Sets for Molecular Response Calculations. *J. Chem. Phys.* **2010**, *133* (13), 134105.

(12) St. John, P. C.; Guan, Y.; Kim, Y.; Etz, B. D.; Kim, S.; Paton, R. S. Quantum Chemical Calculations for over 200,000 Organic Radical Species and 40,000 Associated Closed-Shell Molecules. *Sci. Data* **2020**, *7* (1), 1–6.

(13) Pritchard, B. P.; Altarawy, D.; Didier, B.; Gibson, T. D.; Windus, T. L. New Basis Set Exchange: An Open, Up-to-Date Resource for the Molecular Sciences Community. *J. Chem. Inf. Model.* **2019**, *59* (11), 4814–4820.

(14) Becke, A. D. Density-Functional Thermochemistry. III. The Role of Exact Exchange. *J. Chem. Phys.* **1993**, *98* (7), 5648–5652.

(15) Hehre, W. J.; Ditchfield, K.; Pople, J. A. Self-Consistent Molecular Orbital Methods. XII. Further Extensions of Gaussian-Type Basis Sets for Use in Molecular Orbital Studies of Organic Molecules. *J. Chem. Phys.* **1972**, *56* (5), 2257–2261.

(16) Krishnan, R.; Binkley, J. S.; Seeger, R.; Pople, J. A. Self-Consistent Molecular Orbital Methods. XX. A Basis Set for Correlated Wave Functions. *J. Chem. Phys.* **1980**, *72* (1), 650–654.

(17) Frisch, M. J.; Pople, J. A.; Binkley, J. S. Self-Consistent Molecular Orbital Methods 25. Supplementary Functions for Gaussian Basis Sets. *J. Chem. Phys.* **1984**, *80* (7), 3265–3269.

(18) Miertuš, S.; Scrocco, E.; Tomasi, J. Electrostatic Interaction of a Solute with a Continuum. A Direct Utilizaion of AB Initio Molecular Potentials for the Prevision of Solvent Effects. *Chem. Phys.* **1981**, *55* (1), 117–129.

(19) Bard, A. J.; Faulkner, L. R. *Electrochemical Methods: Fundamentals and Applications*, 2nd ed.; John Wiley & Sons, Inc.: New York, 2001.

(20) Falciola, L.; Mussini, P. R.; Trasatti, S.; Doubova, L. M. Specific Adsorption of Bromide and Iodide Anions from Nonaqueous Solutions on Controlled-Surface Polycrystalline Silver Electrodes. *J. Electroanal. Chem.* **2006**, *593* (1–2), 185–193.

(21) Jouikov, V.; Simonet, J. Novel Method for Grafting Alkyl Chains onto Glassy Carbon. Application to the Easy Immobilization of Ferrocene Used as Redox Probe. *Langmuir* **2012**, *28* (1), 931–938.

(22) Koefoed, L.; Pedersen, S. U.; Daasbjerg, K. Covalent Modification of Glassy Carbon Surfaces by Electrochemical Grafting of Aryl Iodides. *Langmuir* **2017**, *33* (13), 3217–3222.

(23) Richard, J. P.; Williams, G.; O'Donoghue, A. M. C.; Amyes, T. L. Formation and Stability of Enolates of Acetamide and Acetate Anion: An Eigen Plot for Proton Transfer at α-Carbonyl Carbon. *J. Am. Chem. Soc.* **2002**, *124* (12), 2957–2968.

(24) Marcus, Y. The Properties of Organic Liquids That Are Relevant to Their Use as Solvating Solvents. *Chem. Soc. Rev.* **1993**, *22* (6), 409–416.

(25) Rinaldi, R. Instantaneous Dissolution of Cellulose in Organic Electrolyte Solutions. *Chem. Commun.* **2011**, *47* (1), 511–513.

(26) Bordwell, F. G. Equilibrium Acidities in Dimethyl Sulfoxide Solution. *Acc. Chem. Res.* **1988**, *21* (12), 456–463.

(27) Kamlet, M. J.; Abboud, J. L. M.; Abraham, M. H.; Taft, R. W. Linear Solvation Energy Relationships. 23. A Comprehensive Collection of the Solvatochromic Parameters, π, α, and β, and Some Methods for Simplifying the Generalized Solvatochromic Equation. *J. Org. Chem.* **1983**, *48* (17), 2877–2887.

(28) Minnick, D. L.; Flores, R. A.; Destefano, M. R.; Scurto, A. M. Cellulose Solubility in Ionic Liquid Mixtures: Temperature, Cosolvent, and Antisolvent Effects. *J. Phys. Chem. B* **2016**, *120* (32), 7906–7919.

(29) Duereh, A.; Sato, Y.; Smith, R. L.; Inomata, H. Analysis of the Cybotactic Region of Two Renewable Lactone-Water Mixed-Solvent Systems That Exhibit Synergistic Kamlet-Taft Basicity. *J. Phys. Chem. B* **2016**, *120* (19), 4467–4481.

(30) Dubouis, N.; Serva, A.; Berthin, R.; Jeanmairet, G.; Porcheron, B.; Salager, E.; Salanne, M.; Grimaud, A. Tuning Water Reduction through Controlled Nanoconfinement within an Organic Liquid Matrix. *Nat. Catal.* **2020**, 1–8.

(31) Gennaro, A.; Isse, A. A.; Vianello, E. Solubility and Electrochemical Determination of CO2 in Some Dipolar Aprotic Solvents. *J. Electroanal. Chem. Interfacial Electrochem.* **1990**, *289* (1–2), 203–215.

(32) Gennaro, A.; Isse, A. A.; Severin, M.-G.; Vianello, E.; Bhugun, I.; Savéant, J.-M. Mechanism of the Electrochemical Reduction of Carbon Dioxide at Inert Electrodes in Media of Low Proton Availability. *J. Chem. Soc. - Faraday Trans.* **1996**, *92* (20), 3963–3968.
